# Human Proteoglycan Linkage Region Glycosyltransferases are Dimeric and Show Unexpected Specificities

**DOI:** 10.1002/anie.202516855

**Published:** 2025-11-29

**Authors:** Sascha Weidler, Ole Bundgaard, Markus Hessefort, Marisa Rädisch, Christopher Günther Franz Graf, Kevin Lam, Vanessa J. Neubauer, Johanna Eisenreich, Leonhard Köhler, Kelley W. Moremen, Catharina Steentoft, Henrik Clausen, Teng‐Yi Huang, Shang‐Cheng Hung, Clemens Steegborn, Michael Weyand, Carlo Unverzagt

**Affiliations:** ^1^ University of Bayreuth Bioorganic Chemistry Universitätsstraße 30 95447 Bayreuth Germany; ^2^ Complex Carbohydrate Research Center University of Georgia Athens GA 30602 USA; ^3^ Copenhagen Center for Glycomics Departments of Cellular and Molecular Medicine and School of Dentistry University of Copenhagen Denmark; ^4^ Genomics Research Center Academia Sinica Taipei Taiwan; ^5^ Department of Biochemistry University of Bayreuth 95447 Bayreuth Germany

**Keywords:** Enzymes, Glycoconjugates, Glycopeptides, Protein structures, Proteoglycan

## Abstract

We selected the *N*,*O*‐glycosylated proteoglycan bikunin as a model to establish a chemoenzymatic approach to defined proteoglycans using native chemical ligation. Overexpression of the human linkage region glycosyltransferases B4GalT7, B3GalT6 and B3GlcAT‐1 as *N*‐terminal SUMO‐fusions gave high yields of soluble and active enzymes in *E. col* *i*. When starting with xylosylated bikunin peptides the transferases performed well in enzymatic cascade reactions and provided the desired linkage region tetrasaccharide glycopeptides. B3GalT6 and B3GlcAT‐1 led to side products with *N*,*O*‐glycosylated bikunin peptides revealing unexpected promiscuity of both enzymes towards complex type *N*‐glycans. Additionally, B3GalT6 was found to synthesize short poly‐β3 Gal structures. B3GlcAT‐1 can slowly convert the biosynthetic intermediate Gal‐Xyl to the non‐canonical trisaccharide GlcA‐Gal‐Xyl. This reaction independently confirmed the recently detected biosynthetic bypass to GAGs in the case of dysfunctional B3GalT6 (spondylodysplastic Ehlers‐Danlos‐syndrome). The three linkage region glycosyltransferases B4GalT7, B3GalT6 and B3GlcAT‐1 were dimeric in solution and the crystal structure of B3GalT6 was solved showing a covalent dimer linked by a disulfide in the center of the large dimerization domain. This motif appears to be conserved in higher organisms and reinforces the concept of dimeric glycosyltransferases lining the Golgi.

## Graphical abstract

**Non‐canonical glycopeptides** of the proteoglycan linkage region are accessible by the enzymes B3GalT6 and GlcAT‐1 confirming a recently discovered rescue mode in glycosaminoglycan (GAG) biosynthesis. The crystal structure of B3GalT6 revealed a covalent dimer linked by a disulfide.

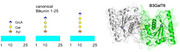

## Introduction

Proteoglycans consist of a protein core and one or more glycosaminoglycan polysaccharide chains. In the case of chondroitinsulfate (CS), dermatansulfate (DS) and heparansulfate (HS) serine moieties of the core protein are modified with a canonical tetrasaccharide sequence termed the linkage region. This tetrasaccharide can be elongated to the various sulfated glycosaminoglycans CS, DS and HS. Proteoglycans are essential for the stability and the functionality of the extracellular matrix.^[^ ^1^ ^]^ Alike glycoproteins the carbohydrate structures of proteoglycans are heterogenous, and the biological activities of both classes of glycoconjugates are modulated by their glycans. Thus, the availability of defined glycoforms of proteoglycans is a requisite for better understanding the formation and the function of proteoglycans. The synthesis of homogenous glycoproteins is well‐established,^[^ ^2^ ^]^ whereas methodologies for proteoglycans are in an early phase.^[^ ^3^, ^4^, ^5^, ^6^, ^7^, ^8^, ^9^, ^10^, ^11^ ^]^ To close this gap we selected bikunin (147 residues) as a model proteoglycan, which contains only one CS chain at Ser 10 and one biantennary *N*‐glycan at Asn 45. Bikunin acts as a protease inhibitor via its two Kunitz domains and is an approved therapeutic against acute inflammatory states. Bikunin can be modified by two heavy chain proteins via esterification of the 6‐OH of GalNAc in the CS oligosaccharide chain. This covalent complex (inter‐α‐trypsin inhibitor) serves to transfer the heavy chains to hyaluronic acid (HA),^[^ ^12^ ^]^ which is increased in rheumatoid arthritis. The same modification of HA is required for the maturation of mammalian oocytes within the preovulatory follicle otherwise leading to infertility.^[^ ^13^ ^]^ For bikunin the glycan composition was studied revealing that the CS chain has a defined sequence.^[^ ^14^ ^]^

**Scheme 1:**
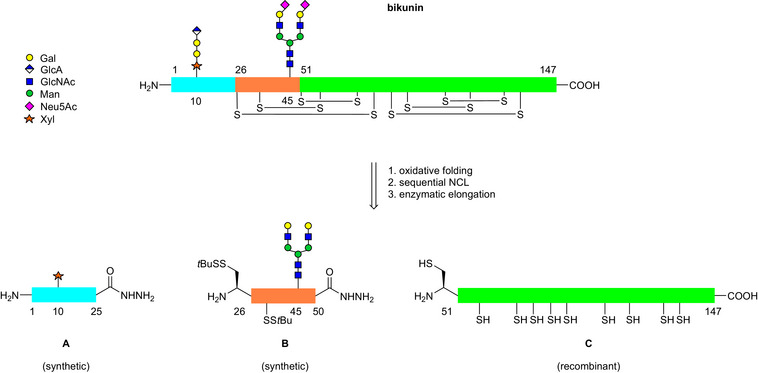
Retrosynthesis of *N*.*O*‐glycosylated bikunin 1–147 with a complex *N*‐glycan at Asn 45 and a linkage region tetrasaccharide at Ser 10.

The polypeptide chain of human bikunin contains twelve cysteines and thus can be readily disconnected into three segments for assembly by sequential native chemical ligation^[^ ^15^ ^]^ (Scheme 1). We set out for a semisynthetic strategy^[^ ^16^ ^]^ based on recombinantly expressed bikunin Cys‐peptide 51–147 **C** in conjunction with synthetic *N*‐glycopeptide 26–50 **B** and *O*‐glycopeptide 1–25 **A**. To ease the accessibility of bikunin 1–25 *O*‐glycopeptides bearing the GAG linkage region tetrasaccharide, a chemoenzymatic approach^[^ ^17^ ^]^ employing glycosyltransferases was envisioned. The glycopeptide substrates were functionalized with the hydrolytically stable hydrazides serving as latent thioesters.^[^ ^18^ ^]^ The glycosyltransferases assembling the linkage region of GAGs should be applicable not only to glycopeptide building blocks but also to the folded proteoglycan bikunin.^[^ ^19^ ^]^ Once established a robust enzymatic methodology is expected to provide access to numerous proteoglycans.

## Results and Discussion

### Glycopeptide Synthesis

**Scheme 2:**
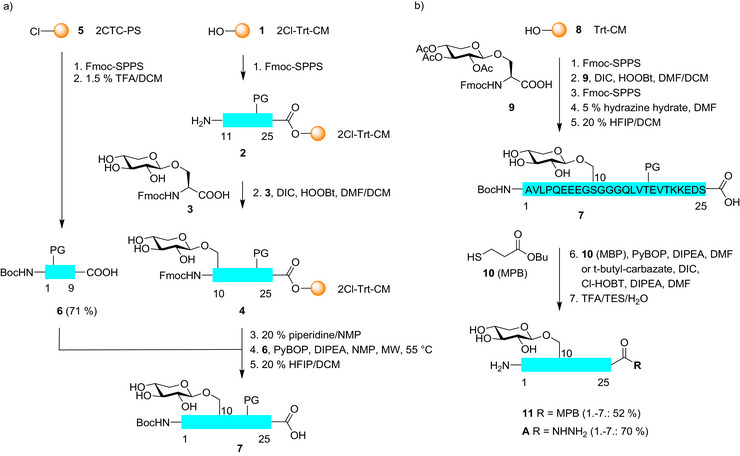
Synthesis of bikunin 1–25 glycopeptides by a) segment condensation and b) stepwise Fmoc‐SPPS.

We started with the preparation of the bikunin 1–25 *O*‐glycopeptide **A** using Fmoc‐solid phase synthesis (Fmoc‐SPPS). The first approach was based on the Fmoc‐Ser(Xyl)‐OH building block **3** (Scheme 2). The xylose moiety of **3** was unprotected to avoid a deacetylation step of the glycopeptide and to prevent unwanted acylation of the *N*‐terminus during chain elongation. However, free hydroxyl groups may lead to esterification of the carbohydrate by activated amino acids.^[^ ^20^ ^]^ This esterification can be minimized by an elongation of the glycopeptide with a protected peptide acid (segment coupling).^[^ ^21^ ^]^ The peptide bikunin 11–25 (**2**) was assembled on the hydrophilic 2‐Cl‐trityl‐ChemMatrix resin **1** employing pseudoproline dipeptides at Thr 17 and 20.^[^ ^22^ ^]^

Elongation with the Fmoc‐Ser(Xyl)‐OH building block **3** (for synthesis: see Supporting Information) to glycopeptide resin **4** unexpectedly gave two isobaric peaks in the LC‐MS of the cleaved 10–25 glycopeptide **4d**. The Gildersleeve group found that glycosylated serine building blocks readily epimerize at the α‐carbon during peptide synthesis^[^ ^23^ ^]^ and thus we tested milder coupling reagents. The isobaric glycopeptide could be lowered from 24% (using the symmetric anhydride) to about 1% using HOOBt^[^ ^24^ ^]^ as the additive (see Supporting Information). Since the stepwise elongation of resin **4** containing the unprotected xylose yielded further isobaric 1–25 glycopeptides (data not shown) the peptide was readily completed by a microwave assisted segment coupling^[^ ^21^ ^]^ using the protected bikunin 1–9 peptide acid **6** (*C*‐terminal glycine). The resulting 1–25 glycopeptide acid **7** was devoid of isobaric side products.

For comparison we also investigated the known peracetylated xylosyl‐serine **9**,^[^ ^24^ ^]^ which was obtained by a modified procedure (supplementary information). During Fmoc‐SPPS on trityl‐CM‐resin **8** coupling of the acetylated building block **9** ^[^ ^25^ ^]^ with DIC/HOOBt proceeded without racemization and was compatible with the subsequent elongation to the 1–25 glycopeptide. The acetyl groups of the xylose moiety were conveniently removed with 5% hydrazine hydrate in DMF on the solid phase.^[^ ^26^ ^]^ After cleavage from the resin the glycopeptide acid **7** was converted to thioester^[^ ^20^ ^]^ **11** and hydrazide^[^ ^18^ ^]^ **A**. Notably, the synthesis of **7** via **9** employing two pseudoproline dipeptides gave similar results using 2‐chlorotrityl‐polystyrene resin.^[^ ^27^ ^]^

**Scheme 3:**
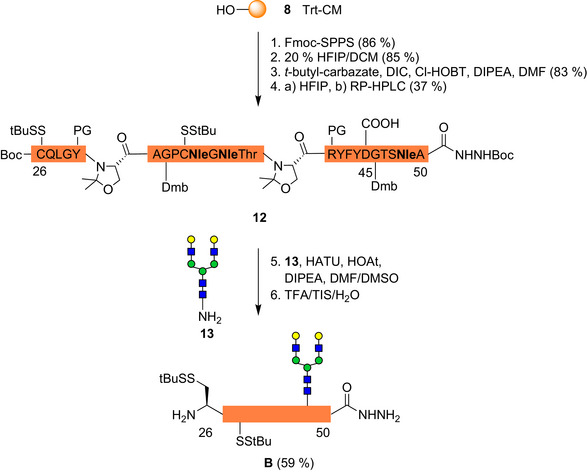
Synthesis of selectively deprotected bikunin 26–50 hydrazide **12** and *N*‐glycopeptide hydrazide **B** by Lansbury aspartylation.

The synthesis of the bikunin 26–50 *N*‐glycopeptide required special efforts and was thus carried out on trityl‐ChemMatrix resin **8** (Scheme 3). The initial approach used an allyl ester at Asp and a pseudoproline in the Asp40‐Gly‐Thr glycosylation sequon.^[^ ^28^, ^29^ ^]^ Despite the flanking pseudoproline the Asp(OAll)‐Gly sequence showed substantial aspartimide formation.^[^ ^29^ ^]^ This was abolished by incorporation of a Dmb‐glycine next to the *N*‐glycosylation site and an Asp 40 PhiPr‐ester protection.^[^ ^30^ ^]^ The three native methionines were replaced by norleucine.^[^ ^31^ ^]^ Additionally, two pseudoproline dipeptides and a second Dmb‐glycine were incorporated to prevent aggregation of the growing peptide chain.

After cleavage of the peptide from the resin with 20% HFIP the *C*‐terminal hydrazide was installed followed by selective removal of the PhiPr‐ester with neat HFIP. The resulting aspartylpeptide **12** was purified by flash chromatography or RP‐HPLC. Lansbury aspartylation^[^ ^32^ ^]^ of **12** with the glycosyl amine **13** ^[^ ^29^ ^]^ resulted in a significantly higher yield when using the peptide purified by HPLC (59% versus 45%).

### Enzymatic Elongations

**Scheme 4:**
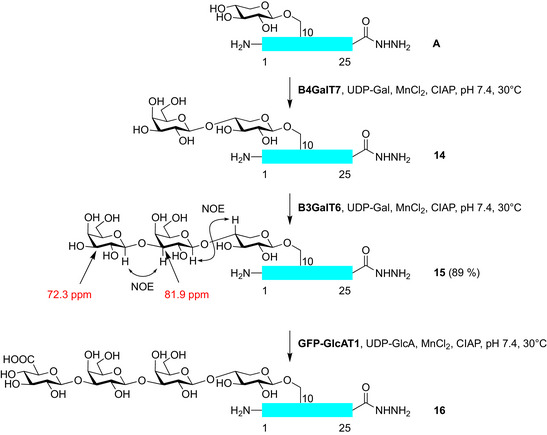
Enzymatic elongation of bikunin 1–25 Xyl‐glycopeptide hydrazide **A** to glycopeptide **16** containing the linkage region tetrasaccharide.

At this point we wanted to establish the enzymatic elongation of the xylose residue on the 1–25 glycopeptide. The Clausen group who initially cloned the human enzyme B4GalT7^[^ ^33^ ^]^ provided some of the enzyme expressed in insect cells. Surprisingly, the enzymatic galactosylation of **A** using recombinant B4GalT7 and alkaline phosphatase^[^ ^34^ ^]^ yielded glycopeptide **14** as a minor product but mainly a glycopeptide with an additional galactose (**15**) (Scheme 4).

Since the enzyme was overexpressed in a eukaryotic host, we suspected that inadvertently the biosynthetically following galactosyltransferase B3GalT6 from the insect cells might have been copurified. This appeared to be the case since after a preparative synthesis the glycan part of the purified glycopeptide **15** was assigned by 2D‐NMR experiments (HSQC and NOESY) and compared to reference data.^[^ ^35^, ^36^ ^]^ The downfield shift of C‐3 of the internal galactose is indicative for a glycosylation at O‐3. Further evidence resulted from NOEs between the anomeric protons of both galactoses and the ring protons of the neighboring residues (details in Supporting Information). Additional (albeit indirect) evidence for the correct assembly of the linkage trisaccharide was deduced by the enzymatic glucuronylation of glycopeptide **15** on an analytical scale with recombinant human glucuronyltransferase‐GFP fusion protein (GFP‐GlcAT1) provided by the Moremen group.^[^ ^37^ ^]^ At this point we could not determine if the copurification of B4GalT7 with B3GalT6 was a mere coincidence or the result of a specific association of both enzymes withstanding the purification conditions.^[^ ^33^ ^]^

### Expression of Linkage Region Transferases in *E. Coli*

Since the activity of the three enzymes expressed in eukaryotic systems was decreasing during storage, we looked for alternative sources. Overexpression of the human transferases B4GalT7^[^ ^38^ ^]^ and B3GlcAT1^[^ ^39^ ^]^ in *E. coli* was reported to provide the active enzymes despite the lack of *N*‐glycosylation and both proteins were crystallized. For B3GalT6 only expressions in mammalian cells were reported.^[^ ^9^, ^35^ ^]^

**Scheme 5:**
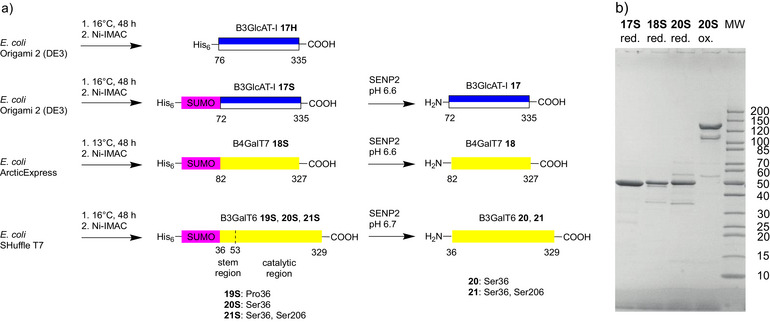
a) Expression of the glycosyltransferases B3GlcAT1, B4GalT7 and B3GalT6 as soluble SUMO fusion proteins (**17S**, **18S**, **19S**, **20S**) at low temperatures in *E. coli* strains. The soluble enzymes were purified by Ni‐IMAC followed by cleavage of the SUMO tag of **17S**, **18S**, **20S**, and **21S**; b) SDS‐PAGE of SUMO fusion proteins **17S**, **18S**, and **20S** (red.: SDS, DTT 90 °C, ox.: SDS 20 °C).

We started with the expression of the known His6‐tagged construct for B3GlcAT1^[^ ^39^ ^]^ **17H** (AA 76–335) lacking the stem region (Scheme 5). The overexpression was tested in several *E. coli* strains and gave mainly insoluble target protein. Active enzyme **17H** was isolated from the soluble fraction after purification via Ni‐IMAC. While LC‐MS analysis was consistent with the calculated molecular weight (30 987 Da) gel filtration of the purified enzyme indicated a mixture of oligomers (ca. 600 kDa) and a species with an apparent MW in the expected range (see Figure S23). In accordance with the report of Pedersen^[^ ^39^ ^]^ the truncated enzyme **17H** was prone to precipitate in buffers. Although this could be remedied by storing and reacting the enzyme in a modified buffer (20 mM MES, 1 M NaCl, pH 6.7), we tested a new construct (**17S**) where a His6SUMO^[^ ^40^ ^]^ domain was fused to slightly extended B3GlcAT1 (AA 72–335). This construct gave considerably more soluble protein, and the final yields of **17S** were up to five times higher in *E. coli* strains compared with **17H** (see Supporting Information). The SUMO fusion protein **17S** (42.5 kDa) eluted as a single peak during gel filtration and remained soluble at lower NaCl concentrations.

The beneficial effect of a SUMO domain fused to the *N*‐terminus of a glycosyltransferase^[^ ^41^ ^]^ was also tested with the human B4GalT7. This enzyme was reported to give inclusion bodies when expressed without a tag in *E.coli* but yielded soluble protein with galectin1 or MBP as a fusion tag.^[^ ^42^ ^]^ We designed a plasmid with a His6SUMO‐tag linked to the catalytically active B4GalT7 82–327 sequence (**18S**). Expression of the fusion protein in *E. coli* mainly gave insoluble protein even in the ArcticExpress strain. The soluble fraction was purified by Ni‐IMAC and provided 11 mg of **18S**/L of culture after gel filtration. After enzymatic cleavage of the His6SUMO domain with SENP2 the B4GalT7 82–327 **18** (28.7 kDa) gratifyingly remained soluble.^[^ ^42^ ^]^ Transferase activity was found for the fusion protein **18S** as well as for the cleaved form **18**. The protein **18** (28 699 Da) was further characterized by LC‐MS (deconvoluted mass of 28 679 Da) and CD spectroscopy.

Subsequently, the use of the SUMO‐tag was also tested for the overexpression of human B3GalT6 in *E. coli*. As there was no literature precedent for obtaining active B3GalT6 in *E. coli* we decided to fuse the SUMO‐tag to the entire lumenal region (36–329) since in some cases the flexible stem region assists in the folding of glycosyltransferases.^[^ ^43^ ^]^ Out of four *E. coli* strains tested only Origami2 and Shuffle T7 gave soluble fusion protein **19S** after Ni‐IMAC purification (1.5 mg of **19S** and 12 mg of **19S**/L of culture, respectively). The fusion protein **19S** was purified at pH 7.0 since at pH 8.0 precipitation started to occur (pI = 8.5). Remarkably, SDS‐PAGE of the unreduced fusion protein **19S** (46.6 kDa) showed a band at 130 kDa, whereas after reduction with DTT the band appeared the anticipated size of 50 kDa, which is analogy to the enzyme expressed in HEK cells by Huang.^[^ ^8^ ^]^ The enzymatic activity of **19S** was confirmed with the glycopeptide substrate **14** and it was found that the enzyme could be stored at 4 °C for two years without significant loss of activity. We initially kept proline 36 as the first amino acid of the transferase, which precluded removal of the directly attached SUMO tag using SENP2.^[^ ^44^ ^]^ A cleavable construct was obtained by replacing proline 36 with a serine (**20S**). The expression and the properties on SDS‐PAGE of the mutant fusion protein **20S** were equal to **19S**. Cleavage of the SUMO‐tag occurred readily and B3GalT6 **20** was isolated by gel filtration. Analysis of **20** (33 316 Da) by HPLC‐ESI‐MS showed a mass of 66 617 Da whereas after reduction the main mass was 33 313 Da. The CD spectrum of **20** was resembling that of B4GalT7 **18**. After optimized expression and purification, the yields of B3GalT6 **20** were reproducibly over 18 mg/L of culture.

### Side Reactions in Enzymatic Elongations

With the recombinant glycosyltransferases in hand, we studied the chemoenzymatic synthesis of the proteoglycan linkage region. The use of xylosyltransferase‐I and B4GalT7 in the enzymatic synthesis of bikunin glycopeptides is straightforward.^[^ ^3^, ^8^, ^10^ ^]^ However, depending on the reaction conditions and the substrates the biosynthetically following transferases B3GalT6 and B3GlcAT1 gave unexpected side products. When reacting the bikunin 1–25 glycopeptide **A** simultaneously with the three glycosyltransferases **18S**, **17S**, **19S** and their donor substrates, the reaction provided the expected glycopeptide **16** with the complete linkage region tetrasaccharide accompanied by traces of a truncated glycopeptide **22** lacking one of the galactoses (1%–2%) as detected by LC‐MS (Scheme 6).

Scheme 6Side reactions during a) enzymatic extension of xylosylated bikunin 1–25 **A** using a cocktail of B4GalT7 **18S**, B3GalT6 **19S** and GlcAT1 **17S** gave **16** and traces of truncated glycopeptide **22**; b) enzymatic extension of bikunin 1–50 *N*,*O*‐glycopeptide hydrazide **23** using a cocktail of **18S** and **19S** followed by **17S** mainly gave **24** accompanied by products with additional GlcA and Gal residues (**25**); c) enzymatic glucuronylation of *N*‐glycan azide **26** by GlcAT1 **17S**; d) multiple enzymatic galactosylation of *N*‐glycan azide **26** by B3GalT6 **19S**; e) multiple enzymatic galactosylation of *O*‐glycopeptide **A** using B4GalT7 **18S** and B3GalT6 **19S** followed by subsequent glucuronylation with GlcAT1 **17S**; preparative synthesis f) of **22**; g) of **16**. h) Synthesis of bikunin 1–50 *N*,*O*‐glycopeptide hydrazide **24** via native chemical ligation.

**Figure:**
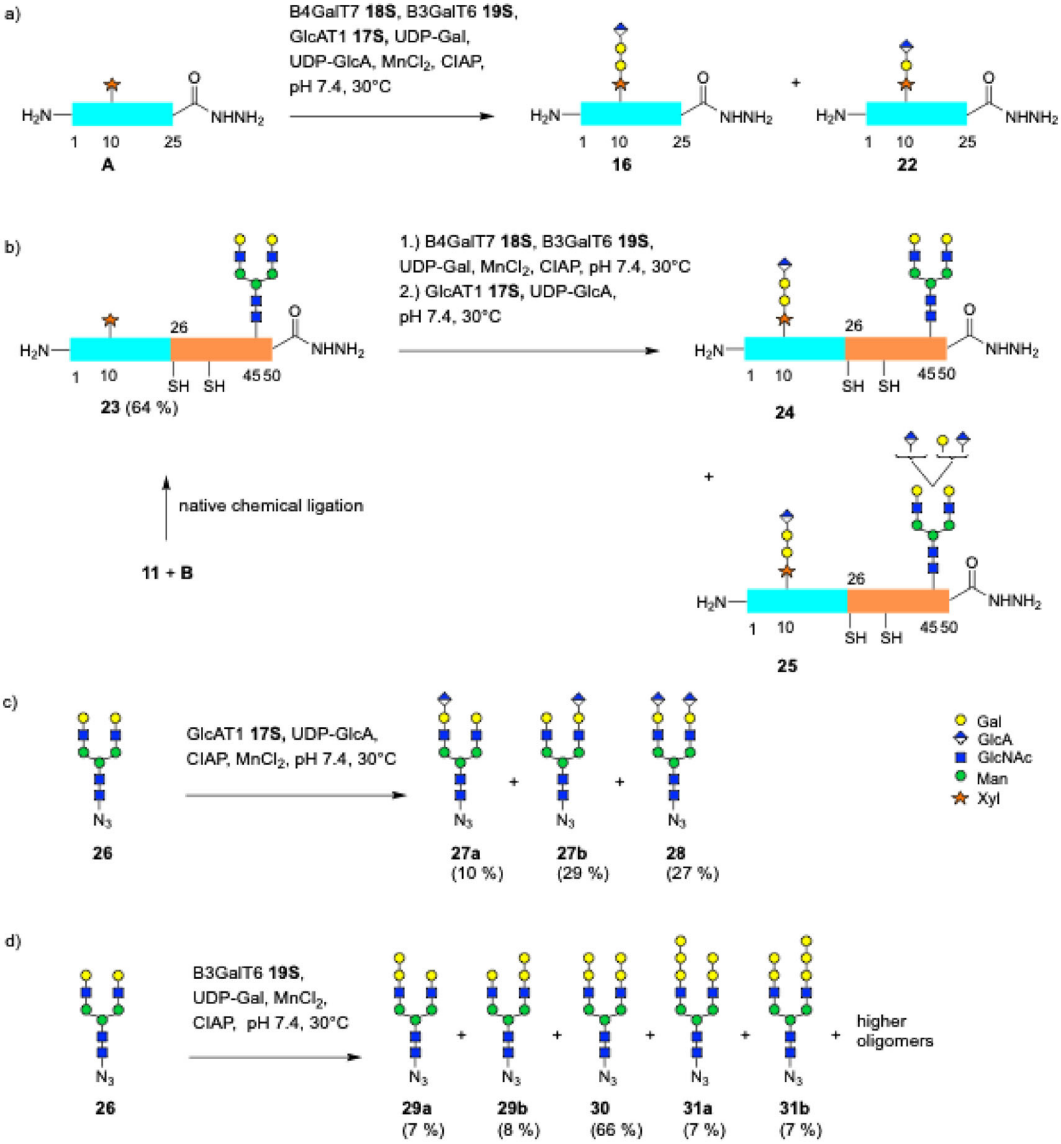

**Figure:**
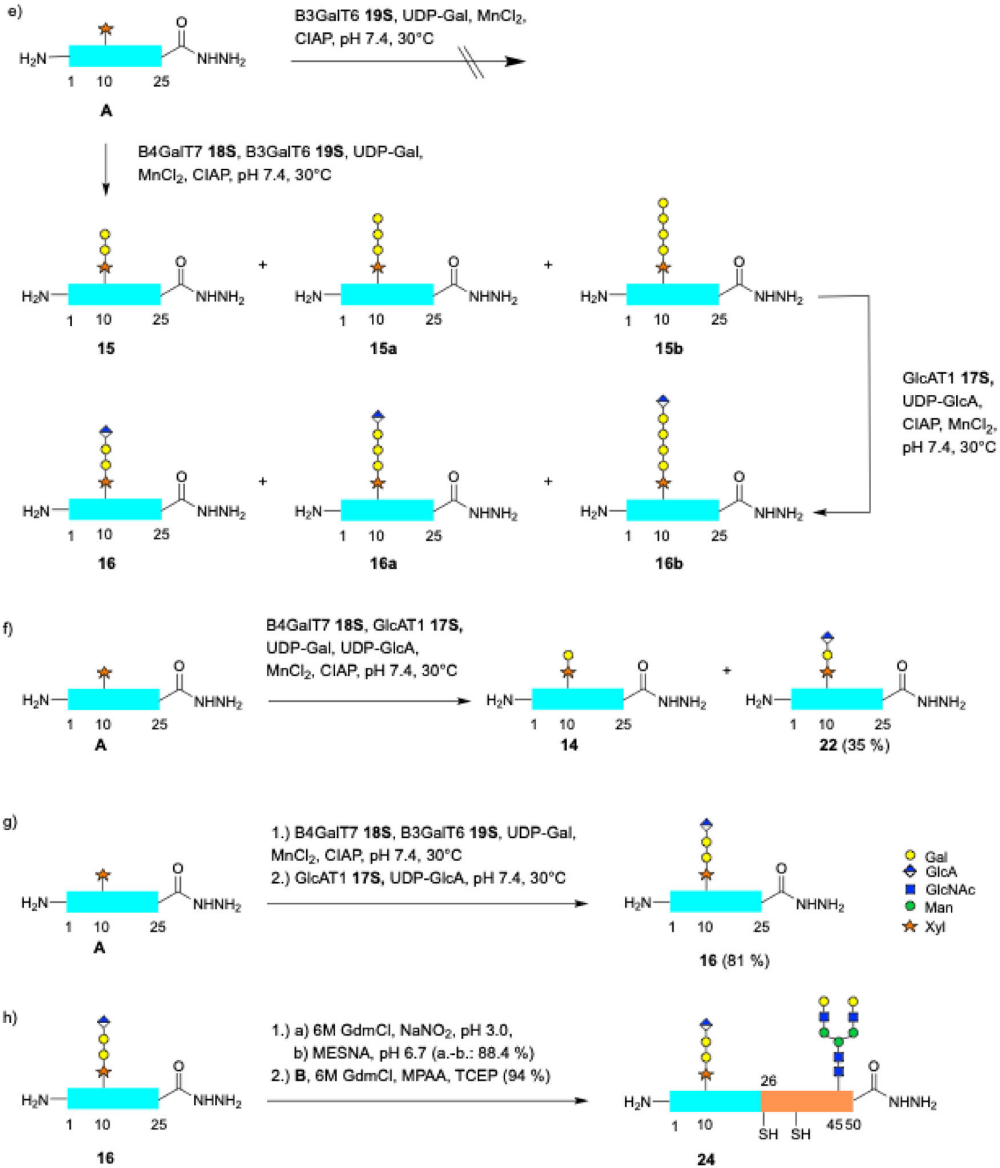

In a subsequent experiment, the *N*,*O*‐glycosylated peptide 1–50 **23** (obtained by ligation of the 1–25 thioester **11** and **B**) was first reacted with the two galactosyltransferases **18S** and **19S** to ensure complete galactosylation followed by glucuronylation with **17S**. Besides the anticipated main product **24** with the complete tetrasaccharide a series of side products (**25**) was found with higher masses corresponding to additional Gal and GlcA residues. We assumed that the unexpected transfer of GlcA and Gal moieties occurred at the *N*‐glycan and thus investigated the substrate specificity of human B3GlcAT‐I in more detail.

Indeed, when reacting **17S** with the *N*‐glycan azide **26** (5 mM) and UDP‐GlcA for 3d the LC‐MS analysis showed the formation of two monoglucuronylated *N*‐glycans **27a**, **b** accompanied by the glycan with two glucuronyl residues (**28**). The unsymmetric *N*‐glycans were separated and unexpectedly displayed preferred transfer onto the 1,6‐arm (**27b**) as shown by HR‐MS‐MS using characteristic cross ring fragmentations of anionic parent ions.^[^ ^45^ ^]^ Thus, human GlcAT‐I can also convert β1,4‐linked terminal galactosides, albeit at a lower rate compared to the β1,3‐linked galactose in the natural acceptor trisaccharide. Previous publications on hGlcAT‐I was reported that free *N*‐acetyllactosamine was not an acceptor^[^ ^46^, ^47^ ^]^ and that the non‐natural naphthylmethyl glycoside of *N*‐acetyllactosamine was reacting poorly.^[^ ^48^ ^]^ Glucuronylation of *N*‐glycans was so far only described for the related glucuronyl transferases hGlcAT‐S^[^ ^49^ ^]^ and hGlcAT‐P,^[^ ^46^, ^47^, ^48^ ^]^ which can also contribute to GAG linkage region biosynthesis.

Additional specificity experiments for B4GalT7 and B3GalT6 were carried out. No transfer of galactose was found when xylosyl‐glycopeptide **A** was reacted with B3GalT6 **19S** and UDP‐Gal. However, when *N*‐glycan **26** was reacted with B3GalT6 **19S** for 1d LC‐MS showed that most of the *N*‐glycan was consumed and products with 1–3 additional galactose units had formed. Using stringent conditions (concentrated enzyme and UDP‐Gal) the number of transferred Gal residues could be increased to 6–13 (data not shown). In a preparative reaction (8 mM **26**, 24 mM UDP‐Gal) five compounds were obtained after separation on a graphite column. The main product **30** had an additional galactose in each antenna. Furthermore, unsymmetric *N*‐glycans with one (**29a**, **b**) or three additional galactoses (**31a**, **b**) were obtained. We assigned the regioisomers by HR‐MS‐MS as above^[^ ^45^ ^]^ and ^1^H‐NMR spectroscopy (see Supporting Information).

Subsequently, we investigated if B3GalT6 (**19S**) and hGlcAT‐I (**17S**) can also lead to side products on the O‐linked Gal‐Gal‐Xyl trisaccharide in glycopeptide **15**. This was tested by galactosylating glycopeptide **A** (6 mM) with the galactosyltransferases **18S** and **19S** and 8 + 8 equiv. of UDP‐Gal. After 24 h the LC‐MS analysis showed **15** as the main product accompanied by larger species (**15a**, **b**) with additional Gal residues in a ratio of 100:42:3 based on MS intensity, respectively. The subsequent reaction with GlcAT‐I **17S** and UDP‐GlcA (28 mM) for 3 h provided the canonical tetrasaccharide (**16**) and novel glucuronylated products with extended Gal chains (**16a**, **b**), albeit with incomplete conversion of the non‐natural substrates (ca. 40%).

The observed promiscuities of hGlcAT‐I suggested that the initially detected deletion product **22** should result from an irregular transfer of glucuronic acid onto the Gal‐β1,4‐Xyl disaccharide of glycopeptide **14** omitting B3GalT6. This was tested by galactosylating the 1–25 peptide **A** (6 mM) with B4GalT7 **18S**/UDP‐Gal and repeated additions GlcAT‐I **17S**/UDP‐GlcA converting most of **14** to the glucuronylated product **22** (ca. 85% conversion) revealing the latent reactivity of GlcAT‐I towards Gal‐β1,4‐Xyl as an alternative acceptor. The non‐canonical glycopeptide **22** was purified by RP‐HPLC (35% yield) and characterized by HR‐MS and NMR spectroscopy. Thus, hGlcAT‐I can bypass the canonical biosynthetic pathway to proteoglycans by a slow glucuronylation of the shortened acceptor (Gal‐Xyl) lacking the second galactose unit. This correlates well with the small amounts (0.1%) of the truncated linkage region trisaccharide detectable in bikunin of healthy individuals.^[^ ^50^ ^]^ The promiscuity of GlcAT‐I thus explains the unexpected formation of the non‐canonical truncated linkage region glycopeptide **22** and provides a salvage path allowing GAG biosynthesis in B3GalT6 knock out cells.^[^ ^51^ ^]^ In patients affected by pathogenic (non‐functional) variants of the B3GalT6 gene initiation of GAG biosynthesis is severely impaired and weakens the connective tissue (spondylodysplastic Ehlers‐Danlos‐syndrome). These patients show highly increased levels of the truncated linkage region trisaccharide.^[^ ^52^ ^]^ A study of double knockouts of B3GalT6 in zebrafish^[^ ^53^ ^]^ gave similar results. This data is fully in accordance with our findings.

The assembly of bikunin 1–25 with the canonic linkage region tetrasaccharide (**16**) was readily carried out on preparative scale in a stepwise manner. Bikunin 1–25 hydrazide **A** (5 mM) and 3 equiv. of UDP‐Gal were incubated simultaneously with the two galactosyltransferases **18S** and **20S**. After 16 h the glucuronyltransferase **17S** and UDP‐GlcA were added. Nearly complete conversion for each elongation was achieved after 24 h of total reaction time resulting in an isolated yield of 81% of **16** after RP‐HPLC. Thus, a high yielding and robust chemoenzymatic synthesis for the key building block **16** was developed. The designated anchoring point for further extension to a chondroitin chain should facilitate the chemoenzymatic assembly of *N*,*O*‐glycosylated glycoforms of bikunin. Using native chemical ligation the linkage region glycopeptide hydrazide **16** was coupled with *N*‐glycopeptide **B** furnishing the bikunin 1–50 *N*,*O*‐glycopeptide **24** devoid of side products at the *N*‐glycan.

In human plasma *N*‐glycans with terminal glucuronic acid were detected, albeit at a very low rate.^[^ ^54^ ^]^ Their low abundance can be rationalized by the different Golgi‐localization of the enzymes involved. GAG linkage region biosynthesis^[^ ^55^ ^]^ was shown to occur in the early‐Golgi whereas galactosylation^[^ ^56^ ^]^ and sialylation of *N*‐glycans is located in the late‐Golgi. Thus cross‐reactivity is unlikely to occur. Furthermore, the reactivity of B3GlcAT‐I and B3GalT6 toward galactosylated *N*‐glycans is low compared to sialyltransferases.

### X‐Ray Crystallography of B3GalT6

Since the structure of B3GalT6 had not been determined we screened for suitable crystallization conditions. The solubility‐enhancing effect of the SUMO‐domain permitted highly concentrated solutions (20 mg **19S**/mL). Despite the addition of MnCl_2_ and UDP‐Gal no crystals of **19S** were obtained. We thus examined the untagged transferase **20**. However, protein **20** precipitated at concentrations above 2 mg/mL. When adding 20% glycerol **20** remained soluble up to 6 mg mL^−1^. This stock solution was used for crystallization screens. In about 30% of the wells diffuse crystallization occurred within 2 d. In two wells single crystals formed within 1 d reaching maximum size after 6 d. Using an in‐house X‐ray source showed that these protein crystals diffracted to a resolution of 1.2 Å and belonged to space group C222(1). Since molecular replacement was not successful the crystals were treated either with thiomersal or HgCl_2_ each at 1 mM for overnight soaking. With these heavy atom derivatives, the phase problem could be solved and the crystal structure of human B3GalT6 was obtained (PDB accession code 9SP7).

**Scheme 7:**
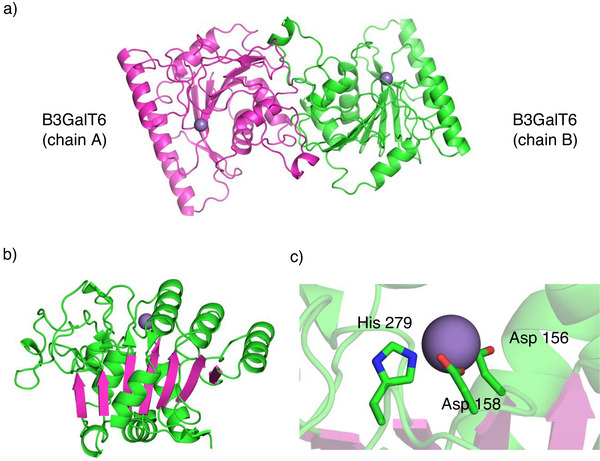
a) Crystallographic B3GalT6 dimer **20** formed by the given crystallographic dyad (PDB ID: 9SP7). The protein chains A and B are highlighted in magenta and green, Mn^2+^ in deep purple. b) Rossman fold (GT‐A) of B3GalT6 (only one chain displayed). The seven central β‐sheets are highlighted in magenta. c) Tripodal complexation of the catalytic Mn^2+^ (purple) in the active site of the enzyme. The figures were created by using the PyMOL Molecular Graphics System, Version 2.4.2 Schrödinger, LLC.

The glycosyltransferase shows a GT‐A fold with a the single Rossman domain displaying the typical topology of the β‐sheets (Scheme 6b).^[^ ^57^, ^58^ ^]^ Furthermore, the crystal packing revealed a close contact to a symmetry related monomer leading to a dimeric B3GalT6 structure (Scheme 7a). The occupancy of the Mn^2+^ binding sites in the crystal was not complete (∼ 50%) and the UDP‐Gal added prior to crystallization was not detectable, thus, the crystals correspond to an apo‐form. As expected, binding of Mn^2+^ occurred at the D156XD158 motif. In the crystal structure Mn^2+^ is complexed in a tripodal mode by two Asp carboxylates (D156, D158) and the ΝΕ2‐nitrogen of His279 as an additional ligand (Scheme 6c). This arrangement is similar to the binding of Mn^2+^ in rabbit muscle glycogenin (pdb:1LL2; D101, D103, H211)^[^ ^59^ ^]^ a glycosyltransferase also forming a dimer in solution.^[^ ^60^ ^]^ From the predicted stem region (35–56)^[^ ^61^ ^]^ only a short stretch (54–56) was resolved.

**Scheme 8:**
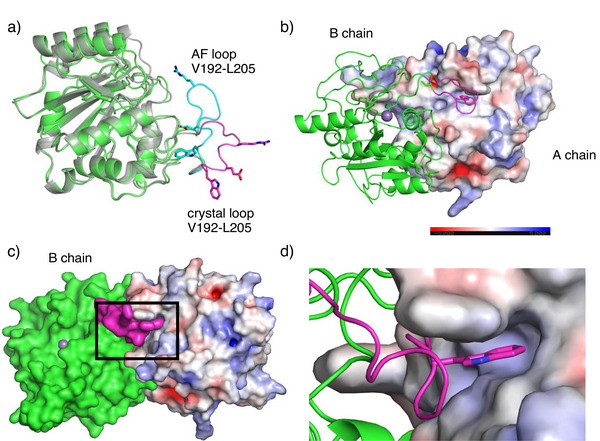
a) Ribbon type overlay of the crystal structure of B3GalT6 **20** in green and the Alphafold model (AF‐Q96L58‐F1) in gray. The loop Val192‐Leu205 is highlighted in magenta (crystal structure) and blue (Alphafold monomer). b) Electrostatic surface representation of the dimerization interface of B3GalT6 displayed on chain A, the chain B is highlighted in green and loop Val192‐Leu205 is highlighted in magenta. c) Interaction of the Val192‐Leu205 loop of chain B (magenta) with a complementary cleft in chain A of dimeric B3GalT6. d) Close up of the deep cleft interacting with the Val192‐Leu205 loop of chain B showing the binding mode of the Trp203 side chain.

An overlay of one monomer of the experimental crystal structure with the theoretical hB3GalT6 monomeric model calculated by Alphafold^[^ ^62^, ^63^ ^]^ (AF‐Q96L58‐F1) showed a high degree of identity of the basic GT‐A fold. Remarkably, the crystal structure deviates strongly from the theoretical model in the loop Val192‐Leu205, which is part of the dimerization surface (see Scheme 8ab). The contact area of the dimer was calculated with PISA^[^ ^64^ ^]^ and resulted in a buried surface of 1256 Å^2^ and a total surface of 13 897 Å^2^ per monomer. The dimerization surface is predominantly hydrophobic and displays knobs and clefts, which perfectly interlock (see Scheme 8b–d). Among those complementary topologies the Trp203 side chain is unique, acting as a flat pin filling a deep cavity located at the open end of the fissure covered by the Val192‐Leu205 loop.

**Scheme 9:**
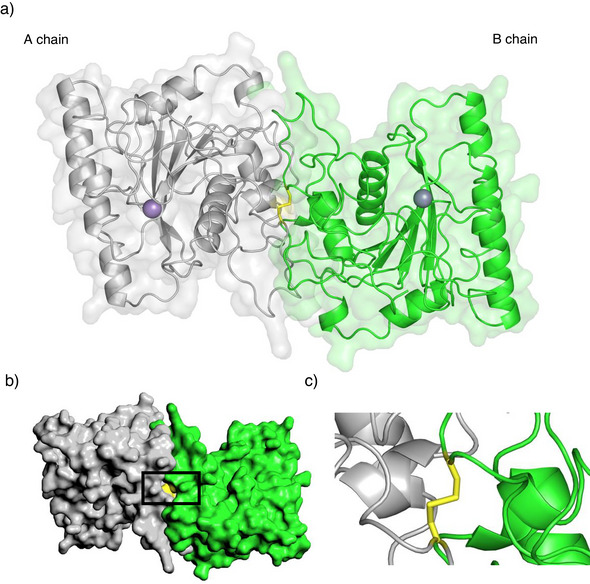
a) Crystal structure of dimeric B3GalT6 with the intermolecular disulfide Cys206(A)‐Cys206(B) highlighted in yellow. b) Surface accessibility of the intermolecular disulfide Cys206(A)‐Cys206(B) highlighted in dimeric B3GalT6. c) Close up of the intermolecular disulfide Cys206(A)‐Cys206(B).

Within the catalytic region B3GalT6 contains five cysteines. Intramolecular disulfides bridges were found at Cys271‐Cys300 and a rare vicinal disulfide Cys321‐Cys322 with the backbone in trans configuration.^[^ ^65^, ^66^ ^]^ The most surprising feature of the dimeric B3GalT6 structure, however, was the formation of a covalent dimer via a disulfide bridge of Cys206 of both monomers (Scheme 9). The distance between the two sulfur atoms is 2.1 Å and a dihedral angle of 97° representing typical values for disulfide bonds. The interchain disulfide is not entirely buried in the dimerization interface but appears accessible from the surface via a small opening.

To investigate the role of Cys206 in the formation of dimeric B3GalT6 we mutated Cys206 to Ser206 (see SI). The mutant transferase **21S** (Ser206) expressed well and was enzymatically active, however, its activity was reduced to about 60% compared with the disulfide‐linked enzyme **20S**. Gel filtration showed that the Ser 206 mutants **21S** and the deSUMOylated transferase **21** also eluted as a dimer (vide infra). Analysis of **21** by SDS‐PAGE (33 kDa) and LC‐MS (33 322 Da) gave the anticipated mass of the monomeric protein. Thus, the dimerization of B3GalT6 in solution is not dependent on a free thiol at Cys206. We thus speculate that in the native enzyme the monomers initially associate via the large dimerization interface and are subsequently covalently locked by the intermolecular Cys206 disulfide.

The potential conservation of dimeric B3GalT6 in animals was investigated by alignment of 19 mammalian, avian, fish, amphibian, nematode and insect B3GalT6 protein sequences (see Figure S52). The high degree of identical residues revealed several conserved motifs including the DXD loop for nucleotide binding and the two intramolecular disulfides. A major part of the conserved residues (48 total) is located in the active site and in the dimerization domain surrounding Cys206 (see Supporting Information). Within the set of investigated sequences only in two *Drosophila* species the residue corresponding to Cys206 was a serine, which does not interfere with dimer formation according to the experimental evidence shown above. Thus, for higher animals B3GalT6 should be a conserved disulfide‐linked dimer.

Biochemical evidence for homodimeric or heteromeric complexes of eukaryotic glycosyltransferases has been reported in many cases^[^ ^67^ ^]^ whereas covalent homodimers via disulfides are limited to a few examples with only little structural data.^[^ ^68^ ^]^ Interchain disulfides were found in the cytoplasmic or transmembrane domain, the stem region and in a few cases^[^ ^69^, ^70^ ^]^ also in the catalytic domain. Disulfide formation within a large dimerization interface of the catalytic domain has to the best of our knowledge not been reported before. Human B3GalT6 appears to be the first structurally characterized example of a covalently linked dimeric glycosyltransferase.

We have then analyzed the molecular weights of the three consecutively acting glycosyltransferases B4GalT7, B3GalT6 and B3GlcAT‐I by SEC‐MALS. All the SUMO‐linked transferases (constructs **17S**, **18S**, **20S**, **21S**) eluted as dimers although only **20S** was covalently linked. Their apparent molecular weights were calculated from a SEC chromatogram using protein standards (see Supporting Information). For the SUMO fusions (**17S**, **18S**, **20S**, **21S**) the MALS data corresponded to the theoretical values of dimers. However, their apparent molecular weights (SEC) were higher than anticipated (+15%–37%), presumably due to the SUMO domain. Cleavage of the SUMO domain in the dimers occurred stepwise via a heterodimeric species with one SUMO tag remaining, which could be resolved by SEC in the case of **20S** and **21S**. The fully deSUMOylated transferases **17**, **18**, **20**, **21** remained dimeric in solution as evidenced by SEC‐MALS. The dimeric state of enzyme **18** in solution is in accordance with the dimer apparent in the crystal structure.^[^ ^38^ ^]^ The dimeric nature of B4GalT7, B3GalT6 and B3GlcAT‐I in solution was recently described in a publication by the Wild group.^[^ ^9^ ^]^

Thermal unfolding of the SUMOylated B4GalT7 **18S,** B3GalT6 **20S** and **21S** was tested by CD‐spectroscopy and revealed melting points of 55, 59 and 53 °C, respectively (Supporting Information). Thus, the lack of the intermolecular disulfide in the Ser206 enzyme **21S** (53 °C) lowers thermal stability relative to the disulfide linked enzyme **20S** (59 °C). Surprisingly, under these conditions B3GlcAT‐I **17S** appeared to remain stable. The data for **18S** and **20S** correspond well to the values for other constructs determined independently using DSF.^[^ ^9^ ^]^

The dimerization interfaces of the linkage region transferases showed that mainly hydrophobic interactions are responsible for dimerization. This presumably renders their dimeric status independent of pH and other factors. The occurrence of dimeric glycosyltransferases in the Golgi apparatus has been proposed for some time (kin recognition model)^[^ ^71^, ^72^ ^]^ and recently the number of crystal structures showing glycosyltransferase dimers^[^ ^73^ ^]^ forming dimers in solution,^[^ ^74^, ^75^ ^]^ is steadily increasing. Even the activity of complex bifunctional glycosyltransferases involved in polysaccharide synthesis is related to the formation of homo‐ and heterodimers exemplified by the structures of bifunctional LARGE1^[^ ^76^ ^]^ (matriglycan) and the heparan sulfate polymerase complex EXT1‐EXT2.^[^ ^77^ ^]^ A detailed analysis of the Golgi apparatus of *Chlamydomonas* by cryo‐ET revealed a densely populated interior protein array with repetitive globular structures^[^ ^78^ ^]^ in lateral repeats of 6x12 nm. This correlates well with the size of dimeric glycosyltransferases anchored in the membrane and lends further evidence to a condensed type of the kin recognition model in the Golgi.

## Conclusion

The synthetic efforts to access defined forms of the proteoglycan bikunin using a chemoenzymatic approach revealed several unexpected findings. We found that *N*‐terminal SUMO‐fusions of the human linkage region glycosyltransferases B4GalT7, B3GalT6 and B3GlcAT‐1 generally gave high yields of soluble and active enzymes in *E. coli* strains. The transferases performed well in enzymatic cascade reactions and provided the desired linkage region tetrasaccharide when starting with xylosylated bikunin glycopeptides even without phosphorylation of the xylosyl residue by the kinase FAM20B.^[^ ^8^, ^9^, ^10^, ^11^, ^79^ ^]^ B3GalT6 and B3GlcAT‐1 led to side products with *N,O*‐glycosylated bikunin peptides revealing unexpected promiscuity of both enzymes towards complex type *N*‐glycans. Additionally, B3GalT6 was found to synthesize poly‐β3 Gal structures. B3GlcAT‐1 can slowly convert the biosynthetic intermediate Gal‐Xyl to the non‐canonical trisaccharide GlcA‐Gal‐Xyl. This reaction was carried out on a preparative scale and independently confirmed the biosynthetic bypass to GAGs in the case of dysfunctional B3GalT6 (spondylodysplastic Ehlers‐Danlos‐syndrome). The three linkage region glycosyltransferases B4GalT7, B3GalT6 and B3GlcAT‐1 were dimeric in solution and the crystal structure of B3GalT6 was solved showing a covalent dimer linked by a disulfide in the center of the dimerization domain. Part of this interface is a 13AA loop providing a tightly fitting clamp, which was not predicted in this orientation by the monomeric Alphafold model. The dimeric B3GalT6 motif appears to be conserved in higher organisms and reinforces the concept of dimeric glycosyltransferases lining the Golgi.

## Conflict of Interests

The authors declare no conflict of interest.

## Supporting information



Supporting Information

## Data Availability

The data that support the findings of this study are available in the supplementary material of this article. Diffraction data and refined crystal structure of B3GalT6 have been deposited with the Protein Databank (https://www.rcsb.org/) under accession code 9SP7.
