# Supplementary material for: Human Proteoglycan Linkage Region Glycosyltransferases are Dimeric and Show Unexpected Specificities
Source: Angew Chem Int Ed Engl. 2025 Nov 29;65(3):e16855. doi: 10.1002/anie.202516855 (PMC12811666; doi:10.1002/anie.202516855)
Supplement: Supplementary file 1 — Supporting Information [file ANIE-65-e16855-s001.pdf]

1. General Methods

2. General Synthesis Procedures

3. Synthesis of Bikunin 1-25 Glycopeptides
3.1 Synthesis of Xylosyl-Serine Building Blocks **3** and **9**
3.2 Synthesis of Bikunin 11-25 Resin **2**
3.3 Synthesis of Bikunin 1-9 Peptide **6**
3.4 Convergent Synthesis of Bikunin 1-25 Glycopeptide **7**
3.5 Stepwise Synthesis of Bikunin 1-25 Glycopeptide **7**
3.6 Synthesis of Bikunin 1-25 Glycopeptide Thioester **11** and Hydrazide **A**

4. Synthesis of Bikunin 26-50 Glycopeptide Hydrazide B
4.1 Synthesis of Bikunin 26-50 Hydrazide **12**
4.2 Synthesis of Bikunin 26-50 Glycopeptide Hydrazide **B**

5. Enzymatic Elongation of Bikunin 1-25 Glycopeptides
5.1 Synthesis of Bikunin 1-25 (Gal-Gal-Xyl)-Glycopeptide **15**
5.2 Synthesis of Bikunin 1-25 (GlcA-Gal-Gal-Xyl)-Glycopeptide **16**

6. Recombinant Expression of Linkage Region Glycosyltransferases
6.1 Expression and purification of B3GlcAT-I **17**
6.2 Expression and purification of SUMO-B3GlcAT-I **17S**
6.3 Expression and purification of SUMO-B4GalT7 **18S**
6.4 Expression and purification of SUMO-B3GalT6 **19S, 20S, 21S**
6.5 Cleavage of the SUMO tag from **17S**, **18S**, **20S**, **21S**
6.6 SEC and SEC-MALS of **17S, 18S, 20S, 21S** and **17, 18, 20, 21**

7. Enzymatic Glycosylation of Glycopeptides and N-Glycans
7.1 Enzymatic Glycosylation of Bikunin 1-25 Glycopeptide Hydrazide **A**
7.2 Synthesis of Bikunin 1-50 Glycopeptide Hydrazide **23**
7.3 Enzymatic Glycosylation of Bikunin 1-50 Glycopeptide Hydrazide **23**
7.4 Enzymatic Glucuronylation of N-Glycan Azide **26**
7.5 Enzymatic Galactosylation of N-Glycan Azide **26**
7.6 Enzymatic Synthesis of Bikunin 1-25 Glycopeptides with extended linkage region
7.7 Preparative Synthesis of Bikunin 1-25 Glycopeptide **22** with truncated linkage region
7.8 Preparative Synthesis of Bikunin 1-25 linkage region Glycopeptide **16**
7.9 Synthesis of Bikunin 1-50 Glycopeptide Hydrazide **24**

8. Crystallization of B3GalT6 18
8.1 Overexpression and Crystallization of B3GalT6 **18**
8.2 Relative activity of **20S** and **21S**
8.3 Thermal unfolding of **18S,** B3GalT6 **20S** and **21S**, **17S**
8.4 Sequence alignment of B3GalT6 from various animal species

9. NMR spectra

10. References

1. General Methods

Solvents were dried according to standard methods. Automated Fmoc-SPPS was performed in 45-mL reaction vessels in a PTI Tribute peptide synthesizer with UV-monitoring and feedback control system. Manual SPPS steps were performed in 2-mL, 5-mL, 10-mL or 20-mL polypropylene syringes equipped with a 25 µm polyethylene filter (Multisyntech, Germany). Fmoc or Boc protected amino acids and coupling reagents were obtained from Novabiochem (Läufelfingen, Switzerland), Iris Biotech (Marktredwitz, Germany) and Sigma Aldrich (Taufkirchen, Germany). 2Cl-Trityl-ChemMatrix resin was synthesized according to published procedures.[1] Trityl-ChemMatrix resin was obtained from PCAS BioMatrix Inc., Canada). 2Cl-Tritylchloride-polystyrene resin resin was obtained from Iris Biotech (Marktredwitz, Germany). Microwave assisted reactions were performed using a µCHEMIST instrument from MWS GmbH (Leutkirch, Germany).

ESI-TOF mass spectra were recorded on a Micromass LCT or LCT Premier instrument coupled to an Agilent 1100 HPLC or a Waters ACQUITY UPLC H-Class System with a photodiode array detector using solvent A (H_2O + 0.1 % HCOOH) and B (MeCN + 0.1 % HCOOH). HR-ESI mass spectra were recorded on a Thermo Q Exactive Orbitrap mass spectrometer.

Preparative size exclusion chromatography was performed on an Äkta Purifier system with conductivity detection and UV-detection at 214, 254 and 280 nm.

Flash chromatography was performed manually or on a GRACE Reveleris® iES-flash chromatography system with ELS-detector. Thin layer chromatography was performed on coated aluminum plates (silica gel 60 GF_{254}, Merck Darmstadt). Spots were detected by UV light or by charring with a 1:1 mixture of 2 N H_2SO_4/0.2 % resorcinol monomethyl ether in ethanol. Dialysis tubing Zellutrans V Serie (MW cutoff: 5000, flat width 40 mm) was obtained from Roth (Germany).

CD spectra were recorded on a Jasco J-715 spectropolarimeter. A 1 mm cuvette (110-QS, Hellma Analytics, fused silica Suprasil) was used. The ellipticities ΔΘ were corrected by a matrix blank and were normalized as residual molar ellipticities $[\Theta]_{MRW}$: $[\Theta]_{MRW}$ (deg cm^2 dmol^{-1}) = ΔΘ (mdeg) / (l (mm) × [**EPO A**] (mol/L) × (N_{aa} - 1)). l is the layer thickness of the cuvette and N_{aa} is the number of amino acid residues in the protein.

UDP-Gal was kindly provided by Roche Diagnostics GmbH. Alkaline phosphatase from calf intestine was purchased from Sigma Aldrich (10.3 mg/mL with 2145 U/mg, Buffer: 30 mM triethanolamine, pH 7.6, 3 M NaCl, 1 mM $MgCl_2$, 0.1 mM $ZnCl_2$).

The degree of loading of resins was determined by Fmoc cleavage of the penultimate amino acid.[2,3] Absorbance measurements were performed on a Specord 2000 spectrophotometer from Analytik Jena. Analytical TFA deprotections were performed by adding 0.5 mg of resin or 0.2 mg of protected peptide to 100 µL of TFA/TIS/H_2O (95:2.5:2.5) for 60 min. The mixture was dried in high vacuum,

the residue was dissolved in MeCN/H_2O + 0.1 % HCOOH and analyzed by HPLC-MS.

2. General Synthesis Procedures

Automated Fmoc-SPPS

Solid-phase synthesis following the Fmoc strategy[3] was performed automatically in 45 mL reaction vessels on a Tribute peptide synthesizer with IntelliSynth UV Monitoring and Feedback Control System from Protein Technologies Inc. All reactions were performed at ambient temperature under a nitrogen atmosphere and mechanical shaking. Nitrogen was passed through the reaction suspension for additional mixing. Before starting the synthesis, the resin was swollen by washing with CH_2Cl_2 (5 x 30 s) and DMF (5 x 30 s).

Automated Removal of N^{α}-Fmoc

To remove *N*-terminal Fmoc protection a piperidine solution (20 % in DMF) was added to the resin and shaken for 0.5 min. The coupled UV monitoring system of the peptide synthesizer initiated further Fmoc cleavage cycles if the absorbance (A_{301}) of the filtrate indicated incomplete cleavage. After complete Fmoc removal, the resin was washed with DMF (5 x 30 s).

Automated Coupling of Fmoc amino acid derivatives

The amino acid building block and the activation reagent were taken up in a defined volume of DIPEA in DMF (for molarity see specific synthesis) before coupling, mixed for 2 min, and added to the deprotected peptidyl resin. Cysteine derivatives were activated as symmetric anhydrides and coupled manually. At the end of each coupling the resin was washed with DMF (5 x 30 s). The exact coupling conditions are given in the specific synthesis protocols.

Manual coupling of cysteine derivatives[4]

In dried glassware under argon, the cysteine building block (10 equiv.) was dissolved in dry DMF/CH_2Cl_2 1:2.5 (*c* Fmoc/Boc-Cys(PG)-OH = 180 mM) and *N*,*N*'-diisopropylcarbodiimide (5 equiv.) was added at 0 °C. The reaction mixture was stirred at 0 °C for 5 min and for 30 min at ambient temperature. Subsequently, the CH_2Cl_2 was removed under vacuum. The suspension was drawn up into a syringe with and added to the deprotected peptidyl resin swollen in DMF. The residue and the vessel were washed three times with an appropriate volume of DMF to give a concentration of 105 mM for the anhydride in the coupling solution. After 3 - 5 h of shaking at ambient temperature, the coupling solution was removed and the resin was washed with CH_2Cl_2 and DMF (five times each).

3. Synthesis of Bikunin 1-25 Glycopeptides

3.1 Synthesis of Xylosyl-Serine Building Blocks 3 and 9

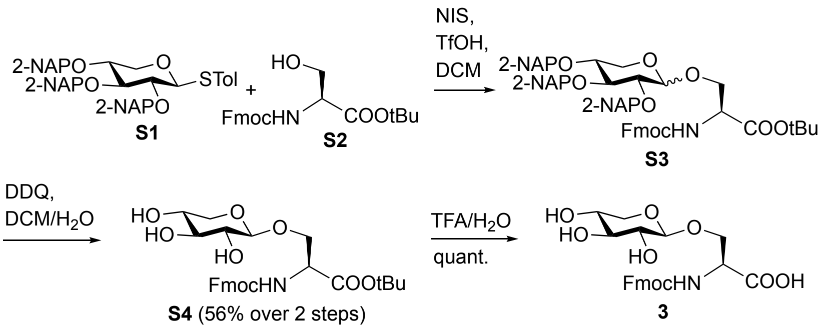

Figure S1: Synthesis of Fmoc-Ser(Xyl)-OH **2.**

A mixture of the D-xylose-derived thioglycoside **S1** (9.60 g, 14.2 mmol, synthesized in analogy to Ref.[5]), Fmoc-Ser-OtBu **S2**[6] (6.41 g, 17.0 mmol), and freshly dried 3 Å molecular sieves (25 g) in dichloromethane/acetonitrile (1/1, 400 mL) was stirred at room temperature for 1 h under ni-trogen atmosphere. The mixture was cooled to –30 °C for 15 min, *N*-iodosuccinimide (3.9 g, 17.0 mmol) and triflic acid (250 L, 2.84 mmol) were sequentially added to the reaction flask, and the mixture was stirred at –30 °C for 3 h. Triethylamine (1 mL) was subsequently added to quench the reaction, the suspension was filtered through celite, and the flask and the celite were rinsed with ethyl acetate. The filtrate was washed with 10% sodium thiosulfate and brine, dried over magnesium sulfate, filtered and concentrated under vacuum. The residue was purified by flash chromatography (ethyl acetate/hexane = 1/3) to give a mixture of anomers **S3** (11.4 g, α/β= 1/2), which were not separated.

The mixture **S3** (11.4 g, 12.1 mmol) was dissolved in dichloromethane/water (20/1, 228 mL) and 2,3-dichloro-5,6-dicyano-1,4-benzoquinone (16.6 g, 72.7 mmol) was added in three equal portions in 30-min intervals at room temperature. After stirring for 4 h, the reaction was quenched with 10% sodium thiosulfate (200 mL) and the aqueous layer was extracted with dichloromethane (3 x 200 mL). The combined organic layers were extracted with saturated sodium bicarbonate (1x 300mL) and water (1x 300mL), dried over magnesium sulfate, filtered and concentrated under vacuum. The residue was purified by flash column chromatography (ethyl acetate/hexane = 3/1, then methanol/chloroform = 1/15) to provide the desired β-triol **S4** (4.05 g, 56% over 2 steps).

The β-triol **S4** (4.05 g, 7.86 mmol) was dissolved in trifluoroacetic acid/water (95/5, 180 mL) at room temperature. After stirring for 2 h, toluene (250 mL) was added and the solvents were removed under vacuum to furnish Fmoc-Ser(Xyl)-OH **3** (3.61 g, quantitative yield). ^{1}H NMR (600 MHz, CD_3OD): δ 7.67 (d, J = 7.4 Hz, 2H), 7.57-7.55 (m, 2H), 7.28–7.26 (m, 2H), 7.21-7.19 (m, 2H), 4.33 (t, J = 3.6 Hz, 1H), 4.26-4.18 (m, 3H), 3.75 (dd, J = 5.4, 11.5 Hz, 1H), 3.63 (dd, J = 3.3, 10.0 Hz, 1H), 3.23-3.21 (m, 1H), 3.12–3.07 (m, 2H); ^{13}C NMR (150 MHz, CD_3OD): δ 173.8 (C), 159.0 (C), 145.6 (C), 145.4 (C), 142.8 (C), 129.1 (CH), 128.5 (CH), 126.6 (CH), 126.5 (CH), 121.2 (CH), 105.5 (CH), 77.9 (CH), 75.1 (CH), 71.4 (CH), 70.9 (CH), 68.4 (CH), 67.2 (CH), 55.9 (CH), 50.2 (C), 48.6 (CH).

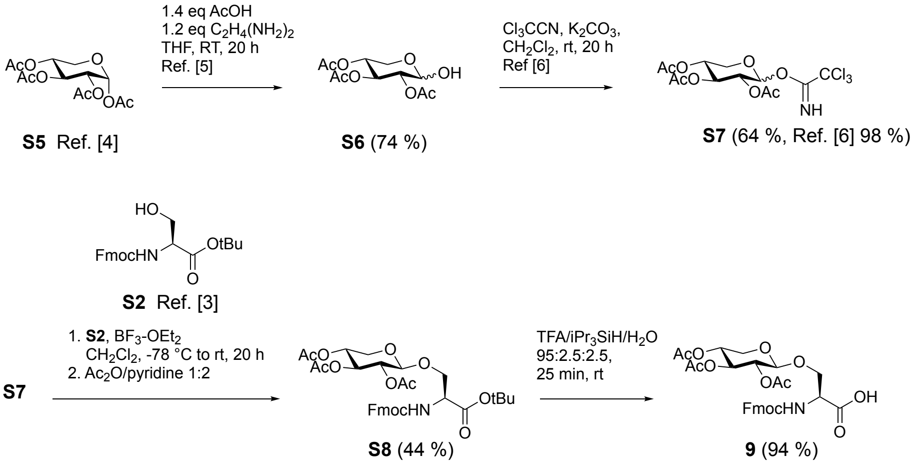

Figure S2: Synthesis of Fmoc-Ser(Ac_3Xyl)-OH **2.**

Compound **S5** was obtained from D-xylose according to Ref.[7] This procedure afforded the desired peracetate **S5** in the pyranose form devoid of furanoid contaminants. Selective anomeric deacetylation was carried out according to Ref.[8] followed by conversion to the trichloroacetimidate **S7**.[9] The glycosylation of **S2**[6] using the imidate **S7** was carried out in analogy to Nakahara.[10] In detail Fmoc-Ser-OtBu **S2** (1 g, 2.6 mmol) trichloroacetimidate **S7** (1.3 g, 3.1 mmol) and freshly dried ground molecular sieves (4 Å) were suspended in absolute DCM (11 mL). The mixture was stirred for 30 min at room temperature followed by cooling to -78 °C. The reaction was started by adding BF_3-Et_2O (77 µL, 0.6 mmol, 0.2 eq). After 2h at -78 °C (TLC: cyclohexane/ethyl acetate 2:1 or DCM/acetone 25:1) more donor **S7** (440 mg, 1 mmol) in 500 µL of absolute DCM was added and the reaction was warmed up to room temperature over 16h. Saturated $NaHCO_3$ (5 mL) was added and after 10 min the stirred suspension was diluted with

ethyl acetate (200 mL) and filtered over celite. The organic phase was extracted with water (2x100 mL) and brine (100 mL), dried over $MgSO_4$ and evaporated to dryness. The chromatographic removal of residual acceptor **S2** was facilitated by acetylation of the residue using acetic anhydride/pyridine 1:2 (5 mL) for 45 min. Subsequently, the volatiles were removed in a rotary evaporator under vacuum after adding toluene (5x20 mL). The residue was dissolved in ethyl acetate (150 mL) and extracted with 1 M HCl and 2 M $KHCO_3$ (75 mL each). After drying over $MgSO_4$ and evaporation of the solvents the product was isolated by flash chromatography (150 g of silica gel, cyclohexane/ethyl acetate 4:1 → 3:1). Yield: 739 mg (1.2 mmol, 44%) of **S8**.

TLC: R_f (**S8**) = 0.55 (cyclohexane/ EtOAc 2:1), 0.55 (DCM/acetone 25:1); R_f (**S2**+Ac) = 0.72 (cyclohexane/EtOAc 2:1), 0.71 (DCM/acetone 25:1); R_f (**S2**) = 0.52 (cyclohexane/EtOAc 2:1), 0.30 (DCM/acetone 25:1).

UPLC: t_{ret} **S8** = 9.51 min; [A] Triart C8, 2.0 × 30 mm, 30-60% MeCN/H_2O + 0.1% HCOOH

ESI-MS of **S8** *m/z* (average isotopes) $C_{33}H_{39}NO_{12}$ (641.67); calculated: 664.24 $[M+Na]^+$, 680.21 $[M+K]^+$; found: 664.27, 680.28.

^1H-NMR ($CDCl_3$, 500 MHz): δ = 7.77 (d, $J_{1,2} = J_{3,4}$ = 7.5 Hz, 2H, Ar), 7.62 (d, 2H, Ar), 7.41 (t, 2H, Ar), 7.33 (t, 2H, Ar), 5.56 (d, $J_{NH,H\alpha}$ = 7.9 Hz, 1H, NH-urethane), 5.15 (t, $J_{3,4} = J_{2,3}$ = 8.3 Hz, 1H, H-3), 4.94-4.84 (m, 2H, H-4, H-2), 4.48 (d, $J_{1,2}$ = 6.5 Hz, 1H, H-1), 4.44-4.34 (m, 3H, Fmoc-CH_2, α-H), 4.27-4.20 (m, 2H, Fmoc-CH, β-Ha), 4.10 (dd, $J_{4,5}$ = 4.8 Hz, $J_{5a,5b}$ = 11.9 Hz, 1H, H-5eq), 3.76 (dd, $J_{\alpha\text{-H},\beta\text{-H}}$ = 2.7 Hz, $J_{\beta\text{-Ha},\beta\text{-Hb}}$ = 10.2 Hz, 1H, β-Hb), 3.36 (dd, $J_{4,5}$ = 8.5 Hz, 1H, H-5ax), 2.06 (s, 3H, OAc), 2.04 (s, 3H, OAc), 2.03 (s, 3H, OAc), 1.47 (s, 9H, tBu).

^{13}C-NMR ($CDCl_3$, 125 MHz) von **38**: δ = 170.16, 170.02, 169.48, 168.58 (C=O), 155.97 (C=O urethane), 143.98, 143.86, 141.47, 141.44, 127.89, 127.24, 125.22, 120.16, 100.95 (C-1, $J_{C\text{-}1,H\text{-}1}$ = 164.1 Hz), 82.96 (C_q, tBu), 71.05 (C-3), 70.58 (C-2), 69.68 (C-β), 68.76 (C-4), 67.17 (Fmoc-CH_2), 61.88 (C-5), 54.84 (C-α), 47.28 (Fmoc-CH), 28.09 (tBu), 20.92, 20.86, 20.77 (OAc).

(Fmoc-Ser(β-Ac_3Xyl)-OH) **9**

Fmoc-Ser(Ac_3Xyl)-OtBu **S8** (450 mg, 701 µmol) was dissolved in 8.8 mL of TFA/iPr_3SiH/H_2O (95/2.5/2.5). The reaction was monitored by TLC (cyclohexane/EtOAc 1:1). After 30 min the volatiles are removed in high vacuum. The residue was purified by flash chromatography (GraceResolv High Resolution, 12 g cartridge, flow rate: 18 mL/min, 0-60% EtOAc/cyclohexane

+ 0.5% AcOH) over 50 min). Fractions containing the desired product were pooled and concentrated. Remaining acetic acid was removed by repeated codistillation with cyclohexane followed by repeated codistillation with DCM in a rotary evaporator. The residue was lyophilized from MeCN/H_2O 1:1. Yield: 387 mg (660 µmol, 94%) of **9**.

TLC: R_f (**9**) = 0.34 (cyclohexane/EtOAc 1:1); R_f (**S8**) = 0.62 (cyclohexane/EtOAc 1:1).

UPLC: t_{ret} **9** = 5.38 min (Triart C8, 2.0 × 30 mm, 30-60% MeCN/H_2O + 0.1% HCOOH).

ESI-MS of **S8** *m/z* (average isotopes) $C_{29}H_{31}NO_{12}$; calculated: 608.17 $[M+Na]^+$, 624.15 $[M+K]^+$; found: 608.08, 624.11.

^{1}H-NMR ($CDCl_3$, 500 MHz) of **9**: δ = 7.77 (d, $J_{1,2} = J_{2,3} = J_{3,4}$ 7.6 Hz, 2H, Ar), 7.61 (m, 2H, Ar), 7.41 (t, 2H, Ar), 7.32 (t, 2H, Ar), 5.65 (d, $J_{NH,H\alpha}$ = 8.2 Hz, 1H, NH-urethane), 5.15 (t, $J_{3,4} = J_{2,3}$ = 8.2 Hz, 1H, H-3), 4.96-4.86 (m, 2H, H-4, H-2), 4.55 (m, 1H, α-H), 4.52 (d, $J_{1,2}$ = 6.2 Hz, 1H, H-1), 4.49-4.38 (m, 2H, Fmoc-CH_2), 4.29 (m, 1H, β-Ha), 4.24 (t, $J_{CH,CH2}$ = 7.0 Hz, 1H, Fmoc-CH), 4.10 (dd, $J_{4,5}$ = 4.9 Hz, $J_{5a,5b}$ = 12.0 Hz, 1H, H-5eq), 3.81 (dd, $J_{\alpha\text{-H},\beta\text{-H}}$ = 3.2 Hz, $J_{\beta\text{-Ha},\beta\text{-Hb}}$ = 10.2 Hz, 1H, β-Hb), 3.38 (dd, $J_{4,5}$ = 8.3 Hz, $J_{5a,5b}$ = 11.9 Hz, 1H, H-5b), 2.06 (s, 3H, OAc), 2.04 (s, 3H, OAc), 2.03 (s, 3H, OAc).

^{13}C-NMR ($CDCl_3$, 125 MHz) of **9**: δ = 172.40, 170.18, 170.11, 169.87 (C=O), 156.27 (C=O urethane), 143.88, 143.74, 141.47, 141.44, 127.92, 127.27, 125.21, 120.17, 100.72 (C-1, $J_{C\text{-}1,H\text{-}1}$ = 164.4 Hz), 70.76 (C-3), 70.51 (C-2), 69.01 (C-β), 68.59 (C-4), 67.41 (Fmoc-CH_2), 61.77 (C-5), 54.02 (C-α), 47.23 (Fmoc-CH), 20.92, 20.82, 20.80 (OAc).

3.2 Synthesis of Bikunin 11-25 Resin 2

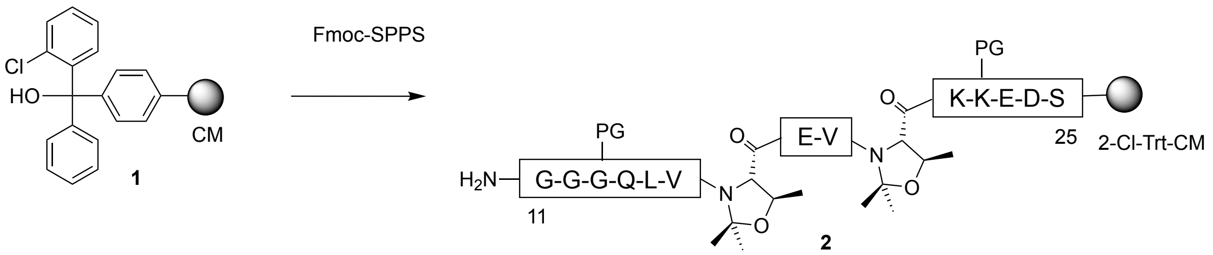

Figure S3: Synthesis of bikunin 11-25 resin **2**

545 mg of resin **1**[1] (degree of substitution 300 µmol/g) were placed in a 40-mL glass tube and treated under argon atmosphere with 8 mL of 10 % AcBr/DCM for 3 h. After removal of the reagents the brominated resin was washed (5 x 10 mL DCM_{abs}). To this resin a solution of 323 mg

(843 μmol) of Fmoc-Ser(t-Bu)-OH and 294 μL (1.69 mmol) of DIPEA in 3 mL of DCM_{abs} were added. After 20 h the slurry was transferred into a 10-mL syringe and the resin was washed with DCM (5 x), a mixture of DCM/methanol/DIPEA (17:2:1, 3x2 min) and DCM (5 x).

The loaded resin was transferred into a 45 mL peptide synthesizer reaction vessel and the peptide chain was elongated automatically. For cleavage of the Fmoc group the general procedure was applied and the amino acid building blocks were coupled under the conditions denoted in table S2 and the general procedure section. The protected amino acids (1.4 mmol) were activated with 579 mg (1.4 mmol) of HCTU and dissolved in 4 mL of 0.4 M DIPEA /DCM. The pseudoproline dipeptide Fmoc-Val-Thr($\Psi^{Me,Me}$pro)-OH (1 mmol) was activated with 533,4 mg (1 mmol) of PyBOP and dissolved in 4 mL of 0.4 M DIPEA/DCM. Resin **2** was obtained after cleavage of the Fmoc group of Gly-11.

Table S1:

position	building block	m [mg]	coupling time [min]	coupling reagent mg
Asp-24	Fmoc-Asp(Ot-Bu)-OH	576	30	HCTU
Glu-23	Fmoc-Glu(Ot-Bu)-OH	596	30	HCTU
Lys-22	Fmoc-Lys(Boc)-OH	656	30	HCTU
Lys-21	Fmoc-Lys(Boc)-OH	656	30	HCTU
Thr-20, Val-19	Fmoc-Val-Thr($\psi^{Me,Me}$pro)-OH	481	45	PyBOP
Glu-18	Fmoc-Glu(Ot-Bu)-OH	596	30	HCTU
Val-17, Thr-16	Fmoc-Val-Thr($\psi^{Me,Me}$pro)-OH	481	45	PyBOP
Leu-15	Fmoc-Leu-OH	495	30	HCTU
Gln-14	Fmoc-Gln(Trt)-OH	855	30	HCTU
Gly-13	Fmoc-Gly-OH	416	30	HCTU
Gly-12	Fmoc-Gly-OH	416	30	HCTU
Gly-11	Fmoc-Gly-OH	416	30	HCTU

3.3 Synthesis of protected Bikunin 1-9 Peptide Acid 6

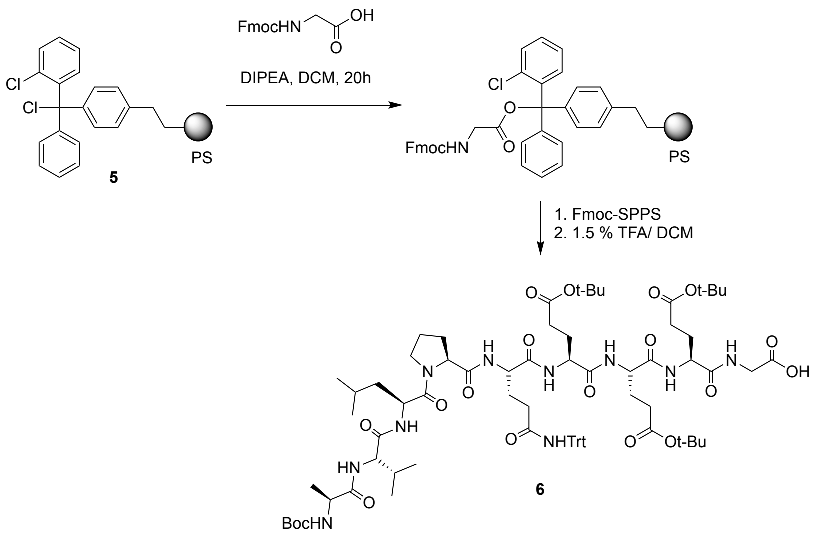

Figure S6: Synthesis of protected bikunin 1-9 peptide acid **6**

Boc-Ala-Val-Leu-Pro-Gln(Trt)-Glu(tBu)-Glu(tBu)-Glu(tBu)-Gly-OH **6**

500 mg of 2-chlorotrityl chloride PS resin **5** (1.0 mmol/g) were placed in a 10-mL syringe. 148.7 mg (0.5 mmol, 1 equiv.) of Fmoc-Gly-OH were dissolved in 2 ml of NMP/DCM (1/1) and 348 µL (2 mmol, 4 equiv.) of DIPEA were added. The slurry was shaken for 20 h. The resin was washed with NMP (2x), DCM (3x) and DCM/methanol/DIPEA (17:2:1, 3x2 min). After washing with NMP (2x) and DCM (3x) the resin was transferred to a 45 mL reaction vessel. The Fmoc group was removed with 20 % piperidine in DMF. The protected amino acids (2.0 mmol) were activated with 827 mg (2.0 mmol) of HCTU. The coupling conditions are listed in table **S2**. For every activation step 3 mL of 0.4 M DIPEA/DMF were added by the synthesizer.

Table S2:

position	building block	m [mg]	coupling time [min]	coupling reagent
Glu-8	Fmoc-Glu(Ot-Bu)-OH	851	30	HCTU
Glu-7	Fmoc-Glu(Ot-Bu)-OH	851	30	HCTU
Glu-6	Fmoc-Glu(Ot-Bu)-OH	851	30	HCTU
Gln-5	Fmoc-Gln(Trt)-OH	1221	30	HCTU
Pro-4	Fmoc-Pro-OH	675	30	HCTU

Leu-3	Fmoc-Leu-OH	707	30	HCTU
Val-2	Fmoc-Val-OH	679	30	HCTU
Ala-1	Fmoc-Ala-OH	623	30	HCTU

An analytical TFA-cleavage (1.5 % TFA in DCM) was carried out and the protected peptide **6** was analyzed by LC-MS. Subsequently, the resin was treated with 4 mL of a mixture consisting of 20 % HFIP, 10 % TFE and 2 % TES in DCM (1 × 20 min, 10 × 2 min) followed by washing with DCM (3 ×). The cleavage solutions were combined in a flask, concentrated in vacuo and dried in high vaccum followed by lyophilization from 1,4-dioxane. Yield: 535.9 mg crude **6** (362 µmol, 72.4 %). LC-MS: Pro C8 S-3 µm, 50 × 2.1 mm, 40-95 % MeCN/H_2O + 0.1 % HCOOH, 0.2 mL/min). ESI-MS of **6** *m/z* (average isotopes) $C_{77}H_{112}N_{10}O_{19}$ (1481.77) calculated: 1482.80 [M+H] $^+$; found: 1483.45 [M+H]$^+$.

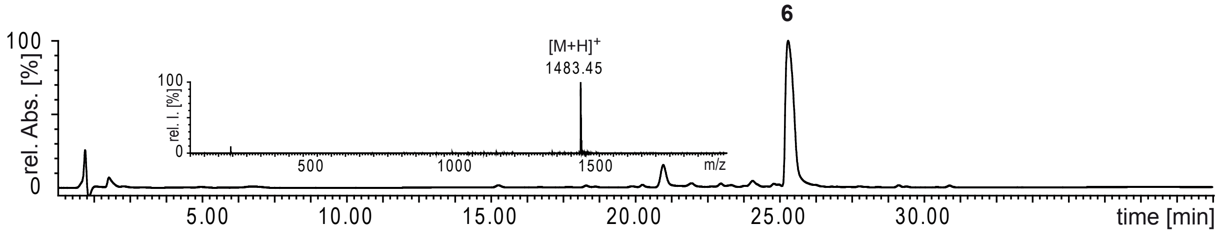

Figure S7: RP-HPLC-MS of protected bikunin 1-9 peptide **6**.

3.4 Convergent Synthesis of Bikunin 1-25 Glycopeptide 7

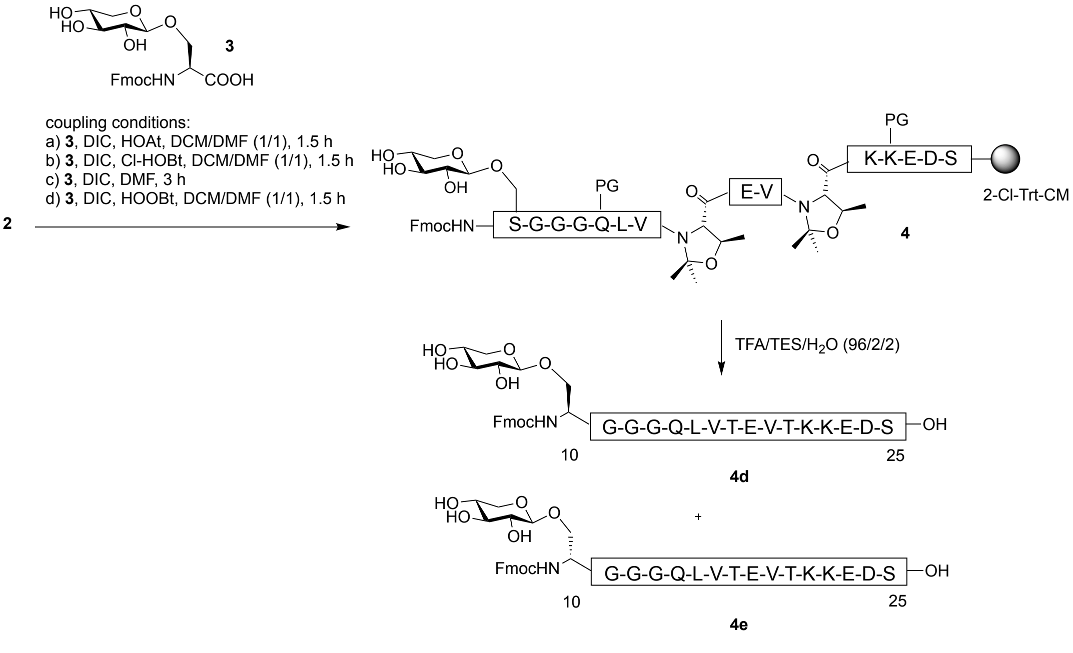

Figure S8: Variation of coupling conditions of Fmoc-Ser(Xyl)-OH **3** to resin **2**

Variation of coupling conditions of Fmoc-Ser(Xyl)-OH **3** to resin **2**

Condition a) Fmoc-Xyl-Ser-OH **3** (3.8 mg, 8.2 µmol) and HOAt (1.1 mg, 8.2 µmol) were dissolved in 0.5 mL of DCM/DMF (1/1) followed by addition of DIC (1.3 µL, 8.2 µmol) and kept for 10 min.

Condition b) Fmoc-Xyl-Ser-OH **3** (3.6 mg, 8.0 µmol) and Cl-HOBt (1.3 mg, 8.0 µmol) were dissolved in 0.5 mL of DCM/DMF (1/1) followed by addition of DIC (1.2 µL, 8.0 µmol) and kept for 10 min.

Condition c) Fmoc-Xyl-Ser-OH **3** (9.0 mg, 20.0 µmol) was dissolved in 0.6 mL of DCM/DMF 5:1 at 0 °C followed by addition of DIC (1.5 µL, 10.0 µmol). After 20 min the solids were removed and the filtrate was concentrated in vacuum. The residue was dissolved in DMF (0.5 mL).

The coupling solutions obtained from conditions a-c were transferred to a syringe containing 20.0 mg of prewashed resin **2** (2 × NMP, 3 × DCM). The suspension was shaken for 1.5 h at ambient temperature. Subsequently, the resin was washed (2 × NMP, 3 × DCM). About 1 mg of resin **4** was submitted to a TFA cleavage (1h, TFA, TES, H_2O, 96:2:2) and peptide **4d** was analyzed by LC-MS (Hydro C18 S-3 µm, 50 × 2.1 mm, 20-80% MeCN/H_2O + 0.1% HCOOH, flow rate: 0.2 mL/min).

ESI-MS of **4d** *m/z* (exact mass) $C_{87}H_{133}N_{19}O_{34}$ (1987.93) calculated: 1988.93 $[M+H]^+$; found: 994.21 $(M+2H)^{2+}$, 1988.50 $(M+H)^+$.

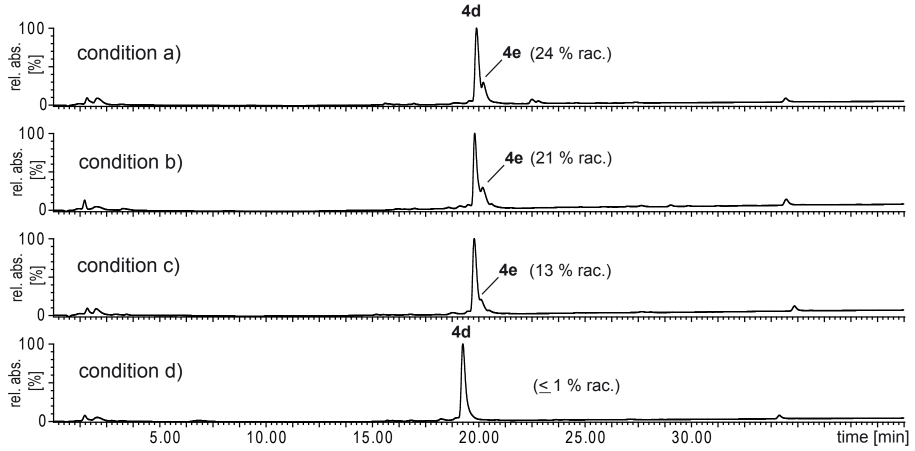

Figure S9: RP-HPLC-MS of deprotected bikunin 10-25 glycopeptide **4d** following activation conditions a-d). It can be assumed that the isobaric peak **4e** corresponds to the epimerized D-Ser(Xyl) residue because of strong activation.[11] The extent of racemization (%) was calculated by integration of the peak areas.

Preparative synthesis: Condition d) Fmoc-Xyl-Ser-OH **3** (54.1 mg, 0.1 mmol) was mixed with a solution of HOOBt[12] (19.1 mg, 0.1 mmol) in 2 mL of DMF/DCM (1/1) followed by addition of DIC (18.4 µL, 0.1 mmol) and kept for 10 min at 0°C. The solution was transferred to a 10 mL syringe containing 328 mg of prewashed resin **2** (2 × NMP, 3 × DCM). The suspension was agitated at ambient temperature for 1.5 h. Subsequently, the resin was washed (2 × NMP, 3 × DCM). About 1 mg of resin **4** was submitted to a TFA cleavage (1h, TFA, TES, H_2O, 96:2:2) and peptide **4d** was analyzed by LC-MS (Hydro C18 S-3 µm, 50 × 2.1 mm; 20-80% MeCN/H_2O + 0.1% HCOOH, flow rate: 0.2 mL/min).

Segment coupling of Bikunin 1-9 peptide acid **6** to resin **4**

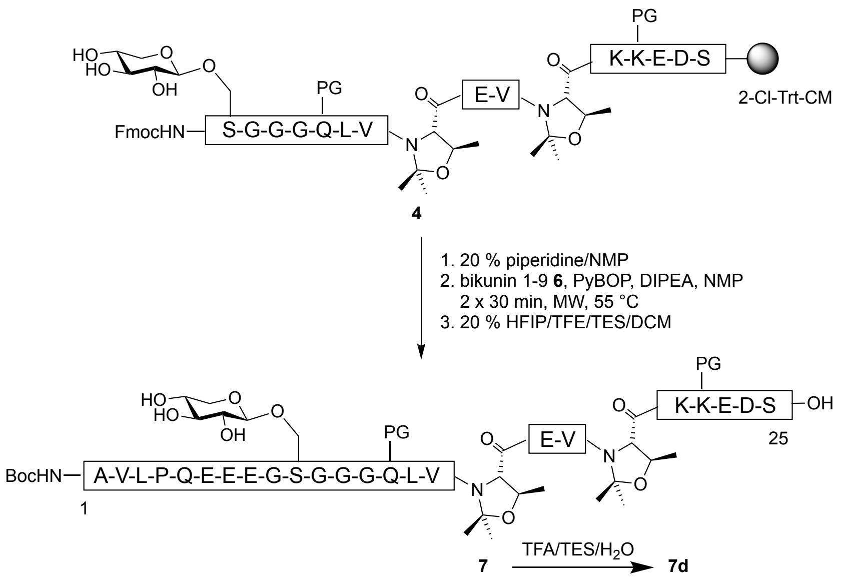

Figure S10: Microwave assisted segment condensation of bikunin 1-9 peptide acid **6** with glycopeptide resin **4**.

The resin **4** obtained after preparative coupling (condition d) was deprotected manually with 20 % piperidine in NMP for 15 min. Subsequently, the resin was washed (4 × NMP, 3 × DCM, 2 × NMP). Bikunin 1-9 peptide acid **6** (174.5 mg, 117 µmol) was dissolved in NMP (5 mL) and PyBOP (61.3 mg, 117 µmol) and DIPEA (40.3 µL, 231 µmol) were added. The mixture was irradiated in a synthesis microwave (55 °C, 70 W) for 30 min under shaking. After adding more PyBOP (61.3 mg, 117 µmol) and DIPEA (40.3 µL, 231 µmol) microwave irradiation (55 °C, 70 W) was continued for 30 min. After a brief cooling period, the resin was washed (2 × NMP, 3 × DCM). The resin was treated with a cleavage cocktail (20 mL, DCM/HFIP/TFE/TES 68:20:10:2)

repeatedly (1 × 20 min, 3 × 2 min each) followed by withing with DCM (3 x). The combined filtrates were collected in a flask, concentrated in vacuum and lyophilized from 1,4 dioxane. Yield: 94 mg of crude peptide **7**. About 1 mg of peptide **7** was submitted to a TFA cleavage (1h, TFA, TES, H_2O, 96:2:2) and peptide **7d** was analyzed by LC-MS (Hydro C18 S-3 µm, 50 × 2.1 mm, 10-70 % MeCN/H_2O + 0.1% HCOOH, flow rate: 0.2 mL/min).

ESI-MS of **7d** *m/z* (exact mass) $C_{113}H_{187}N_{29}O_{48}$ (2718.31) calculated: 2719.32 $[M+H]^{+}$, 1360.16 $[M+2H]^{2+}$, 907.11 $[M+2H]^{3+}$; found: 1360.52 $(M+2H)^{2+}$, 907.52 $(M+3H)^{3+}$.

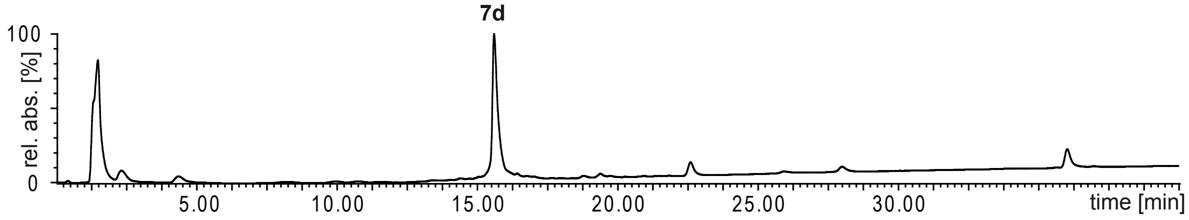

Figure S11: RP-HPLC-MS of crude deprotected bikunin 1-25 glycopeptide **7d**.

3.5 Stepwise Synthesis of Bikunin 1-25 Glycopeptide 7

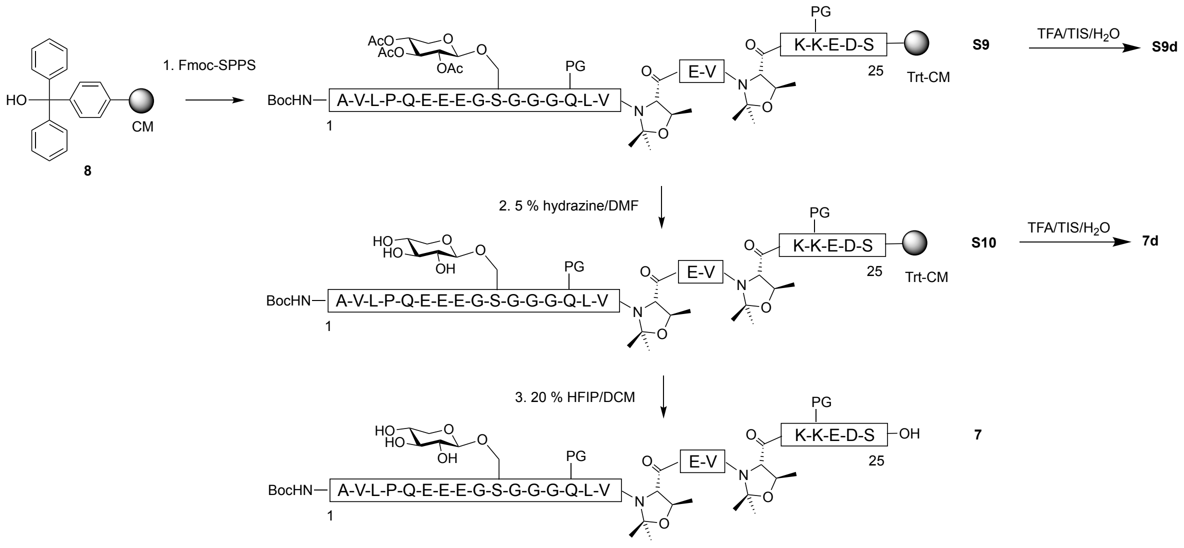

Figure S12: SPPS of bikunin 1-25 glycopeptide **7** on Trt-CM resin **8** using an on-resin deacetylation step.

148 mg of resin **8** (degree of substitution 300 µmol/g) were placed in a 40-mL glass tube and swelled under argon atmosphere with 2.5 mL of DCM_{abs} for 20 min. Subsequently, 2.5 mL of 20 % AcBr/DCM were added. After shaking for 2.5 h the reagents were removed with a syringe and the brominated resin was washed (10 x 10 mL DCM_{abs}). To this resin a solution of 85 mg (222 µmol) of Fmoc-Ser(t-Bu)-OH and 76 µL (445 µmol) of DIPEA in 2 mL of DCM_{abs} were added. After

shaking for 18 h the loaded resin was transferred into a 45 mL peptide synthesizer reaction vessel and the resin was washed repeatedly with DCM (100 mL total). The resin was treated with a mixture of DCM/methanol/DIPEA (10 mL, 17:2:1, 3x5 min each) and then repeatedly washed with DCM (100 mL total).

The peptide chain was elongated automatically. For cleavage of the Fmoc group 2 mL of 20 % piperidine/DMF was used and 2 mL of DMF for each washing step. The amino acid building blocks were coupled under the conditions denoted in table **S3** and the general procedure section. The synthesis was monitored by LC-MS prior and after coupling of the glycosyl amino acid **9** by an analytical test cleavage using 1mg of resin and TFA/iPr$_3$SiH/H$_2$O 95:2.5:2.5 (data not shown).

Table S3:

position	building block	m [mg]	coupling conditions
Asp-24	Fmoc-Asp(O*t*Bu)-OH	91	[a]
Glu-23	Fmoc-Glu(O*t*Bu)-OH	95	[a]
Lys-22	Fmoc-Lys(Boc)-OH	104	[a]
Lys-21	Fmoc-Lys(Boc)-OH	104	[a]
Thr-Val-20,19	Fmoc-Val-Thr($\psi^{Me,Me}$Pro)-OH	64	[b]
Glu-18	Fmoc-Glu(O*t*Bu)-OH	95	[a]
Thr-Val-17,16	Fmoc-Val-Thr($\psi^{Me,Me}$Pro)-OH	64	[b]
Leu-15	Fmoc-Leu-OH	79	[a]
Gln-14	Fmoc-Gln(Trt)-OH	136	[a]
Gly-13	Fmoc-Gly-OH	66	[a]
Gly-12	Fmoc-Gly-OH	66	[a]
Gly-11	Fmoc-Gly-OH	66	[a]
Ser-10	Fmoc-Ser(Ac$_3$Xyl)-OH **9**	52	[c]

Gly-9	Fmoc-Gly-OH	66	[a]
Glu-8	Fmoc-Glu(O*t*Bu)-OH	95	[a]
Glu-7	Fmoc-Glu(O*t*Bu)-OH	95	[a]
Glu-6	Fmoc-Glu(O*t*Bu)-OH	95	[a]
Gln-5	Fmoc-Gln(Trt)-OH	136	[a]
Pro-4	Fmoc-Pro-OH	75	[a]
Leu-3	Fmoc-Leu-OH	79	[a]
Val-2	Fmoc-Val-OH	75	[a]
Ala-1	Boc-Ala-OH	42	[a]

[a] The amino acid building block (222 µmol) and HCTU (88 mg, 213 µmol) were dissolved in 2 mL of 0.24 M DIPEA/DMF (480 µmol DIPEA) under stirring for 4 min. coupling time: 45 min.

[b] The amino acid building block (133 µmol) and PyBOP (69 mg, 133 µmol) were dissolved in 1 mL of 0.2 M DIPEA/DMF (240 µmol DIPEA) under stirring for 4 min. coupling time: 90 min.

[c] Fmoc-Ser(Ac_3Xyl)-OH **9** (52 mg, 89 µmol) and HOOBt (15 mg, 90 µmol) were dissolved in 1.8 mL of DCM/DMF 1:1 (abs.) under stirring. After cooling the yellowish solution to 0 °C, DIC (14 µL, 89 µmol) was added and the mixture was allowed to warm up to room temperature over 10 min. The solution was diluted with 1 mL of DCM/DMF 1:1 (abs.) and added to the resin. coupling time: 3 h.

After completion of the solid phase synthesis the resin was washed with DMF (5 x 2 mL) and DCM (5 x 2 mL) and dried in high vacuum. Yield: 356 mg of resin **S9** (degree of loading 135 µmol/g). The synthesis was monitored after an analytical scale cleavage of resin **S9** with TFA/iPr_3SiH/H_2O 95:2.5:2.5 by LC-MS (Hydrosphere C18, 2.0 × 30 mm, 5-35% MeCN/H_2O +0.1% HCOOH). ESI-MS of **S9d**: *m/z* (average isotopes) $C_{119}H_{193}N_{29}O_{51}$ (2846.01) calculated: 2847.01 $[M+H]^+$, 1424.01 $[M+2H]^{2+}$, 949.68 $[M+3H]^3$; found: 2847.85, 1423.88, 949.57.

The Bikunin 1-25(Ac_3Xyl)-Trt-CM-resin **S9** (356 mg, degree of loading 135 µmol/g) was placed in a PP-syringe equipped with a frit and swelled with DCM for 50 min. The solvent was discarded, and a total reaction volume of 10 mL was established with 5 % hydrazine hydrate in DMF. The mixture

was agitated for 3.5 h and subsequently washed with DMF (5 x 10 mL) and DCM (5 x 10 mL) and dried in high vacuum. The deacetylation was monitored after an analytical scale cleavage of the resin with TFA/iPr$_3$SiH/H$_2$O 95:2.5:2.5 by LC-MS (Hydrosphere C18, 2.0 × 30 mm, 5-35% MeCN/H$_2$O +0.1% HCOOH). ESI-MS of **7d**: *m/z* (average isotopes) $C_{113}H_{187}N_{29}O_{48}$ (2719.89 g/mol) calculated: 2720.90 $[M+H]^+$, 1360.95 $[M+2H]^{2+}$, 907.64 $[M+3H]^{3+}$; found: 1360.22, 2720.59, 907.47.

After deacetylation the resin **S10** was swelled in DCM for 30 min and subsequently shaken with the cleavage cocktail (20 % HFIP/DCM, 10 mL, 10 x 2 min). The filtrates were concentrated and for complete removal of HFIP the residue was taken up in 50 mL of DCM (10 x) and concentrated in vacuum to dryness. The crude peptide was lyophilized from dioxane. Yield: 184 mg of crude **7** (46 µmol, 97%). LC-MS analysis was carried out with protected **7** as well as after analytical deprotection (**7d**) with TFA/iPr$_3$SiH/H$_2$O 95:2.5:2.5.

LC-MS of **7** (Triart C8, 2.0 × 30 mm, 70-95% MeCN/H$_2$O +0.1% HCOOH). ESI-MS of **7**: *m/z* (average isotopes) $C_{200}H_{303}N_{29}O_{54}$ (3977.77) calculated: 4000.76 $[M+Na]^+$; found: 3998.23.

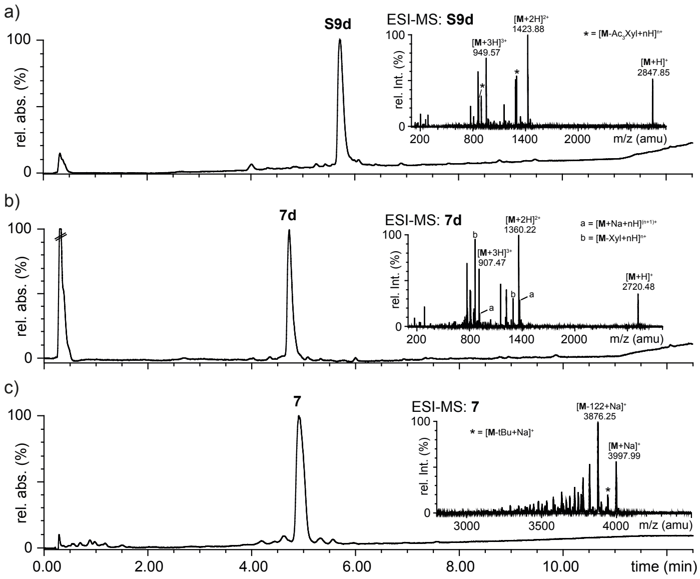

Figure S13: RP-HPLC-MS of crude bikunin 1-25 glycopeptides: a) **S9d**; b) **7d**.; c) **7**.

3.6 Synthesis of Bikunin 1-25 Glycopeptide Thioester 11 and Hydrazide A

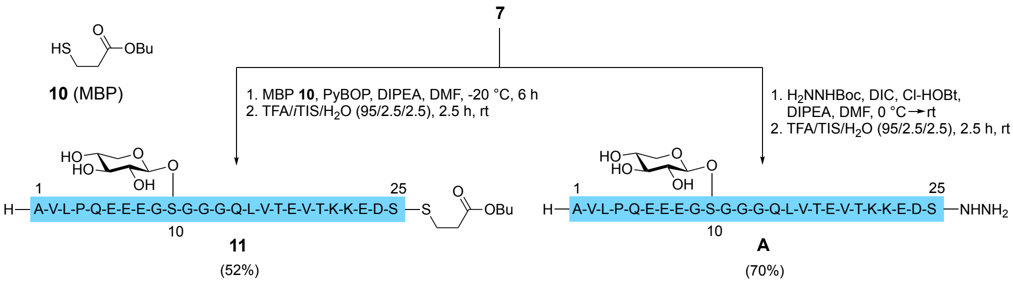

Figure S14: Synthesis of Bikunin 1-25 Glycopeptide Thioester **11** and Hydrazide **A** from **7**.

Thioester **11**

Protected bikunin 1-25 glycopeptide **7** (50 mg, 13 µmol) was dissolved in dry DMF (200 µL), *n*-Butyl-3-mercaptopropionate **10** (62 µL, 380 µmol) was added and the solution was cooled to -18 °C. In parallel PyBOP (33 mg, 63 µmol) and dry DIPEA (11 µL, 64 µmol) were dissolved in dry DMF[13] (400 µL) and after cooling to -18 °C the two solutions were combined. The reaction was diluted with dry DMF (242 µL, -18 °C). Reaction progress at -18 °C was monitored by TLC: (DCM/MeOH 10:1; R_f (**7**) = 0.15, R_f (Thioester) = 0.57). After complete conversion of **7** (5.5 h) the reaction was brought to pH 5 by adding 7 µL of TFA und 3 µL of DIPEA and warmed up to room temperature. The mixture was concentrated to 50-100 µL and the cleavage cocktail TFA/TIS/H_2O 95:2.5:2.5 (36 mL) was added. After 2.5 h at room temperature the mixture was concentrated in high vacuum to 4 mL and cold ether (42 mL, -25 °C) was added. The precipitated glycopeptide thioester was centrifuged (4 min, 4000g) and the pellet was washed twice with cold ether. The pellet was dried in high vacuum, taken up in 20% MeCN/H_2O +0.1% TFA (5 mL) and lyophilized. The residue was purified by HPLC (Supelco Ascentis C18, 150 × 10 mm, 15-30% MeCN/H_2O +0.1% TFA, flow rate: 3 mL/min. Yield: 18.8 mg of **11** (6.6 µmol, 52%). LC-MS of **11** (Hydrosphere C18, 2.0 × 30 mm, 10-30% MeCN/H_2O +0.1% HCOOH). ESI-MS of **11**: *m/z* (average isotopes) $C_{120}H_{199}N_{29}O_{49}S$ (2864.13) calculated: 2865.13 $[M+H]^+$, 1433.07 $[M+2H]^{2+}$, 955.72 $[M+3H]^{3+}$; found: 2865.69, 1432.75, 955.82.

Hydrazide **A**

Protected bikunin 1-25 glycopeptide **7** (40 mg, 10 µmol) was dissolved in dry DMF (150 µL). *Tert*-butylcarbazate (8 mg, 61 µmol), Cl-HOBt (6.8 mg, 40 µmol) and DIPEA (9.4 µL, 55 µmol) in 50 µL DMF were added. The mixture was diluted with 50 µL of DMF and cooled to 0 °C. After adding DIC (6.2 µL, 40 µmol) the reaction was allowed to warm up to room temperature. The reaction progress was monitored by TLC: (DCM/MeOH 10:1) and LC-MS (YMC Triart C8, 70-95% MeCN/H_2O +0.1% HCOOH). After complete conversion of **7** (24 h) the reaction was cooled and diluted with cleavage cocktail TFA/TIS/H_2O 95:2.5:2.5 (13 mL, cooled to 0 °C). After 2.5 h at room temperature the mixture was concentrated in high vacuum to 1.5 mL and cold ether (30 mL, -25 °C) was added. The precipitated glycopeptide hydrazide was centrifuged (4 min, 4000g) and the pellet was washed twice with cold ether. The pellet was taken up in water (10 mL), passed through a Luer filter (0.2 µm) and lyophilized. The residue (35 mg) was dissolved in 5% MeCN/H_2O +0.1% TFA and purified by HPLC (Pro C8, 250 × 20 mm, 5-25% MeCN/H_2O +0.1% TFA, flow rate: 3 mL/min). Yield: 19.4 mg of **A** (7.1 µmol, 70%). LC-MS of **A** (Hydrosphere C18, 2.0 × 30 mm, 5-35% MeCN/H_2O +0.1% HCOOH). ESI-MS of **A**:

m/z (average isotopes) $C_{113}H_{189}N_{31}O_{47}$ (2733.93) calculated: 2734.93 $[M+H]^+$, 1367.97 $[M+2H]^{2+}$, 912.32 $[M+3H]^{3+}$; found: 2741.16, 1370.46, 914.37.

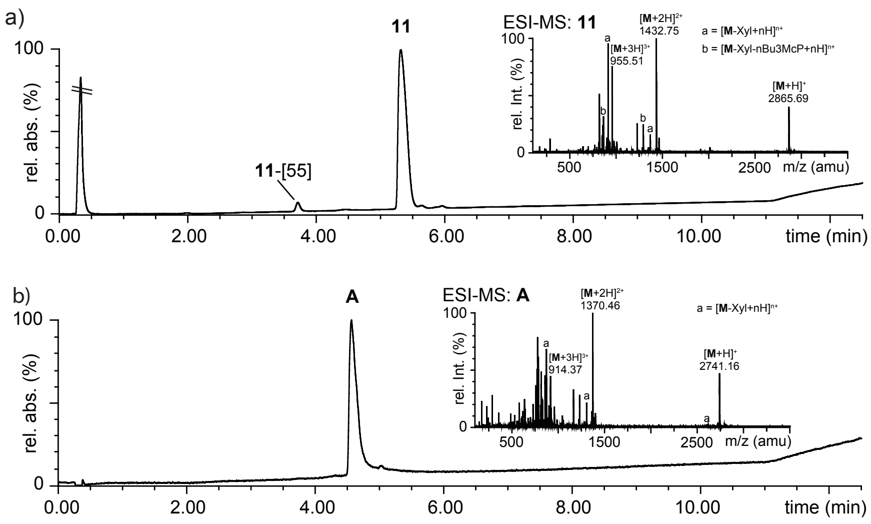

Figure S15: RP-HPLC-MS of purified a) 1-25 glycopeptide thioester **11** and b) hydrazide **A**.

4. Synthesis of Bikunin 26-50 Glycopeptide Hydrazide B

4.1 Synthesis of Bikunin 26-50 Hydrazide 12

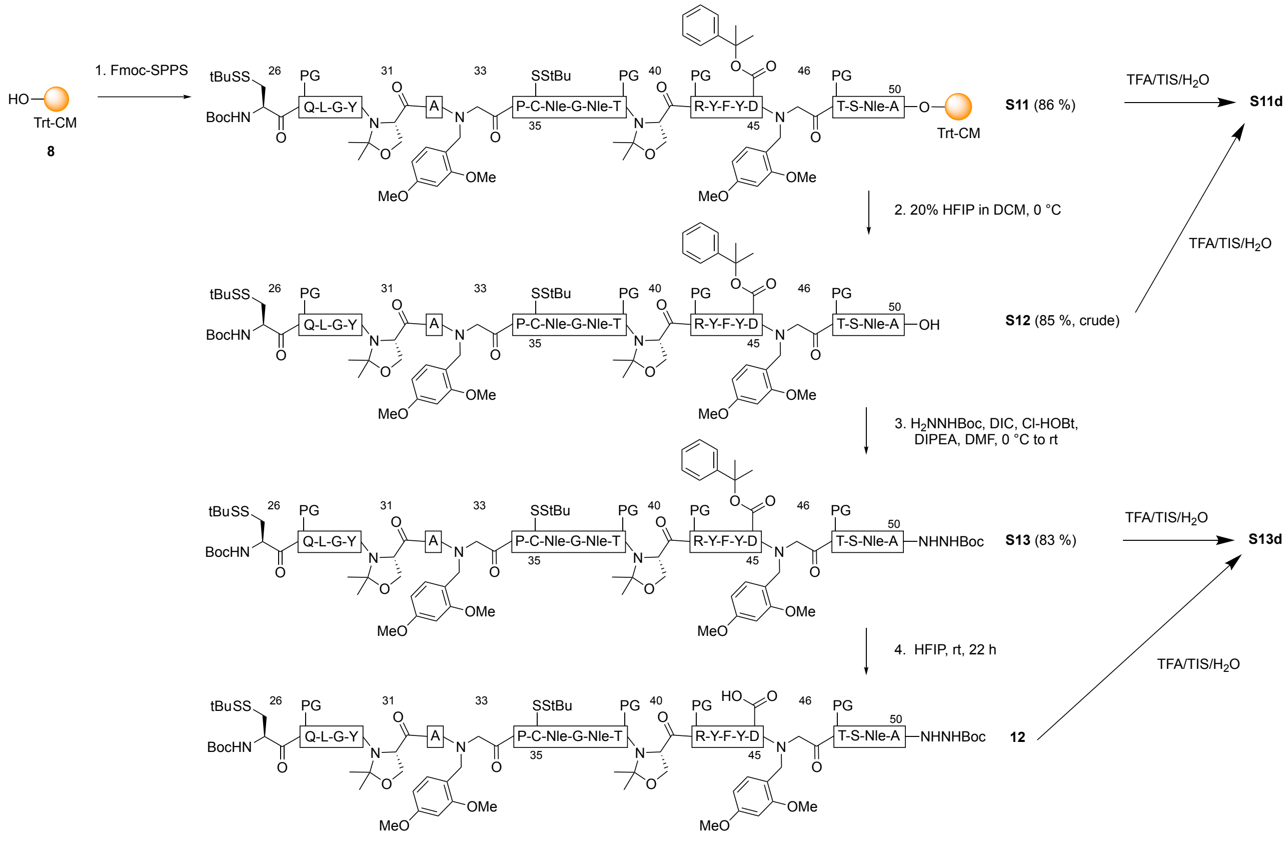

Figure S16: Synthesis of selectively deprotected Bikunin 26-50 Hydrazide **12**.

400 mg of resin **8** (degree of substitution 300 µmol/g) were placed in a 20-mL glass tube and swelled under argon atmosphere with 6 mL of DCM_{abs} for 20 min. Subsequently, 3 mL of 20 % AcBr/DCM were added. After shaking for 2.5 h the reagents were removed with a syringe and the brominated resin was washed (10 x 10 mL DCM_{abs}). To this resin a solution of Fmoc-Ala-OH (186.8 mg, 0.6 mmol) and $DIPEA_{abs}$ (204 µL, 1.2 mmol) in 3 mL of DCM_{abs} was added. After shaking for 15 h the loaded resin was transferred into a 45 mL peptide synthesizer reaction vessel and the resin was washed repeatedly with DCM (100 mL total). The resin was treated with a mixture of DCM/methanol/DIPEA (10 mL, 17:2:1, 3x5 min each) and then repeatedly washed with DCM (100 mL total).

The peptide chain was elongated automatically. For cleavage of the Fmoc group 6 mL of 20 % piperidine/DMF was used and 6 mL of DMF for each washing step. The amino acid building blocks were coupled under the conditions denoted in table **S4** and the general procedure section.

Table S4:

position	building block	m [mg]	coupling conditions
Nle-49	Fmoc-Nle-OH	212	[a]
Ser-48	Fmoc-Ser(*t*Bu)-OH	230	[a]
Thr-47	Fmoc-Thr(*t*Bu)-OH	239	[a]
Gly-46	Fmoc-(Dmb)Gly-OH	161	[b]
Asp-45	Fmoc-Asp(OPhiPr)-OH	171	[b]
Tyr-44	Fmoc-Tyr(*t*Bu)-OH	276	[a]
Phe-43	Fmoc-Phe-OH	233	[a]
Tyr-42	Fmoc-Tyr(*t*Bu)-OH	276	[a]
Arg-41	Fmoc-Arg(Pbf)-OH	389	[a]
Ser-Thr-40,39	Fmoc-Thr(*t*Bu)-Ser($\psi^{Me,Me}$Pro)-OH	189	[b]

Nle-38	Fmoc-Nle-OH	212	[a]
Gly-37	Fmoc-Gly-OH	178	[a]
Nle-36	Fmoc-Nle-OH	212	[a]
Cys-35	Fmoc-Cys(S*t*Bu)-OH	518	[c]
Pro-34	Fmoc-Pro-OH	202	[a]
Gly-33	Fmoc-(Dmb)Gly-OH	161	[b]
Ala-32	Fmoc-Ala-OH	187	[a]
Ser-Tyr-31,30	Fmoc-Tyr(tBu)-Ser($\psi^{Me,Me}$Pro)-OH	211	[b]
Gly-29	Fmoc-Gly-OH	178	[a]
Leu-28	Fmoc-Leu-OH	212	[a]
Gln-27	Fmoc-Gln(Trt)-OH	366	[a]
Cys-26	Boc-Cys(S*t*Bu)-OH	371	[c]

[a] The amino acid building block (0.6 mmol) and HCTU (238 mg, 0.58 mmol) were dissolved in 6 mL of 0.2 M DIPEA/DMF (1.2 mmol DIPEA) under stirring for 4 min. coupling time: 45 min.

[b] The amino acid building block (360 µmol) and PyBOP (187 mg, 360 µmol) were dissolved in 6 mL of 0.2 M DIPEA/DMF (1.2 mmol DIPEA) under stirring for 4 min followed by addition of 2.5 mL of DMF. coupling time: 90 min.

[c] The symmetrical anhydride was generated from the amino acid building block (1.2 mmol) and DIC (0.6 mmol) according to Ref. [9]

After completion of the solid phase synthesis the resin **S11** was washed with DMF (5 x 6 mL) and DCM (5 x 6 mL) and dried in high vacuum. Yield: 778 mg of resin **S11** (degree of loading 132 µmol/g, 86 %). The synthesis was monitored after an analytical scale cleavage of resin **S9** with TFA/iPr$_3$SiH/H$_2$O 95:2.5:2.5 by LC-MS (Hydrosphere C18, 2.0 × 30 mm, 20-40% MeCN/H$_2$O +0.1% HCOOH). ESI-MS of **S11d**: *m/z* (average isotopes) $C_{125}H_{191}N_{29}O_{37}S_4$ (2820,31) calculated: 2821.32 $[M+H]^+$, 1411.16 $[M+2H]^{2+}$; found: 2820.77, 1410.31.

Dried resin **S11** (199 mg) was swelled in DCM (10 mL) for 20 min and the DCM was filtered off. Subsequently the resin was treated with cleavage cocktail cooled to 0 °C (20 % HFIP/DCM, 2 mL, 10 x 1 min). The filtrates were collected and kept at 0 °C in a 500 mL flask and the resin was washed with cold DCM (3 mL, 10× 30 s). The combined washings were concentrated to dryness on a rotary evaporator keeping a bath temperature of 0-5 °C. For complete removal of HFIP the residue was taken up in 20 mL of DCM (6 x) and concentrated as above. The residue was dried in high vacuum and lyophilized from dioxane. Yield: 95 mg of crude **S12**, (22 µmol, 85%). TLC: R_f **S12** = 0.42 (DCM/MeOH 15:1). LC-MS analysis was carried out with protected **S12** as well as after analytical deprotection (**S11d**) with TFA/iPr_3SiH/H_2O 95:2.5:2.5. LC-MS of **S12** (Triart C8, 2.0 × 30 mm, 85-95% $MeCN/H_2O$ +0.1% HCOOH). ESI-MS of **S12**: *m/z* (average isotopes) $C_{219}H_{315}N_{29}O_{46}S_5$ (4250.39 g/mol) calculated: 4251.39 $[M+H]^+$; found: 4250.02.

Hydrazide **S13**

Protected bikunin 26-50 peptide acid **S12** (89 mg (21 µmol) was dissolved in dry DCM (400 µL). *Tert*-butylcarbazate (17 mg, 126 µmol), Cl-HOBt (14 mg, 84 µmol) and DIPEA (18 µL (108 µmol) in dry DCM (100 µL) were added. The mixture was diluted with dry DCM (200 µL) and cooled to 0 °C. After adding DIC (14 µL, 90 µmol) the reaction was allowed to warm up to room temperature. The reaction progress was monitored by TLC: (DCM/MeOH 15:1). After 5 h the mixture was cooled to 0 °C and a second portion of *tert*-butylcarbazate (17 mg, 126 µmol), Cl-HOBt (14 mg, 84 µmol) and DIPEA (18 µL (108 µmol) in dry DCM (100 µL) was added. Subsequently, DIC (14 µL, 90 µmol) was added and the reaction was allowed to warm up to room temperature. After 12 h the conversion was complete and the reaction was diluted with DCM (100 mL) and extracted with 2 M $KHCO_3$ (50 mL). The aqueous phase was extracted with DCM. The combined organic phases were dried over $MgSO_4$ and concentrated. The residue was purified by flash chromatography (12 g cartridge Grace Reveleris Silica 40 µm, flow rate: 30 mL/min, 0-10% MeOH in DCM over 30 min). Fractions containing the product were pooled, concentrated and lyophilized from dioxane. Yield: 76 mg of **S13** (17 µmol, 83%). LC-MS analysis was carried out with protected **S13** as well as after analytical deprotection (**S13d**) with TFA/iPr_3SiH/H_2O 95:2.5:2.5. LC-MS of **S13** (Triart C8, 2.0×30 mm, 85-95% $MeCN/H_2O$+0.1% HCOOH). ESI-MS of **S13**: *m/z* (average isotopes) $C_{224}H_{325}N_{31}O_{47}S_5$ (4364.53) calculated: 4365.54 $[M+H]^+$; found: 4365.89. ESI-MS of **S13d**: *m/z* (average isotopes) $C_{125}H_{193}N_{31}O_{36}S_4$ (2834.34) calculated: 2835.35 $[M+H]^+$, 1418.18 $[M+2H]^{2+}$; found: 2833.64, 1417.26.

TLC: R_f (**S13**) = 0.46 (DCM/MeOH 15:1); R_f (**S12**) = 0.42 (DCM/MeOH 15:1)

Aspartylpeptide **12**

Bikunin 26-50 hydrazide **S13** (120 mg, 28 µmol) was dissolved in 14 mL of HFIP (2.5 mM **S13**) and kept at room temperature. Reaction progress was monitored by LC-MS (YMC Triart C8, , 2.0 × 30 mm, 85-95% MeCN/H_2O+0.1% HCOOH). After 22 h complete conversion of the starting material **S13** was observed, and the solution was concentrated to dryness. For complete removal of HFIP the residue was taken up in 100 mL of DCM (10x) and concentrated as above. The remainder was dried in high vacuum and lyophilized from dioxane. Yield: 114 mg of crude **12** (27 µmol, 97%). LC-MS analysis was carried out with protected **12** as well as after analytical deprotection (**S13d**) with TFA/iPr$_3$SiH/H_2O 95:2.5:2.5. LC-MS of **12** (Triart C8, 2.0 × 30 mm, 85-95% MeCN/H_2O +0.1% HCOOH). ESI-MS of **12**: *m/z* (average isotopes) $C_{215}H_{315}N_{31}O_{47}S_5$ (*M* = 4246.36 g/mol) calculated: 4247.37 $[M+H]^+$; found: 4246.69.

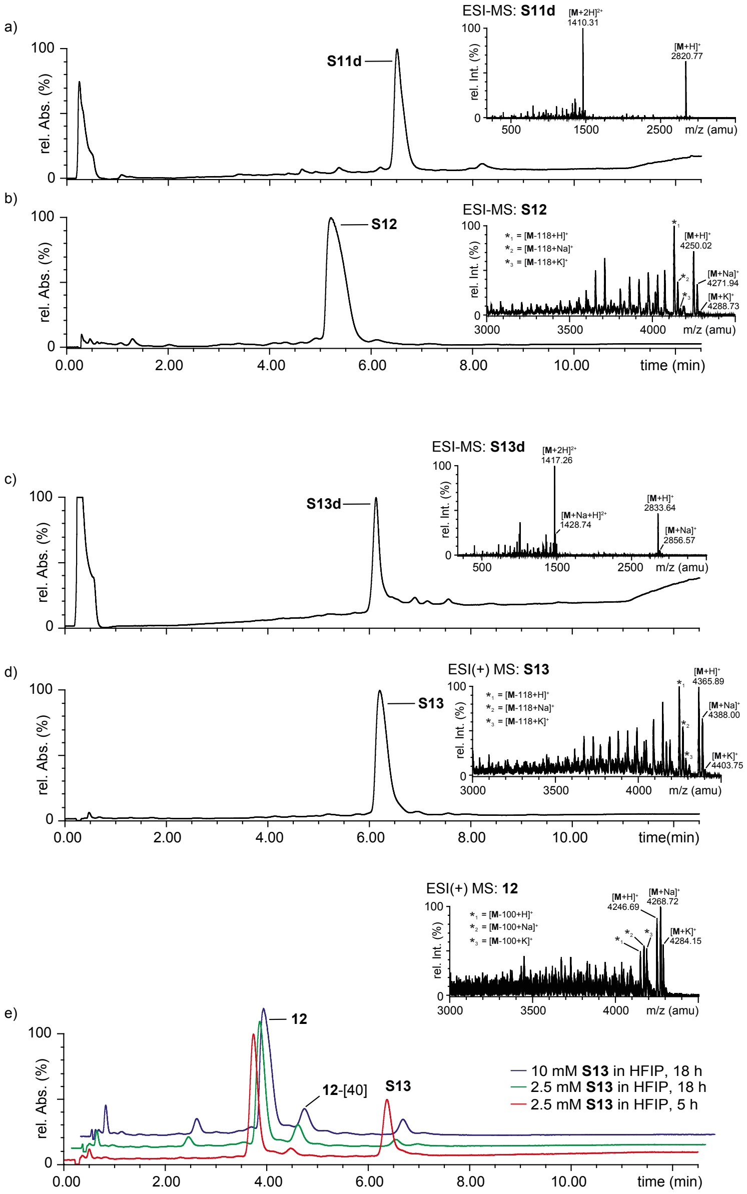

Figure S17: RP-HPLC-MS of a) deprotected 26-50 peptide **S11d**; b) protected 26-50 peptide acid **S12**; c) deprotected 26-50 peptide hydrazide **S13d**; d) protected 26-50 peptide hydrazide **S13**; e) selective removal of the Phipr ester from **S13** in HFIP at different concentrations yielding aspartyl peptide hydrazide 26-50 **12**.

4.2 Synthesis of Bikunin 26-50 Glycopeptide Hydrazide B

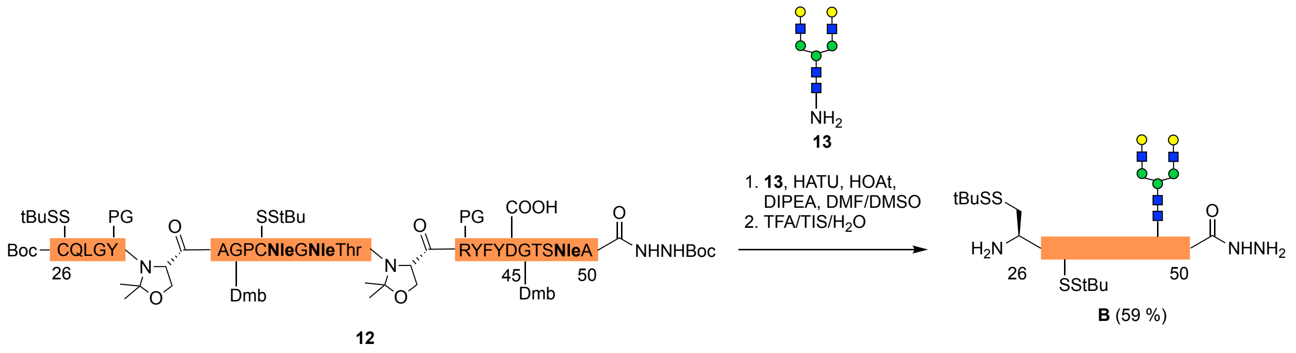

Figure S18: Synthesis of Bikunin 26-50 glycopeptide hydrazide **B from 12** and glycosylamine **13**.[14]

To aspartylpeptide-hydrazide **12** (7.7 mg, 1.8 µmol) was added a mixture of HATU (1.4 mg, 3.6 µmol), HOAt (0.5 mg, 3.6 µmol) and DIPEA (1.4 µL, 8.4 µmol) in 40 µL of DMF/DMSO 1:1 by means of adding in 40 µL of the following stock solution: 5.5 mg of HATU, 2 mg of HOAt, 5.7 µL of DIPEA in 154 µL of DMF/DMSO 1:1. The mixture was vortexed and kept for 10 min at ambient temperature. The activated peptide was added to the freshly prepared glycosylamine **13** (2.3 mg, 1.4 µmol). The suspension was diluted with 21 µL of DMF/DMSO 1:1 and agitated for 16 h unter an argon atmosphere. Susequently, the coupling was monitored by LC-MS after an analytical scale deprotection with TFA/Et_3SiH/H_2O 96:2:2.

Preparative deprotection was carried out by adding 5 mL of cold (0 °C) TFA/Et_3SiH/H_2O 96:2:2 to the cooled reaction mixture (0 °C). After shaking for 2.5 h at ambient temperature the solution was concentrated in vacuo to approximately 0.5 mL and14 mL of cold diethylether (-30 °C) were added. The suspension was cleared by centrifugation and the supernatant was decanted. This procedure was repeated twice with 14 mL of cold diethylether. The pellet was dried in high vacuum, dissolved in 25% MeCN/H_2O +0.1% TFA and purified by RP-HPLC (*Supelco* Ascentis C18, 150 × 10 mm, 25-45% MeCN/H_2O +0.1% TFA, flow rate: 3 mL/min). Fractions containing the product **B** were pooled and lyophilized.

Yield: 2.6 mg of **B** (0.6 µmol, 97%). LC-MS of **b** (Hydrosphere C18, 2.0 × 30 mm, 10-40% MeCN/H_2O +0.1% HCOOH). ESI-MS of **B**: *m/z* (average isotopes) $C_{187}H_{296}N_{36}O_{80}S_4$ (M = 4456.84) calculated: 1486.62 $[M+3H]^{3+}$, 2229.43 $[M+2H]^{2+}$, 4457.84 $[M+H]^{+}$; found: 1486.63, 2229.92, 4457.92.

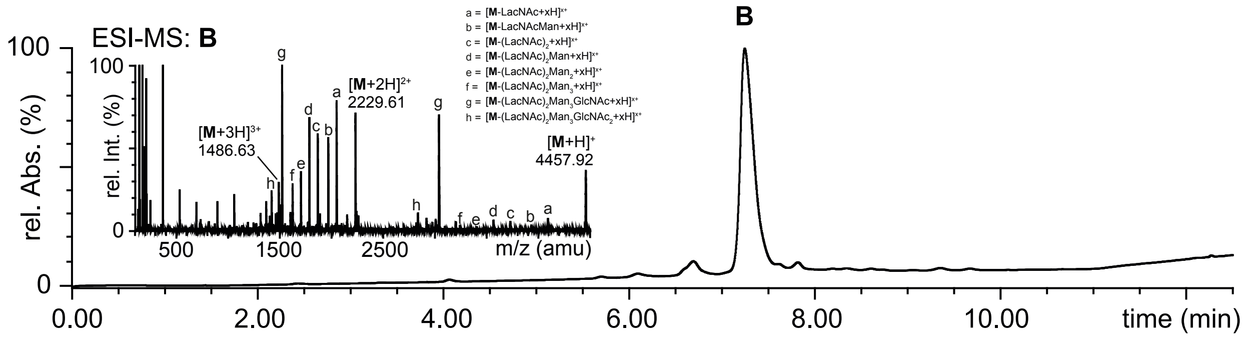

Figure S19: RP-HPLC-MS of purified glycopeptide hydrazide 26-50 **B**.

5. Enzymatic Elongation of Bikunin 1-25 Glycopeptides

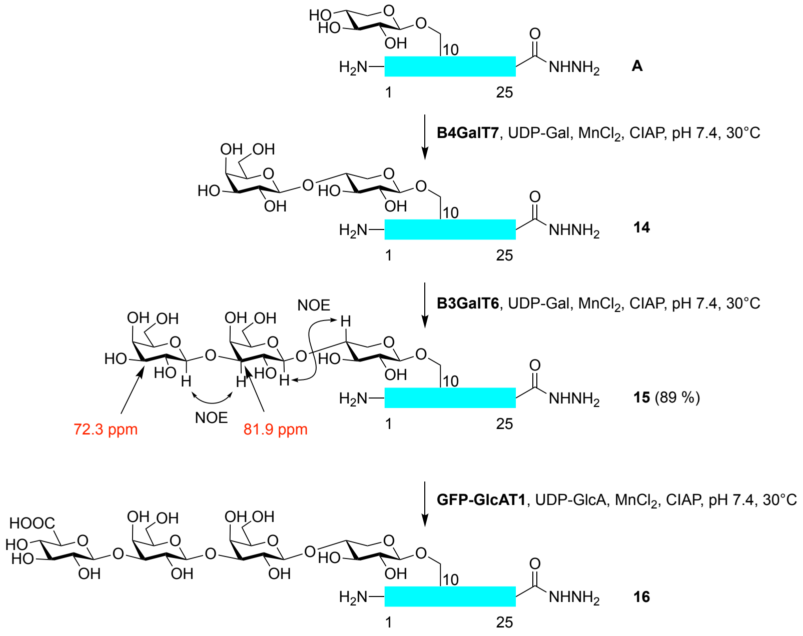

Figure S20: Enzymatic elongation of Bikunin 1-25 hydrazide **A**. using recombinant glycosyltransferases.

5.1 Synthesis of Bikunin 1-25 (Gal-Gal-Xyl)-Glycopeptide **15**

Glycopeptide hydrazide **A** (2.1 mg, 0.77 µmol) was dissolved in 1 mL of recombinant human β4GalT7 expressed in Hi5 cells,[15] 5.5 mU, 1 M NaCl, Bis-Tris, pH 7). Subsequently, BSA (3.0 µL of a 10 mg/mL stock solution), alkaline phosphatase (5.0 µL, 60 U), $MnCl_2$ (2.4 µL of a 0.5 M stock solution) and UDP-Gal (1.9 mg, 3.08 µmol) were added. The reaction was incubated at 30°C for 18 h. The reaction mixture was purified by gel filtration (Superdex Peptide PE 30/100 300 × 10 mm, 30

% MeCN/H_2O + 0.1 % TFA, flow rate 0.6 mL/min). Yield: 2.1 mg of **15** (0.69 µmol, 89 %). LC-MS of **15** (Hydrosphere C18 S-3 µm, 2.1 × 50 mm, 10-50% MeCN/H_2O +0.1% HCOOH). ESI-MS of **15**: *m/z* (average isotopes) $C_{125}H_{209}N_{31}O_{57}$ (3058,21), calculated: 1020.41 $[M+3H]^{3+}$, 1530.11 $[M+2H]^{2+}$; found: 1019.78, 1529.81.

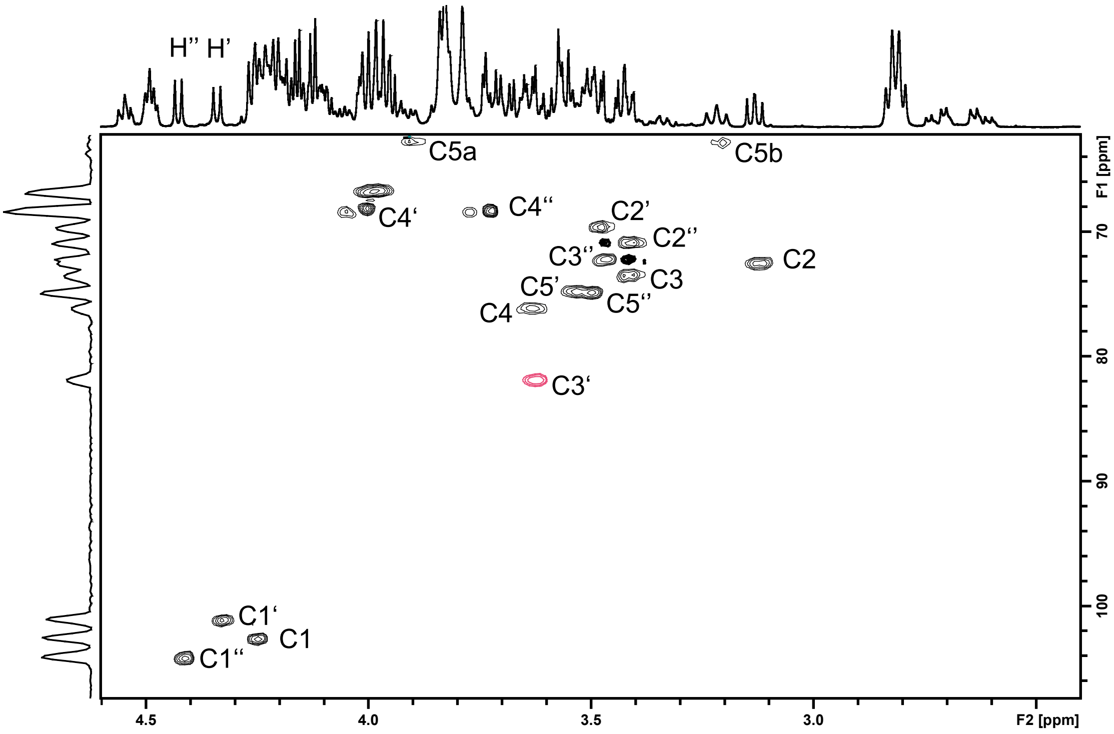

Figure S21: Zoom of a 500 MHz-NMR HSQC-spectrum of **15** recorded in D_2O.

5.2 Synthesis of Bikunin 1-25 (GlcA-Gal-Gal-Xyl)-Glycopeptide 16

0.1 mg (32.7 nmol) of glycopeptide **15** was dissolved in 50 µL of recombinant GFP-GlcAT1 expressed in HEK cells[16] (0.9 mg/mL in 25 mM HEPES, 300 mM NaCl and 300 mM imidazole, pH 7). Subsequently, BSA (2.0 µL of a 10 mg/mL stock solution), alkaline phosphatase (2.0 µL, 24 U), $MnCl_2$ (0.5 µL of a 0.5 M stock solution) and UDP-GlcA trisodium salt (2.0 µL of a 50 mg/mL stock solution, 155 nmol) were added. The reaction was incubated at 30°C for 18 h and analyzed by LC-MS (Hydrosphere C18 S-3 µm, 2.1 × 50 mm, 10-50% MeCN/H_2O +0.1% HCOOH). ESI-MS of **16**: *m/z* (average isotopes) $C_{131}H_{217}N_{31}O_{63}$ (3234.33), calculated: 1079,12 $[M+3H]^{3+}$, 1618.18 $[M+2H]^{2+}$; found: 1078.36, 1617.16.

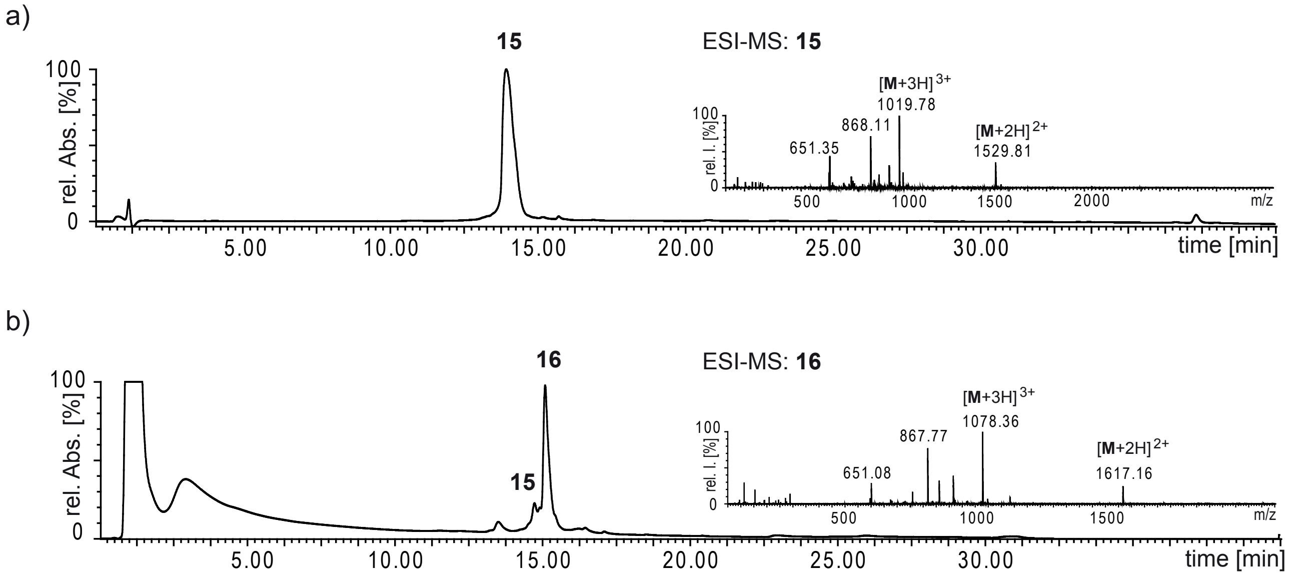

Figure S22: RP-HPLC-MS of a) digalactosylated glycopeptide hydrazide 1-25 **15** after gel filtration and b) crude reaction mixture after 2 d of the galacturonylation of **15** using GFP-B3GlcAT1 and UDP-GlcA.

6. Recombinant Expression of Linkage Region Glycosyltransferases

Plasmids for the expression of the glycosyltransferases (see below) were ordered from GenScript (Rijswijk, Netherlands).

General Protocols:

Transformation of *E. coli* strains: BL21 (DE3), Shuffle T7, Actic Express, Origami2 (DE3)
Chemically competent *E. coli* cells (20 µL) were mixed with a solution of the plasmid (1 µL, 0.2 µg of plasmid/µL in H_2O) and placed on ice for 30 min. After a heat shock (42 °C, 45 s) the cells were placed on ice for 1 min. Subsequently 1 mL of LB-medium was added and the culture was incubated in a thermo shaker (37 °C, 1.5 h, 950 rpm). The cells were pelleted (6000 g, 2 min, 4 °C), 900µL of the supernatant were removed and the cells were resuspended in the remaining volume. The culture was plated out on an agar plate with the required antibiotic and incubated at 37 °C. After 24 h colonies were picked and cultivated in LB medium.

For preparative expressions LB-medium (100mL) containing the appropriate antibiotic was inoculated with the transformed *E. coli* strain and incubated (37 °C, 16h, 175 rpm). Subsequently, the overnight culture was added to LB-medium (2.5-5 L) containing antibiotic resulting in an initial OD_{600} of 0.1. The culture was incubated (37 °C, 110-130 rpm) up to an OD_{600} of 0.4 followed by lowering the temperature to the desired value for the overexpression. Recombinant expression was started by addition of IPTG (final concentration: 0.4 mM, stock solution 1 M) at an OD_{600} of 0.6. After 48 h the cells were harvested (8000 g, 4 °C, 8 min) and stored at -20 °C until use.

Lysis of bacterial pellets:
The bacterial pellets were placed on ice and resuspended in cold Ni^{2+}-IMAC binding buffer (10 mL/g of bacterial pellet) under stirring. Lysis was carried out in rosetta shaped glass flasks using a Bandelin *SONOPLUS* HD2200 ultrasound homogenizer (12 x 20 s, 1 min pause after each cycle, power: 60 %, cycle: 50 %) at 0° C.

Sequence of the proteins:
The plasmid for the expression of **17** (pET11a*His6-B3GlcAT1*) was designed according to Pedersen.[16] The following protein sequence was expressed:

His6-B3GlcAT1 **17**: (pET11a*His6-B3GlcAT1*)
MGSSHHHHHH SSGLVPRGSH MTIYVVTPTY ARLVQKAELV RLSQTLSLVP

RLHWLLVEDA EGPTPLVSGL LAASGLLFTH LVVLTPKAQR LREGEPGWVH PRGVEQRNKA LDWLRGRGGA VGGEKDPPPP GTQGVVYFAD DDNTYSRELF EEMRWTRGVS VWPVGLVGGL RFEGPQVQDG RVVGFHTAWE PSRPFPVDMA GFAVALPLLL DKPNAQFDST APRGHLESSL LSHLVDPKDL EPRAANCTRV LVWHTRTEKP KMKQEEQLQR QGRGSDPAIE V

the underlined sequence corresponds to:

AA 76-335 of human B3GlcAT1 (Uniprot entry: O94766 · B3GA3_HUMAN).

His6SUMO-B3GlcAT1 **17S**: (pET11a*His6-SUMO-B3GlcAT1*)

GSSHHHHHH**G SGLVPRGSAS MSDSEVNQEA KPEVKPEVKP ETHINLKVSD GSSEIFFKIK KTTPLRRLME AFAKRQGKEM DSLRFLYDGI RIQADQTPED LDMEDNDIIE AHREQIGG**EA LPTIYVVTPT YARLVQKAEL VRLSQTLSLV PRLHWLLVED AEGPTPLVSG LLAASGLLFT HLVVLTPKAQ RLREGEPGWV HPRGVEQRNK ALDWLRGRGG AVGGEKDPPP PGTQGVVYFA DDDNTYSREL FEEMRWTRGV SVWPVGLVGG LRFEGPQVQD GRVVGFHTAW EPSRPFPVDM AGFAVALPLL LDKPNAQFDS TAPRGHLESS LLSHLVDPKD LEPRAANCTR VLVWHTRTEK PKMKQEEQLQ RQGRGSDPAI EV

the underlined sequence corresponds to AA 72-335 of human B3GlcAT1 (Uniprot entry: O94766 · B3GA3_HUMAN); in bold SUMO.

His6SUMO-B4GalT7 **18S**: (pET11a*His6-SUMO-B4GalT7*)

GSSHHHHHH**G SGLVPRGSAS MSDSEVNQEA KPEVKPEVKP ETHINLKVSD GSSEIFFKIK KTTPLRRLME AFAKRQGKEM DSLRFLYDGI RIQADQTPED LDMEDNDIIE AHREQIGG**EH WEEDASWGPH RLAVLVPFRE RFEELLVFVP HMRRFLSRKK IRHHIYVLNQ VDHFRFNRAA LINVGFLESS NSTDYIAMHD VDLLPLNEEL DYGFPEAGPF HVASPELHPL YHYKTYVGGI LLLSKQHYRL CNGMSNRFWG WGREDDEFYR RIKGAGLQLF RPSGITTGYK TFRHLHDPAW RKRDQKRIAA QKQEQFKVDR EGGLNTVKYH VASRTALSVG GAPCTVLNIM LDCDKTATPW CTFS

the underlined sequence corresponds to AA 82-327 of human B4GalT7 (Uniprot entry: Q9UBV7 · B4GT7_HUMAN); in bold SUMO.

His6SUMO-B3GalT6 **19S**: (pET11a*His6-SUMO-B3GalT6*)

GSSHHHHHH**G SGLVPRGSAS MSDSEVNQEA KPEVKPEVKP ETHINLKVSD GSSEIFFKIK KTTPLRRLME AFAKRQGKEM DSLRFLYDGI RIQADQTPED LDMEDNDIIE AHREQIGG**PG DPRAMSGRSP PPPAPARAAA FLAVLVASAP

RAAERRSVIR STWLARRGAP GDVWARFAVG TAGLGAEERR ALEREQARHG
DLLLLPALRD AYENLTAKVL AMLAWLDEHV AFEFVLKADD DSFARLDALL
AELRAREPAR RRRLYWGFFS GRGRVKPGGR WREAAWQLCD YYLPYALGGG
YVLSADLVHY LRLSRDYLRA WHSEDVSLGA WLAPVDVQRE HDPRFDTEYR
SRGCSNQYLV THKQSLEDML EKHATLAREG RLCKREVQLR LSYVYDWSAP
PSQCCQRREG IP

the underlined sequence corresponds to AA 35-329 of human B3GalT6 (Uniprot entry: Q96L58 · B3GT6_HUMAN); in bold SUMO.

His6SUMO-B3GalT6(P35S) **20S**: (pET11a*His6-SUMO-B3GalT6(P35S)*)
GSSHHHHHH**G SGLVPRGSAS MSDSEVNQEA KPEVKPEVKP ETHINLKVSD**
GSSEIFFKIK KTTPLRRLME AFAKRQGKEM DSLRFLYDGI RIQADQTPED
LDMEDNDIIE AHREQIGGSG DPRAMSGRSP PPPAPARAAA FLAVLVASAP
RAAERRSVIR STWLARRGAP GDVWARFAVG TAGLGAEERR ALEREQARHG
DLLLLPALRD AYENLTAKVL AMLAWLDEHV AFEFVLKADD DSFARLDALL
AELRAREPAR RRRLYWGFFS GRGRVKPGGR WREAAWQLCD YYLPYALGGG
YVLSADLVHY LRLSRDYLRA WHSEDVSLGA WLAPVDVQRE HDPRFDTEYR
SRGCSNQYLV THKQSLEDML EKHATLAREG RLCKREVQLR LSYVYDWSAP
PSQCCQRREG IP

the underlined sequence corresponds to AA 35-329 of human B3GalT6 (Uniprot entry: Q96L58 · B3GT6_HUMAN); in red mutations, in bold SUMO.

His6SUMO-B3GalT6(P35S)(C206S) **21S**: (pET11a*His6-SUMO-B3GalT6(P35S)(C206S)*)
GSSHHHHHH**G SGLVPRGSAS MSDSEVNQEA KPEVKPEVKP ETHINLKVSD**
GSSEIFFKIK KTTPLRRLME AFAKRQGKEM DSLRFLYDGI RIQADQTPED
LDMEDNDIIE AHREQIGGSG DPRAMSGRSP PPPAPARAAA FLAVLVASAP
RAAERRSVIR STWLARRGAP GDVWARFAVG TAGLGAEERR ALEREQARHG
DLLLLPALRD AYENLTAKVL AMLAWLDEHV AFEFVLKADD DSFARLDALL
AELRAREPAR RRRLYWGFFS GRGRVKPGGR WREAAWQLSD YYLPYALGGG
YVLSADLVHY LRLSRDYLRA WHSEDVSLGA WLAPVDVQRE HDPRFDTEYR
SRGCSNQYLV THKQSLEDML EKHATLAREG RLCKREVQLR LSYVYDWSAP
PSQCCQRREG IP

the underlined sequence corresponds to AA 35-329 of human B3GalT6 (Uniprot entry: Q96L58 · B3GT6_HUMAN); in red mutations, in bold SUMO.

6.1 Expression and purification of B3GlcAT-I 17H

Ni^{2+}-IMAC binding buffer: 750 mM NaCl, 20 mM NaH_2PO_4, 20 mM imidazole, 0.2 mM DTT, pH 8.0
Ni^{2+}-IMAC washing buffer: 750 mM NaCl, 20 mM NaH_2PO_4, 75 mM imidazole, 0.2 mM DTT, pH 8.0
Ni^{2+}-IMAC elution buffer: 750 mM NaCl, 20 mM NaH_2PO_4, 250 mM imidazole, 0.2 mM DTT, pH 8.0
dialysis buffer: 1 M NaCl, 20 mM MES, pH 6.8

Overexpression of **17H** was conducted in 3.7 L of medium for 48 h in the following *E. coli* strains at 16°C (BL21 (DE3), BL21, SHuffle T7 and Origami2) and at 13° C (Arctic express) as described above. The bacterial pellets were lysed and the suspensions were cleared by centrifugation (40000g, 4° C, 45min). The supernatant was filtered (0.45 µm, syringe filter, PVDF, Roth, Germany) and loaded onto a Ni^{2+}-IMAC-column containing approx. 10 mL of NTA-gel. After loading the sample (2 mL/min) the column was washed with 150 mL of Ni^{2+}-IMAC binding buffer followed by Ni^{2+}-IMAC washing buffer (75 mL) and Ni^{2+}-IMAC elution buffer (50 mL, 7 mL/min). The main fraction was dialyzed (3x330 mL of dialysis buffer) changing the buffer every 24 h.
Yields:
1.4 mg **17H**/L of *E. coli* BL21 (DE3) culture
3.4 mg **17H**/L of *E. coli* Arctic Express culture:
4.2 mg **17H**/L of *E. coli* Origami2 (DE3) culture
2.4 mg **17H**/L of *E. coli* SHuffle T7 culture

LC-MS (Discovery BIO Wide Pore C5, 50 x 2.1mm, 20-70 % MeCN/H_2O + 0.1 % HCOOH). ESI-MS of **17H**: *m/z* (average isotopes) $C_{1385}H_{2168}N_{406}O_{395}S_5$ (30987.23), calculated: 1631.91 $[M+19H]^{19+}$, 1823.78 $[M+17H]^{17+}$; 2066.81 $[M+15H]^{15+}$; found: 1631.66, 1823.76, 2066.61; deconvoluted mass: 30984.

Purification of **17H** by gel filtration:

The protein **17H** obtained after Ni^{2+}-IMAC purification of the expression in *E. coli* Origami2 (DE3) was concentrated in a 50 mL Amicon stirred cell (membrane: NADIR UP010 P) to approx. 6 mL. After addition of 50 mL of buffer (1 M NaCl, 20 mM MES, pH 6.7) the solution was concentrated to

6 mL, centrifuged (25000 g, 4 °C, 2 min) and purified by gel filtration (2 x 3 mL, Superdex 200 pg 16/600, 0.8 ml/min, 1 M NaCl, 20 mM MES, pH 6.7). Yield of **17**: 5.4 mg (35 %).

Concentration of the fraction obtained from gel filtration in a 10mL Amicon stirred cell (membrane: NADIR UP010 P) was accompanied by loss of protein. Yield of **17H**: 1.5 mg.

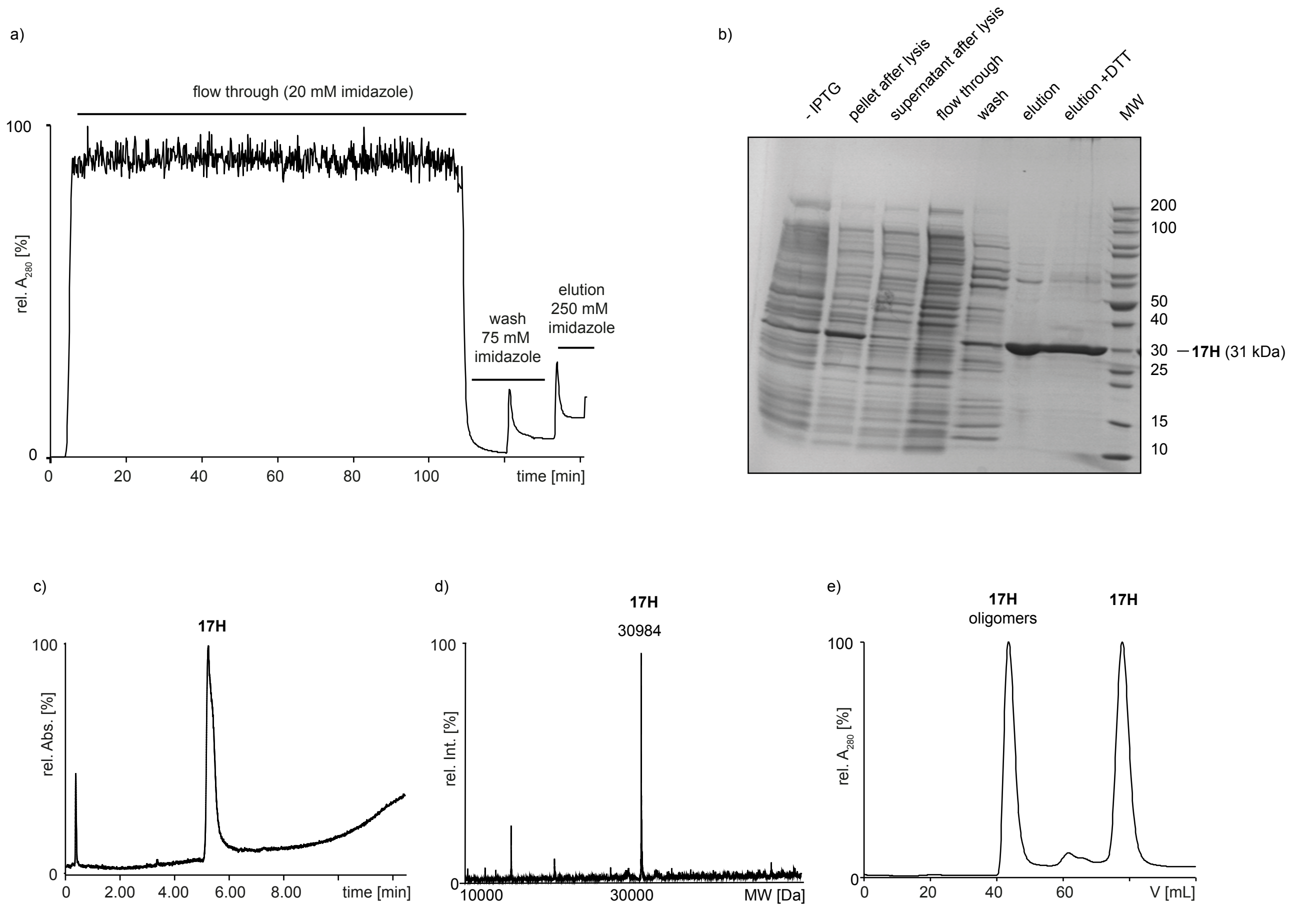

Figure S23: a) Ni^{2+}-IMAC of **17**; b) SDS-PAGE of Ni^{2+}-IMAC purification of **17H**; c) RP-HPLC-MS of **17H**; d) deconvoluted MS of **17H**; e) SEC of purified **17H**.

6.2 Expression and purification of SUMO-B3GlcAT-I 17S

Ni^{2+}-IMAC binding buffer: 750 mM NaCl, 20 mM NaH_2PO_4, 20 mM imidazole, pH 8.0
Ni^{2+}-IMAC washing buffer: 750 mM NaCl, 20 mM NaH_2PO_4, 75 mM imidazole, pH 8.0
Ni^{2+}-IMAC elution buffer: 750 mM NaCl, 20 mM NaH_2PO_4, 250 mM imidazole, pH 8.0
dialysis buffer: 0,5 M NaCl, 20 mM MES, pH 6.6

Overexpression of **17S** was conducted in 3.7 L of medium for 48 h in the following *E. coli* strains at 16°C (BL21 (DE3), BL21, SHuffle T7 and Origami2) and at 13° C (Arctic express) as described above. The bacterial pellets were lysed and the suspensions were cleared by centrifugation ((40000g,

4° C, 45min). The supernatant was filtered (0.45 µm, syringe filter, PVDF, Roth, Germany) and loaded onto a Ni^{2+}-IMAC-column containing approx. 10 mL of NTA-gel. After loading the sample (2 mL/min) the column was washed with 150 mL of Ni^{2+}-IMAC binding buffer followed by Ni^{2+}-IMAC washing buffer (75 mL) and Ni^{2+}-IMAC elution buffer (50 mL, 7 mL/min). The main fraction was dialyzed (3x500 mL of dialysis buffer) changing the buffer every 24 h.

Yields:

8.5 mg **17S**/L of *E. coli* BL21 (DE3) culture

10.5 mg **17S**/L of *E. coli* Arctic Express culture:

18.5 mg **17S**/L of *E. coli* Origami2 (DE3) culture

8 mg **17S**/L of *E. coli* SHuffle T7 culture

LC-MS (Discovery BIO Wide Pore C5, 50 x 2.1mm, 10-70 % MeCN/H_2O + 0.1 % HCOOH). ESI-MS of **17S**: *m/z* (average isotopes) $C_{1886}H_{2975}N_{545}O_{560}S_8$ (42501.28 Da), calculated: 989.40 $[M+43H]^{43+}$, 966.94 $[M+44H]^{44+}$, 945.47 $[M+45H]^{45+}$; found: 989.40 $[M+43H]^{43+}$, 966.93 $[M+44H]^{44+}$, 945.44 $[M+45H]^{45+}$; deconvoluted mass: 42501.0 Da.

2 mg of **17S** obtained after Ni^{2+}-IMAC were further purified by gel filtration (2 x 3 mL of sample, Superdex 200 pg 16/600, 0.8 ml/min, 1 M NaCl, 20 mM MES, pH 6.7).

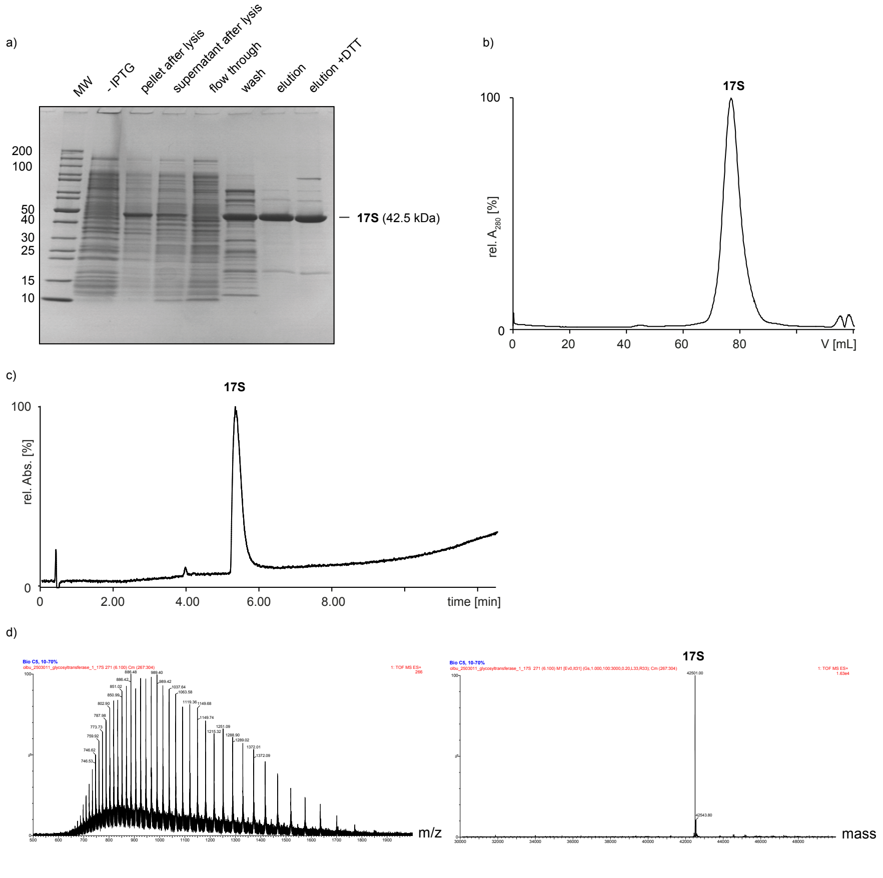

Figure S24: a) SDS-PAGE of Ni^{2+}-IMAC purification of **17S**; b) SEC of purified **17S**; c) RP-LC-MS of **17S**; d) ESI-MS and deconvoluted MS of **17S**.

6.3 Expression and purification of SUMO-B4GalT7 18S

Ni^{2+}-IMAC binding buffer: 300 mM NaCl, 30 mM NaH_2PO_4, 20 mM imidazole, pH 7.0
Ni^{2+}-IMAC washing buffer: 300 mM NaCl, 30 mM NaH_2PO_4, 110 mM imidazole, pH 7.0
Ni^{2+}-IMAC elution buffer: 300 mM NaCl, 30 mM NaH_2PO_4, 250 mM imidazole, pH 7.0
dialysis buffer: 0.5 M L-Arg, 0,15 M NaCl, 30 mM HEPES, pH 7.0

Overexpression of **18S** was conducted in 3.7 L of medium for 48 h in *E. coli* Arctic express at 13° C as described above. The bacterial pellets were lysed and the suspensions were cleared by centrifugation (40000g, 4° C, 45min). The supernatant was filtered (0.45 µm, syringe filter, PVDF, Roth, Germany) and loaded onto a Ni^{2+}-IMAC-column containing approx. 10 mL of NTA-gel. After loading the sample (2 mL/min) the column was washed with 150 mL of Ni^{2+}-IMAC binding buffer followed by Ni^{2+}-IMAC washing buffer (75 mL) and Ni^{2+}-IMAC elution buffer (50 mL, 7 mL/min). The main fraction was dialyzed (3x600 mL of dialysis buffer) changing the buffer every 24 h.

Yield: 5 mg **18S**/L of *E. coli* Arctic Express culture:

1.2 mg of **18S** obtained after Ni^{2+}-IMAC were further purified by gel filtration (1 mL of sample, Superdex 200 pg 16/600, 1 ml/min, 0.5 M L-Arg, 0,15 M NaCl, 30 mM HEPES, pH 7.0).

The protein samples in buffer with L-Arg were prepared for SDS-PAGE by TCA (20 %) precipitation.

LC-MS (Discovery BIO Wide Pore C5, 50 x 2.1mm, 10-70 % MeCN/H_2O + 0.1 % HCOOH). ESI-MS of **18S**: *m/z* (average isotopes) $C_{1873}H_{2881}N_{543}O_{536}S_{12}$ (41966.64 Da), calculated: 840.33 $[M+50H]^{50+}$, 823.88 $[M+51H]^{51+}$, 808.05 $[M+52H]^{52+}$; found: 840.32 $[M+50H]^{50+}$, 823.82 $[M+51H]^{51+}$, 807.99 $[M+52H]^{52+}$; deconvoluted mass: 41966.00 Da.

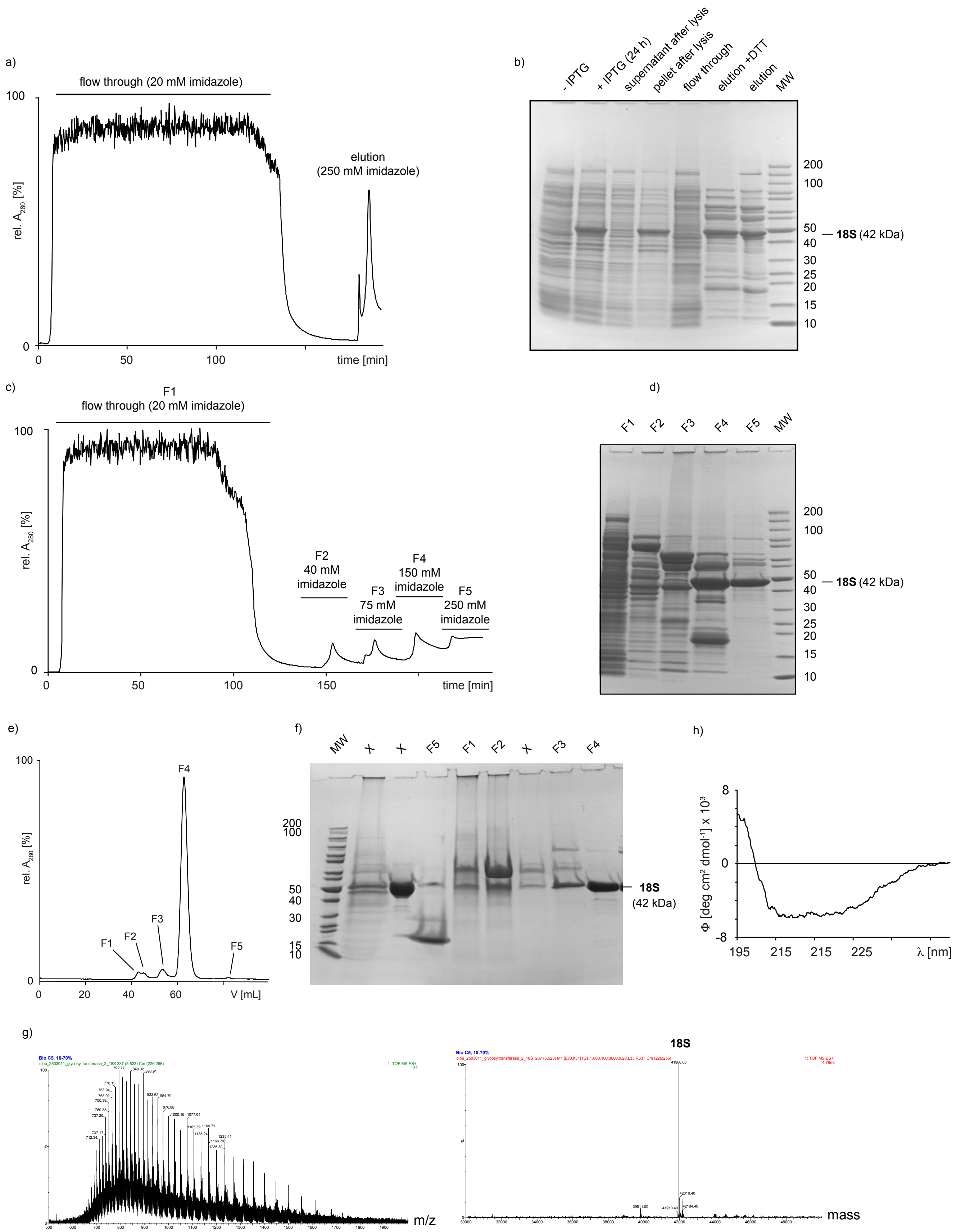

Figure S25: a) Ni^{2+}-IMAC of **18S** without washing buffer step; b) SDS-PAGE of Ni^{2+}-IMAC purification of **18S**; c) Ni^{2+}-IMAC of **18S** with sequential washing buffers containing 40 mM, 75 mM and 150 mM imidazole; d) SDS-PAGE of Ni^{2+}-IMAC purification of **18S** shown in c): e) SEC of purified **18S**; f) SDS-PAGE of SEC purification of **18S** shown in e) X = samples from another experiment; g) ESI and deconvoluted MS of **18S**.; h) CD spectrum of **18S**.

6.4 Expression and purification of SUMO-B3GalT6 19S, 20S, 21S

Ni^{2+}-IMAC binding buffer: 750 mM NaCl, 20 mM NaH_2PO_4, 20 mM imidazole, pH 7.0
Ni^{2+}-IMAC washing buffer: 750 mM NaCl, 20 mM NaH_2PO_4, 110 mM imidazole, pH 7.0
Ni^{2+}-IMAC elution buffer: 750 mM NaCl, 20 mM NaH_2PO_4, 250 mM imidazole, pH 7.0
dialysis buffer: 1 M NaCl, 20 mM MES, pH 6.7

Overexpression of **19S** was conducted in 3.7 L of medium for 48 h in the following *E. coli* strains at 16°C (BL21 (DE3), BL21, SHuffle T7 and Origami2) and at 13° C (Arctic express) as described above. The bacterial pellets were lysed and the suspensions were cleared by centrifugation (40000g, 4° C, 45min). The supernatant was filtered (0.45 µm, syringe filter, PVDF, Roth, Germany) and loaded onto a Ni^{2+}-IMAC-column containing approx. 10 mL of NTA-gel. After loading the sample (3 mL/min) the column was washed with 150 mL of Ni^{2+}-IMAC binding buffer followed by Ni^{2+}-IMAC washing buffer (75 mL) and Ni^{2+}-IMAC elution buffer (50 mL). The main fraction was dialyzed at 4° C (3x500 mL of dialysis buffer) changing the buffer every 24 h.
Yields:
< 0.1 mg **19S**/L of *E. coli* BL21 (DE3) culture
< 0.1 mg **19S**/L of *E. coli* Arctic Express culture:
1.4 mg **19S**/L of *E. coli* Origami2 (DE3) culture
12 mg **19S**/L of *E. coli* SHuffle T7 culture

20S: Ni^{2+}-IMAC binding buffer: 750 mM NaCl, 20 mM NaH_2PO_4, 20 mM imidazole, pH 7.0
Ni^{2+}-IMAC washing buffer: 750 mM NaCl, 20 mM NaH_2PO_4, 100 mM imidazole, pH 7.0
Ni^{2+}-IMAC elution buffer: 750 mM NaCl, 20 mM NaH_2PO_4, 250 mM imidazole, pH 7.0
dialysis buffer: 1 M NaCl, 20 mM MES, pH 6.7

Overexpression of **20S** was conducted in 3.7 L of medium for 48 h in *E. coli* SHuffle T7 at 16°C as described above. The bacterial pellet was lysed and the suspension was cleared by centrifugation (40000g, 4° C, 45min). The supernatant was filtered (0.45 µm, syringe filter, PVDF, Roth, Germany) and loaded onto a Ni^{2+}-IMAC-column containing approx. 10 mL of NTA-gel. After loading the sample (3 mL/min) the column was washed with 150 mL of Ni^{2+}-IMAC binding buffer followed by Ni^{2+}-IMAC washing buffer (75 mL) and Ni^{2+}-IMAC elution buffer (50 mL). The main fraction was dialyzed at 4° C (3x500 mL of dialysis buffer) changing the buffer every 24 h.
Yield: 15 mg of **20S**/L of *E. coli* SHuffle T7 culture

ESI-MS of **20S**: *m/z* (average isotopes) $C_{2053}H_{3218}N_{622}O_{599}S_{112}$ (46582.66 Da), calculated: 1864.31 $[M+25H]^{25+}$, 1726.28 $[M+27H]^{27+}$; found: 1864.62, 1726.61; deconvoluted mass: 46586.

21S: Ni^{2+}-IMAC binding buffer: 750 mM NaCl, 20 mM NaH_2PO_4, 20 mM imidazole, pH 7.0
Ni^{2+}-IMAC elution buffer: 750 mM NaCl, 20 mM NaH_2PO_4, 250 mM imidazole, pH 7.0
gel filtration buffer: 1 M NaCl, 20 mM MES, pH 6.7

Overexpression of **21S** was conducted in 1 L of medium for 48 h in *E. coli* SHuffle T7 at 16°C as described above. The bacterial pellet was lysed and the suspension was cleared by centrifugation (40000g, 4° C, 45min). The supernatant was filtered (0.45 µm, syringe filter, PVDF, Roth, Germany) and loaded onto a Ni^{2+}-IMAC-column containing approx. 10 mL of NTA-gel. After loading the sample (3 mL/min) the column was washed with 250 mL of Ni^{2+}-IMAC binding buffer followed by Ni^{2+}-IMAC elution buffer (35 mL). The main fraction was concentrated in a 50 mL Amicon stirred cell (membrane: NADIR UP010 P) to approx. 10 mL. The concentrated solution of **21S** was further purified by gel filtration (1 mL of sample, Superdex 200 increase 10/300, 0.5 ml/min, 1 M NaCl, 20 mM MES, pH 6.7).
Yield: 10.2 mg **21S**/L of *E. coli* SHuffle T7 culture

LC-MS (Discovery BIO Wide Pore C5, 50 x 2.1mm, 10-70 % MeCN/H_2O + 0.1 % HCOOH). ESI-MS of **21S**: *m/z* (average isotopes) $C_{2053}H_{3218}N_{622}O_{600}S_{11}$ (46566.74 Da), calculated: 832.55 $[M+56H]^{56+}$, 817.96 $[M+51H]^{57+}$, 803.87 $[M+58H]^{58+}$; found: 832.57 $[M+56H]^{56+}$, 817.90 $[M+51H]^{57+}$, 803.81 $[M+58H]^{58+}$; deconvoluted mass: 46565.0 Da.

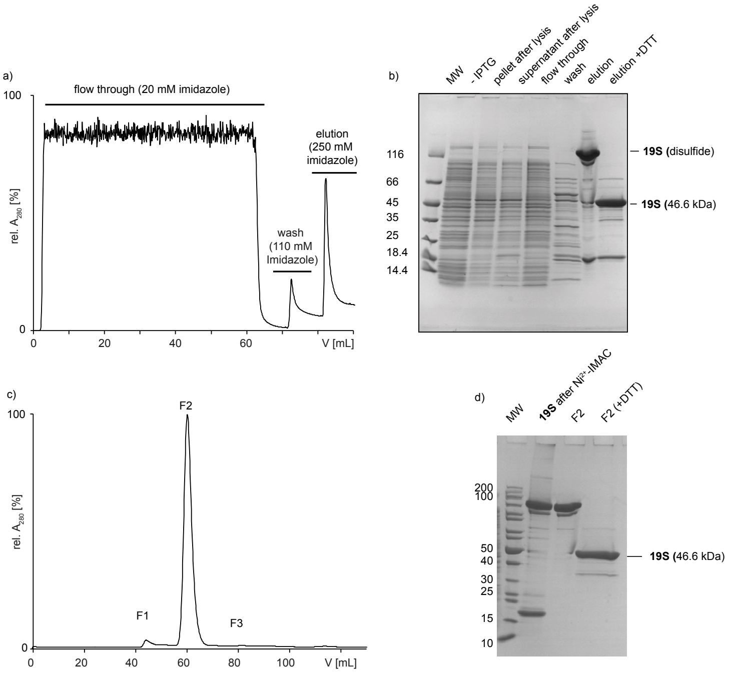

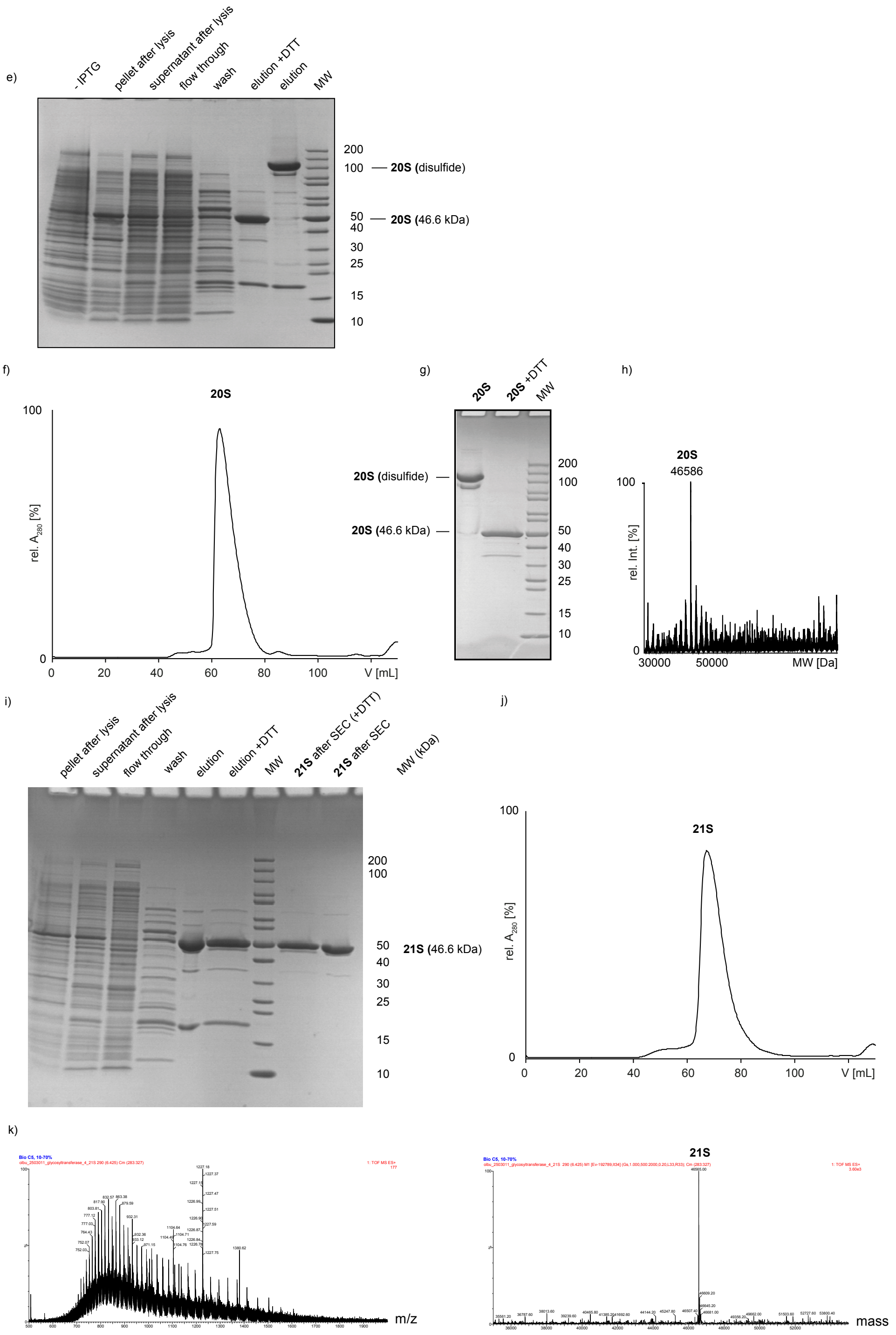

Figure S26: a) Ni^{2+}-IMAC of **19S** without washing buffer step; b) SDS-PAGE of Ni^{2+}-IMAC purification of **19S**; c) SEC of **19S** after Ni^{2+}-IMAC; d) SDS-PAGE of **19S** after SEC purification; e) SDS-PAGE of Ni^{2+}-IMAC purification of **20S** f) SEC of **20S** purified by Ni^{2+}-IMAC; g) SDS-PAGE of **20S** after SEC purification shown in f); h) deconvoluted ESI-MS of **20S**; i) SDS-PAGE of **21S** after Ni^{2+}-IMAC and SEC; j) SEC of **21S** after Ni^{2+}-IMAC; k) ESI-MS and deconvoluted mass of **21S**.

6.5 Cleavage of the SUMO tag from 17S, 18S, 20S, 21S

Proteins in storage buffers:

17S (2 mg/mL) in 50 mm NaH_2PO_4, 0.5 m NaCl, pH 6.6,

18S (1.7 mg/mL) in 50 mm NaH_2PO_4, 0.5 m NaCl, pH 6.6,

20S (1.7 mg/mL) in 20 mm Mes, 1 m NaCl, pH 6.7,

21S (1.4 mg/mL) in 20 mm Mes, 1 m NaCl, pH 6.7,

His6-SENP2 (2.7 mg/mL in 5 mm TRIS, 75 mm NaCl, 2 mm DTT, 0.1 mm EDTA, 10 % glycerine, pH 6.8)

For cleavage of the His_6-SUMO tag, 200 µg of the glycosyltransferases **17S**, **18S**, **20S** or **21S** in their respective storage buffers were incubated with 2 µL of the SENP2 stock solution followed by additional 4 µL of SENP2 after 4 h. DeSUMOylation was complete in all cases after 8 h at ambient temperature (21° C).

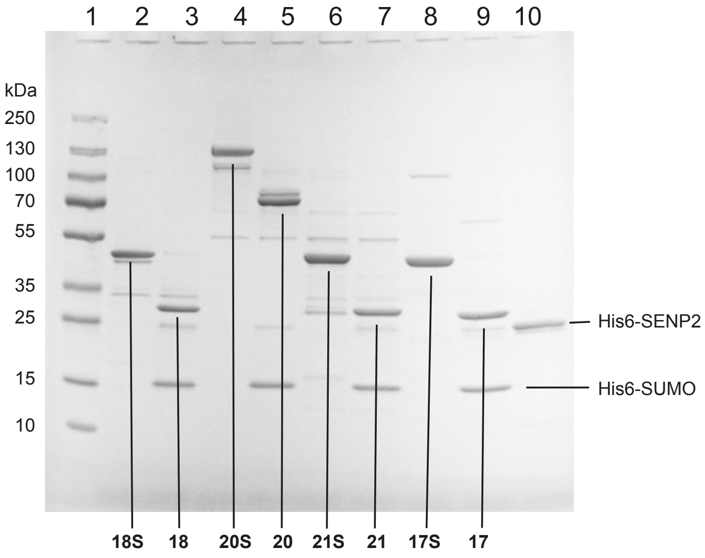

1) MW-Standard
2) His6-SUMO-B4GalT7 **18S** (42.0 kDa)
3) deSUMOylation of **18S** to B4GalT7 **18** (28.7 kDa) after 16h
4) His6-SUMO-B3GalT6 **20S** (93.2 kDa)
5) deSUMOylation of **20S** to B3GalT6 **20** (66.6 kDa) after 16h
6) His6-SUMO-B3GalT6 **21S** (46.6 kDa)
7) deSUMOylation of **21S** to B3GalT6 **21** (33.3 kDa) after 16h
8) His6-SUMO-B3GlcAT1 **17S** (42.2 kDa)
9) deSUMOylation of **17S** toB3GlcAT1 **17** (29.7 kDa) after 16h
10) His6-SENP2

Figure S27: SDS-PAGE of deSUMOylation of **17S**, **18S**, **20S** and **21S**.

6.6 SEC-MALS of 17S, 18S, 20S, 21S and 17, 18, 20, 21

SEC buffer: 0.5 M NaCl, 20 mM Mes, pH 6.7

The samples of SUMOylated glycosyltransferases (**17S**, **18S**, **20S**, **21S**) and of the desumoylation reaction shown above (containing transferases **17**, **18**, **20**, **21**) were diluted to a concentration of 1 mg/mL with the SEC buffer and centrifuged. The samples were subsequently characterized by SEC-MALS (size-exclusion chromatography – multi-angle light scattering) using a Superdex 200 Increase 10/300 GL column (50 µL of sample, 0.75 mL/min, 0.5 M NaCl, 20 mM Mes, pH 6.7) connected to an ÄKTApure FPLC system (GE Healthcare Life Sciences) and coupled to an Optilab refractometer with a mini-DAWN detector (Wyatt Technology).

Table S5: molecular weights (nominal mass) for **17S**, **18S**, **20S**, **21S** and **17**, **18**, **20**, **21**

glycosyltransferase	molecular weight (kDa)
**17S** SUMO-B3GlcAT1	42.5
**17** B3GlcAT1	29.2
**18S** SUMO-B4GalT7	42.0
**18** B4GalT7	28.7
**20S** SUMO-B3GalT6	93.2
**20** B3GalT6	66.6
**21S** SUMO-B3GalT6 (C206S)	46.6
**21** B3GalT6 (C206S)	33.3

After purification by SEC an analytical sample of **20** was analyzed by LC-MS (FortisBIO C4, 1.7µm, 30 x 2.1mm, 20-70 % MeCN/H_2O + 0.1 % HCOOH). ESI-MS of **20**: *m/z* (average isotopes) $C_{2962}H_{4618}N_{900}O_{830}S_{16}$ (66629.60), calculated: 2150.34 $[M+31H]^{31+}$; found: 2150.43; deconvoluted mass: 66617.

After purification by SEC an analytical sample of **20** was reduced (DTT) and analyzed by LC-MS (FortisBIO C4, 1.7µm, 30 x 2.1mm, 20-70 % MeCN/H_2O + 0.1 % HCOOH). ESI-MS of $\mathbf{20^{red}}$: *m/z* (average isotopes) $C_{1481}H_{2310}N_{450}O_{415}S_8$ (33315.88), calculated: 2222.06 $[M+15H]^{15+}$; found: 2221.45; deconvoluted mass: 33313.

After purification by SEC an analytical sample of **21** was analyzed by LC-MS (FortisBIO C4, 1.7µm, 30 x 2.1mm, 20-70 % MeCN/H_2O + 0.1 % HCOOH). ESI-MS of **21**: *m/z* (average isotopes) $C_{1481}H_{2310}N_{450}O_{416}S_7$ (33299.82), calculated: 2220.99 $[M+15H]^{15+}$; found: 2222.36; deconvoluted mass: 33322.

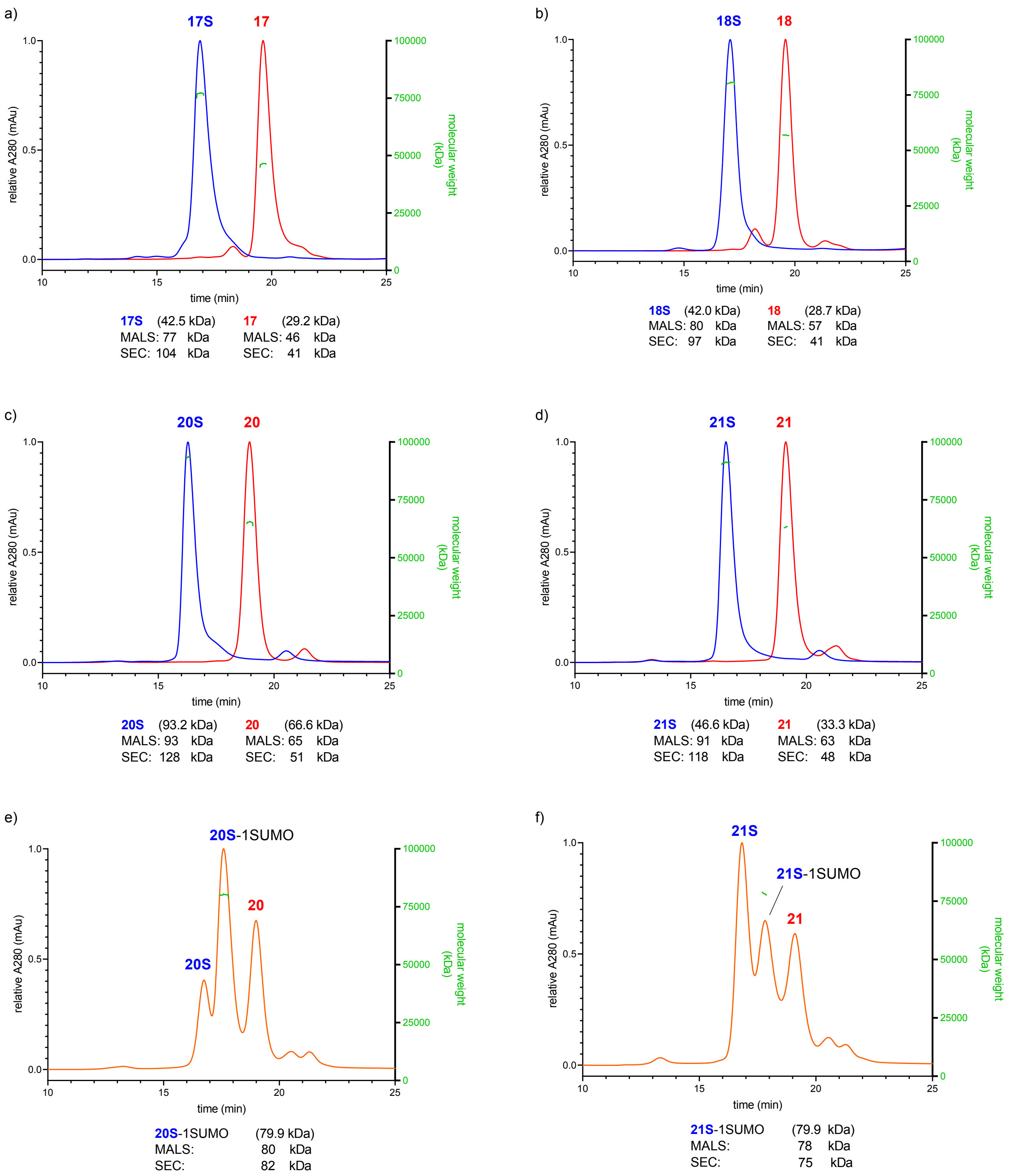

Figure S28: overlay of SEC-MALS data (nominal mass in parentheses) of a) B3GlcAT-1 **17S**, **17**; b) B4GalT7 **18S**, **18**; c) B3GalT6 **20S**, **20**, d) B3GalT6 (C206S) **21S**, **21**; e) formation of intermediate **20S**-1SUMO after 15 min; f) formation of intermediate **21S**-1SUMO after 15 min.

7. Enzymatic Glycosylation of Glycopeptides and N-Glycans

7.1 Enzymatic Glycosylation of Bikunin 1-25 Glycopeptide Hydrazide A

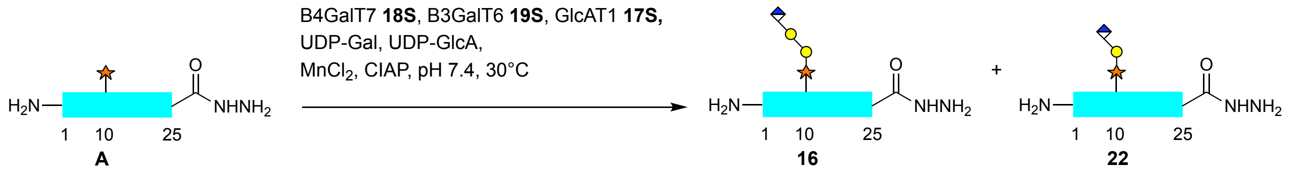

Figure S29: Enzymatic elongation of the xylosyl residue of **A** using glycosyltransferases **17S**, **18S**, **19S** and **17S** gives the expected product **16** accompanied by a side product with a truncated glycan (**22**).

Buffers and solutions:

Reaction buffer: 200 mM MOPS, 0.5 M NaCl, pH 7

SUMO-B4GalT7 **18S** (1.7 mg/mL) in 50 mM $NaH_2PO_4 \cdot 2\ H_2O$, 0.5 M NaCl, pH 6.6

SUMO-B3GalT6 **20S** (1.72 mg/mL) in 20 mM Mes, 1 M NaCl, pH 6.7

SUMO-B3GlcAT1 **17S** (2 mg/mL) in 50 mM $NaH_2PO_4 \cdot 2\ H_2O$, 0.5 M NaCl, pH 6.6

Stock solution (40 µL total volume): 7.9 µL of reaction buffer (200 mM MOPS, 0.5 M NaCl, pH 7), 7.1 µL of **18S** (1.7 mg/mL), 7.0 µL of **20S** (1.72 mg/mL), 6.0 µL of **17S** (2 mg/mL), 4.9 µL of UDP-Gal (100 mg/mL in H_2O), 2.6 µL of UDP-GlcA (100 mg/mL in H_2O), 2.0 µL of BSA (20 mg/mL in reaction buffer), 2.0 µL of alkaline phosphatase (200 mU/µL in reaction buffer) and 0.6 µL $MnCl_2$ (10 mg/mL in reaction buffer) were combined and vortexed prior to use.

Glycopeptide hydrazide **A** (0.4 mg, 146.3 nmol) was dissolved in 29.3 µL of stock solution and incubated at 30 °C for 24h. The resulting reaction conditions were: **A** (5 mM), **18S** (0.3 mg/mL), **20S** (0.3 mg /mL), **17S** (0.3 mg /mL), UDP-Gal (20 mM), UDP-GlcA (10 mM), BSA (1 mg/mL), $MnCl_2$ 1 mM, alkaline phosphatase (10 mU/µL).

LC-MS (Hydrosphere C18 S-2 µm, 2.0 × 50 mm, 5-25% MeCN/H_2O +0.1% HCOOH).

ESI-MS of **16**: *m/z* (exact mass) $C_{131}H_{217}N_{31}O_{63}$ (3232.47), calculated: 1078.49 $[M+3H]^{3+}$, 809.12 $[M+4H]^{4+}$found: 1078.74, 809.31.

ESI-MS of **22**: *m/z* (exact mass) $C_{125}H_{207}N_{31}O_{58}$ (3070.42), calculated: 1024.47 $[M+3H]^{3+}$, 768.61 $[M+4H]^{4+}$found: 1024.76, 768.81.

ESI-MS of **15**: *m/z* (exact mass) $C_{125}H_{209}N_{31}O_{57}$ (3056.44), calculated: 1019.81 $[M+3H]^{3+}$, 765.11 $[M+4H]^{4+}$found: 1020.10, 765.32.

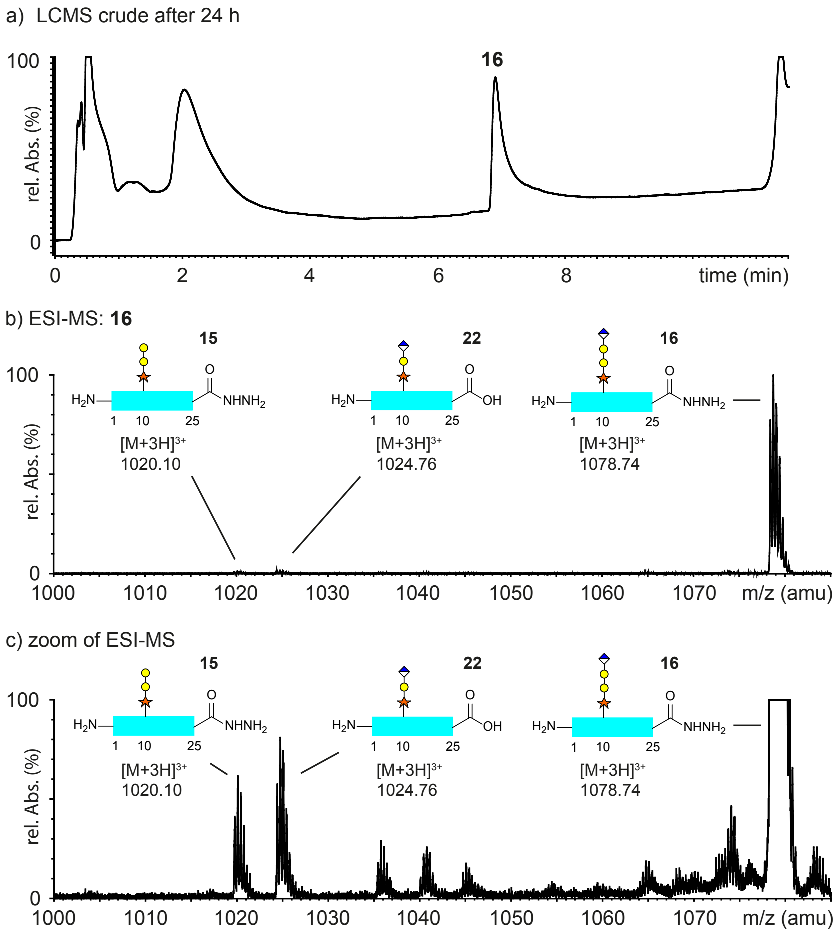

Figure S30: RP-HPLC-MS of a) crude reaction mixture of **16** after 24 and b) MS of the c) zoom in for the low intensity masses **15** and **16**.

7.2 Synthesis of Bikunin 1-50 Glycopeptide Hydrazide 23

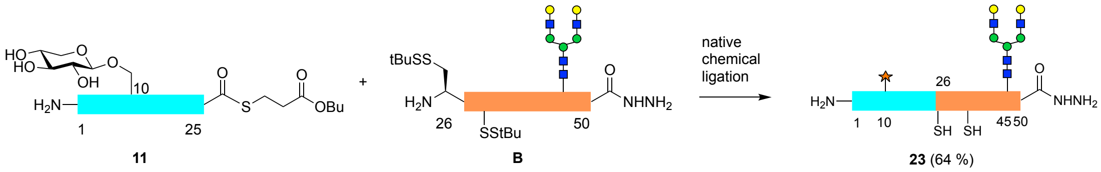

Figure S31: Synthesis of glycopeptide hydrazide 1-50 **23**.

Bikunin 1-25 glycopeptide thioester **11** (1.4 mg (0.5 µmol) and Bikunin 26-50 glycopeptide hydrazide **B** (1.8 mg, 0.43 µmol) were dissolved in ligation buffer (164 µL, 6 M GdmCl, 0.2 M NaH_2PO_4, 100 mM MPAA, 50 mM TCEP-HCl, pH 6.9) in an anaerobic chamber. After 64 h 2 µL of the reaction mixture were reduced with aqueous TCEP-HCl (18 µL, 50 mM pH 6-7) and analyzed by LC-MS (Hydrosphere C18, 2.0 × 30 mm, 10-35% MeCN/H_2O + 0.1% HCOOH). After complete conversion

of **B** the reaction mixture was treated with TCEP-HCl (2.35 mg, 8.2 µmol) and purified by gel filtration (Superdex Peptide 10/300 GL, 10 × 300 mm, 10% MeCN/H_2O +0.1% TFA, flow rate 0.2 mL/min). The fractions containing **23** were combined and lyophilized leaving a residue of 2.59 mg of crude **60**. The solid was dissolved ((900 µL of 10% MeCN/H_2O +0.1% TFA) and 100 µL (10 µmol) of 100 mM TCEP-HCl were added. After 15 min at room temperature the mixture was purified by RP-HPLC (Ascentis C18, 150 × 10 mm, 10-35% MeCN/H_2O +0.1% TFA, flow rate: 3 mL/min). The fractions containing **23** were combined and lyophilized. Yield: 1.8 mg of **23** (0.26 µmol, 64%) LC-MS of **23** (Hydrosphere C18 S-3 µm, 2.0 × 30 mm, 10-15% MeCN/H_2O +0.1% HCOOH). HR-MS of **23**: *m/z* (exact mass) $C_{292}H_{465}N_{65}O_{127}S_2$ (6978,14), calculated: 1164.0301 $[M+6H]^{6+}$, 1396.6346 $[M+5H]^{5+}$, 1745.5415 $[M+4H]^{4+}$; found: 1164.0295, 1396.6359, 1745.5419.

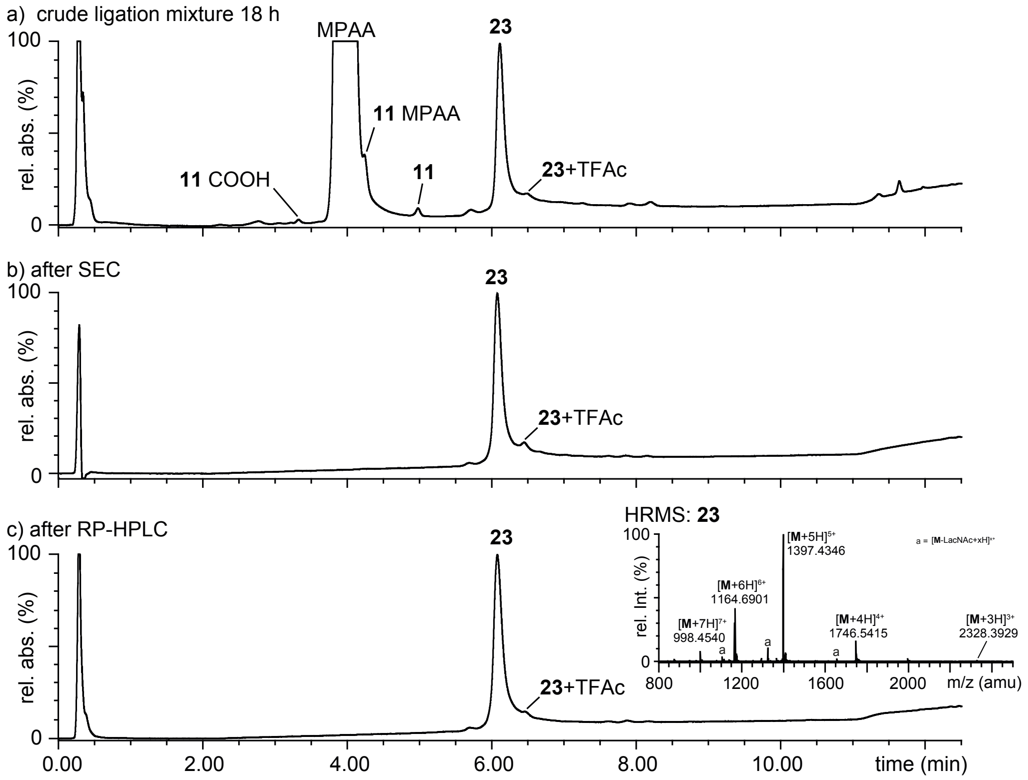

Figure S32: RP-HPLC-MS of glycopeptide hydrazide 1-50 **23**.: a) crude ligation mixture after 18 h; b) purified **23** after SEC; c) purified **23** after RP-HPLC

7.3 Enzymatic Glycosylation of Bikunin 1-50 Glycopeptide Hydrazide 23

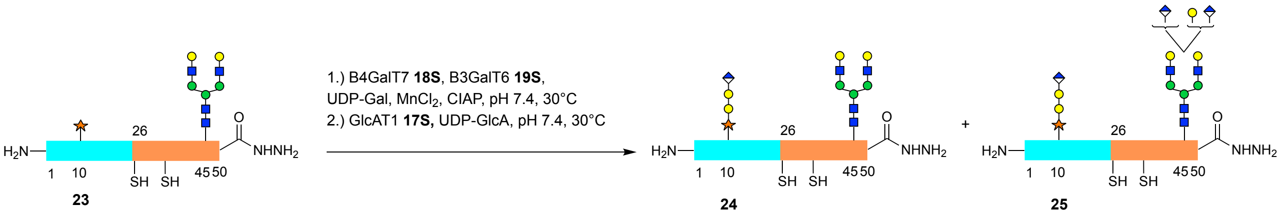

Figure S33: Enzymatic elongation of glycopeptide hydrazide 1-50 **23** using repeated additions of the galactosyltransferases **18S** and **19S** in the first step followed by glucuronyltransferase **17S** leads to side reactions in the N-glycan part.

Reaction buffer: 200 mM MOPS, 0.5 M NaCl, pH 7
SUMO-B4GalT7 **18S** (1.8 mg/mL) in 50 mM $NaH_2PO_4 \cdot 2\ H_2O$, 0.5 M NaCl, pH 6.6
SUMO-B3GalT6 **20S** (1.72 mg/mL) in 20 mM Mes, 1 M NaCl, pH 6.7
SUMO-B3GlcAT1 **17S** (2 mg/mL) in 50 mM $NaH_2PO_4 \cdot 2\ H_2O$, 0.5 M NaCl, pH 6.6
Stock solution (40 µL total volume): 18.6 µL of reaction buffer (200 mM MOPS, 0.5 M NaCl, pH 7), 5.6 µL of **18S** (1.8 mg/mL), 5.8 µL of **20S** (1.72 mg/mL), 1.5 µL of UDP-Gal (100 mg/mL in H_2O), 4 µL of BSA (10 mg/mL in reaction buffer), 4.0 µL of alkaline phosphatase (100 mU/µL in reaction buffer) and 0.6 µL $MnCl_2$ (10 mg/mL in reaction buffer) were combined and vortexed prior to use.

Glycopeptide hydrazide **23** (0.3 mg, 43 nmol) was dissolved in 17.2 µL of stock solution and incubated at 30 °C. The resulting reaction conditions were: **23** (2.5 mM), **18S** (0.3 mg/mL), **20S** (0.3 mg /mL), UDP-Gal (10 mM), BSA (1 mg/mL), $MnCl_2$ (1 mM), alkaline phosphatase (10 mU/µL). After 7 h of total reaction time UDP-GlcA (0.55 µL of stock solution 100 mg/mL in H_2O, 86 nmol) and **17S** (2.6 µL) were added. Before measuring the samples via LCMS, the samples were mixed 1:1 with 100 mM DTT and 10 % MeCN/H_2O + 0.1 % HCOOH were added, resulting in a concentration of 0.5 mg/mL

LC-MS after 7 h (Hydrosphere C18 S-2 µm, 2.0 × 50 mm, 5-25% MeCN/H_2O +0.1% HCOOH). ESI-MS of **23**: *m/z* (exact mass) $C_{292}H_{465}N_{65}O_{127}S_2$ (6978.14), calculated: 1396.63 $[M+5H]^{5+}$, found: 1397.19.
ESI-MS of **23**+1Gal: *m/z* (exact mass) $C_{298}H_{475}N_{65}O_{132}S_2$ (7140.19), calculated: 1429.04 $[M+5H]^{5+}$, found: 1429.79.
ESI-MS of **23**+2Gal: *m/z* (exact mass) $C_{304}H_{485}N_{65}O_{137}S_2$ (7302.24), calculated: 1461.45 $[M+5H]^{5+}$, found: 1462.17.

ESI-MS of **23**+3Gal: *m/z* (exact mass) $C_{310}H_{495}N_{65}O_{142}S_2$ (7464.30), calculated: 1493.86 $[M+5H]^{5+}$, found: 1494.65.

LC-MS after 24 h (Hydrosphere C18 S-2 µm, 2.0 × 50 mm, 5-25% MeCN/H_2O +0.1% HCOOH).

ESI-MS of **23**: *m/z* (exact mass) $C_{292}H_{465}N_{65}O_{127}S_2$ (6978.14), calculated: 1396.63 $[M+5H]^{5+}$, found: 1397.18.

ESI-MS of **23**+1Gal: *m/z* (exact mass) $C_{298}H_{475}N_{65}O_{132}S_2$ (7140.19), calculated: 1429.04 $[M+5H]^{5+}$, found: 1429.81.

ESI-MS of **24**: *m/z* (exact mass) $C_{310}H_{493}N_{65}O_{143}S_2$ (7478.27), calculated: 1496.65 $[M+5H]^{5+}$, found: 1497.41.

ESI-MS of **25a**: *m/z* (exact mass) $C_{316}H_{501}N_{65}O_{149}S_2$ (7654.31), calculated: 1531.86 $[M+5H]^{5+}$, found: 1532.58.

ESI-MS of **25b**: *m/z* (exact mass) $C_{322}H_{511}N_{65}O_{154}S_2$ (7816.36), calculated: 1564.27 $[M+5H]^{5+}$, found: 1564.98.

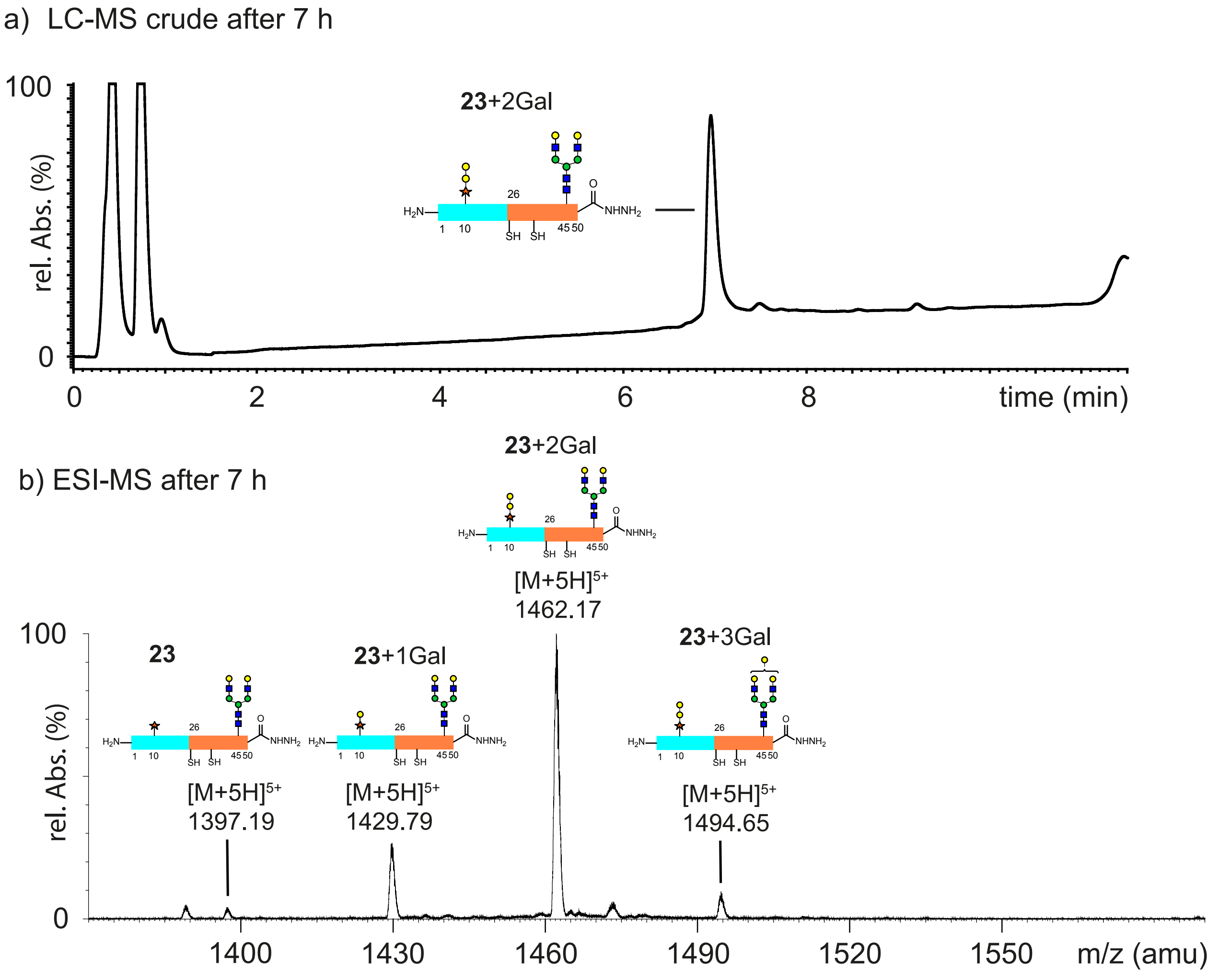

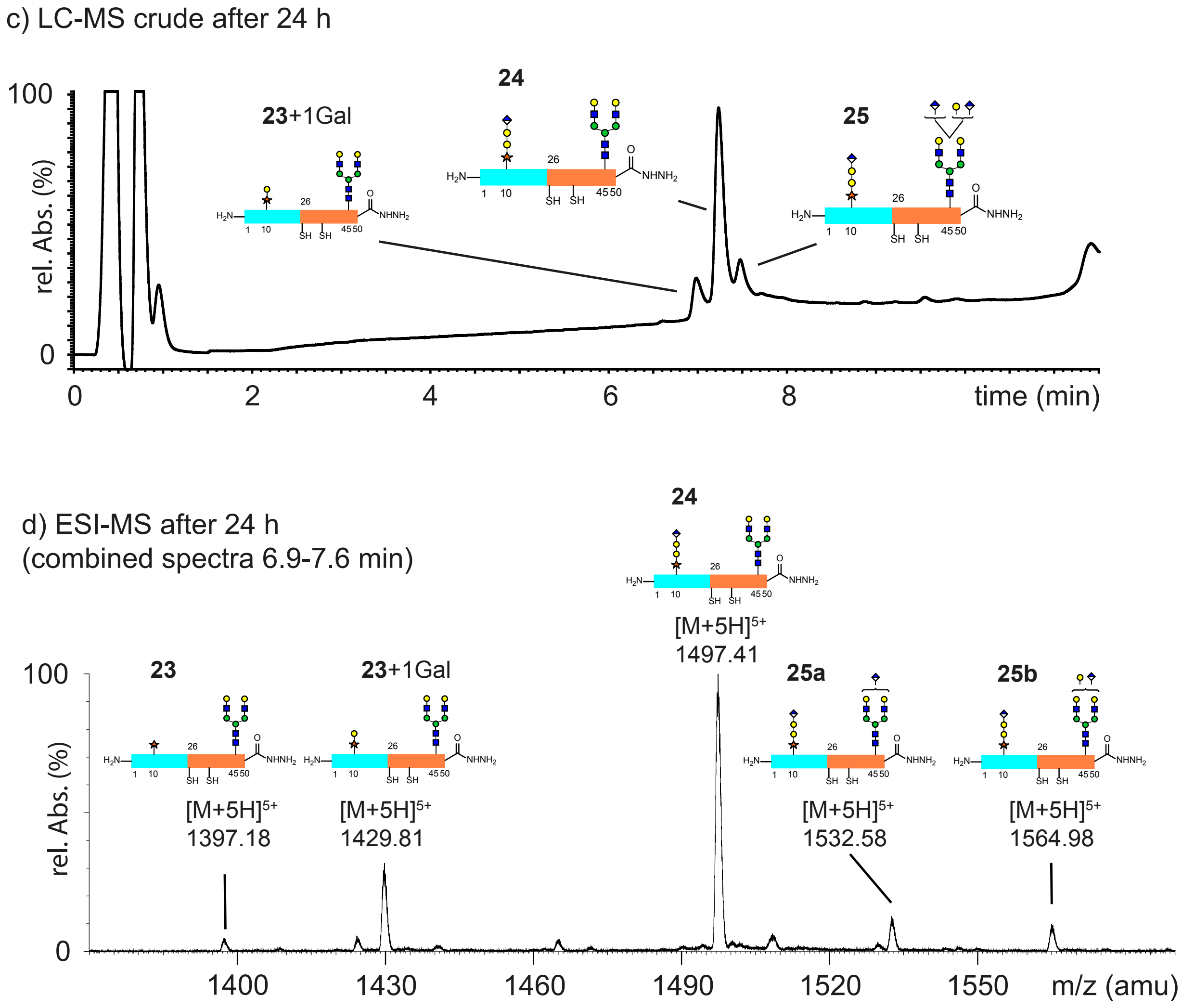

Figure S34: a) LC-MS (UV) reaction control of enzymatic galactosylation of *N,O*-glycopeptide hydrazide 1-50 **23** using the galactosyltransferases **18S** and **19S** after 7 h and b) corresponding ESI-MS showing the expected diglactosylated product (**23**+2Gal) accompanied by traces of starting material **23**, a monogalactosylated intermediate (**23**+Gal) and a tentatively assigned unexpected triglactosylated species (**23**+3Gal). c) LC-MS (UV) reaction control after addition of glucuronyltransferase **17S** + UDP-GlcA after 24 h of total reaction time and d) corresponding combined ESI-MS spectra showing the target compound **24** and side products **25a** and **25b** with additional glycosylations tentatively assigned to the N-glycan part.

7.4 Enzymatic Glucuronylation of N-Glycan Azide 26

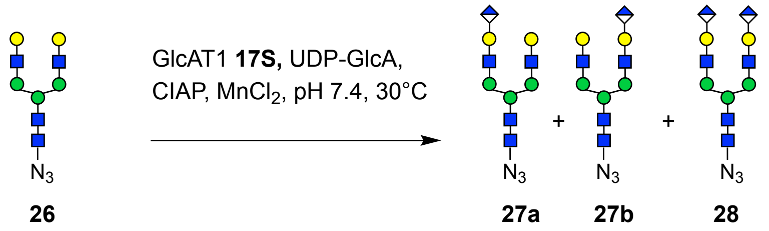

Figure S35: Incubation of N-glycan azide **26** with glucuronyltransferase **17S** and UDP-GlcA confirms the side reactions in the N-glycan part of bikunin 1-50 **23** (vide supra).

analytical reaction:

reaction buffer: 500 mM NaCl, 50 mM MES, pH 6.7

SUMO-B3GlcAT1 **17S** (1.4 mg/mL in 0.5 M NaCl, 20 mM MES, pH 6.7)

To nonasaccharide azide **26** (0.2 mg in 3.3 µL of H_2O, 120 nmol) were subsequently added 25 µL of reaction buffer, 6.46 µL of UDP-GlcA (60 mg/mL in H_2O, 600 nmol), 0.4 µL of $MnCl_2$ (100 mM), 1.8 µL of alkaline phosphatase (22 U/µL), 2 µL of BSA (20 mg/mL) and 4.3 µL of **17S**. The resulting reaction conditions were: **26** (3 mM) and UDP-GlcA (15 mM). The mixture was incubated for 3d at 30°C and analyzed by RP-HPLC-HRMS (YMC-UltraHT Hydrosphere C18, 2.0 x 30 mm 0-10 % MeCN/H_2O + 0.1 % HCOOH).

HR-ESI-MS of **27ab**: *m/z* (exact mass) $C_{68}H_{111}N_7O_{51}$ (1841.6307), calculated: 1842.6380 $[M+H]^+$, 921.8227 $[M+2H]^{2+}$, found: 1842.6338, 921.8219.

HR-ESI-MS of **28**: *m/z* (exact mass) $C_{74}H_{119}N_7O_{57}$ (2017.6628), calculated: 2018.7601 $[M+H]^+$, 1009.8387 $[M+2H]^{2+}$, found: 2018.6658, 1009.8382.

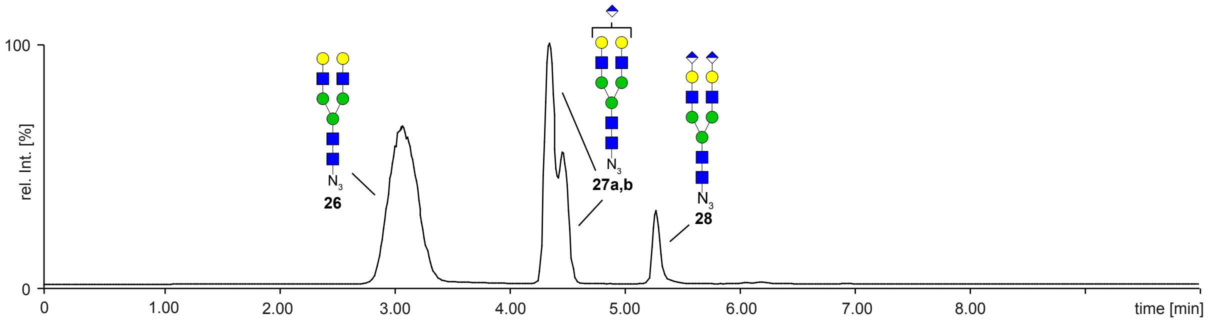

Figure S36: Reaction control of enzymatic glucuronylation of **26** by LC-HRMS (TIC) after 3d of total reaction time.

preparative reaction:

Reaction buffer: 200 mM MOPS, 0.5 M NaCl, pH 7

SUMO-B3GlcAT1 **17S** (2.2 mg/mL) in 50 mM $NaH_2PO_4 \cdot 2\ H_2O$, 0.5 M NaCl, pH 6.6

Stock solution (120 µL total volume): 54.9 µL of reaction buffer (200 mM MOPS, 0.5 M NaCl, pH 7), 32.7 µL of **17S** (2.2 mg/mL), 18.6 µL of UDP-GlcA (500 mg/mL in H_2O), 6 µL of BSA (20 mg/mL in reaction buffer), 6 µL of alkaline phosphatase (200 mU/µL in reaction buffer) and 1.7 µL of $MnCl_2$ (10 mg/mL in reaction buffer) were combined and vortexed prior to use.

Nonasaccharide azide **26** (5.34 mg, 3.20 µmol) was dissolved in 106.8 µL of stock solution and incubated at 30 °C. The resulting reaction conditions were: **A** (30 mM), **17S** (0.6 mg/mL), UDP-GlcA

(120 mM), BSA (1 mg/mL), $MnCl_2$ (1 mM), alkaline phosphatase (10 mU/µL). After 16 h UDP-GlcA (8.3 µL of stock solution 500 mg/mL in H_2O, 6.40 µmol) and **17S** (15.6 µL) were added.

After 94 h of total reaction time UDP-GlcA (8.3 µL of stock solution 500 mg/mL in H_2O, 6.40 µmol) and **17S** (15.6 µL) was added to the reaction. The reaction mixture was purified after 118 h of total reaction time by PGC-HPLC (Hypercarb, 150 x 10 mm, 15 % over 2.4 min, 15 – 20 % over 4.8 min, 20 % over 2.4 min, 20-26 % over 28.8 min, 26 % over 2.4 min, 26 – 50 % over 9.6 min, 50 % over 2.4 min, 50 – 95 % over 2.2 min, 95 % over 5 min MeCN/H_2O +0.1% HCOOH, 70 °C, flow rate: 5 mL/min. Yield: 0.56 mg of **27a** (304 nmol, 9.5 %). 1.71 mg of **27b** (928 nmol, 29 %), 1.77 mg of **28** (877 nmol, 27.4 %).

LC-MS of **27a** (Hypercarb S-3 µm, 2.1 × 30 mm, 250 Å, 10-30 % MeCN/H_2O +0.1% HCOOH, 40 °C). ESI-MS of **27a**: *m/z* (exact mass) $C_{68}H_{111}N_7O_{51}$ (1841.63), calculated: 1842.63 $[M+H]^+$, 921.82 $[M+2H]^{2+}$, found: 1842.47, 921.75.

LC-MS of **27b** (Hypercarb S-3 µm, 2.1 × 30 mm, 250 Å, 10-30 % MeCN/H_2O +0.1% HCOOH, 40 °C). ESI-MS of **27b**: *m/z* (exact mass) $C_{68}H_{111}N_7O_{51}$ (1841.63), calculated: 1842.63 $[M+H]^+$, 921.82 $[M+2H]^{2+}$, found: 1842.63, 921.82.

LC-MS of **28** (Hypercarb S-3 µm, 2.1 × 30 mm, 250 Å, 10-30 % MeCN/H_2O +0.1% HCOOH, 40 °C). ESI-MS of **27a**: *m/z* (exact mass) $C_{74}H_{119}N_7O_{57}$ (2017.66), calculated: 2018.66 $[M+H]^+$, 1009.83 $[M+2H]^{2+}$, found: 2019.53, 1009.78.

a) PGC-HPLC (Hypercarb)

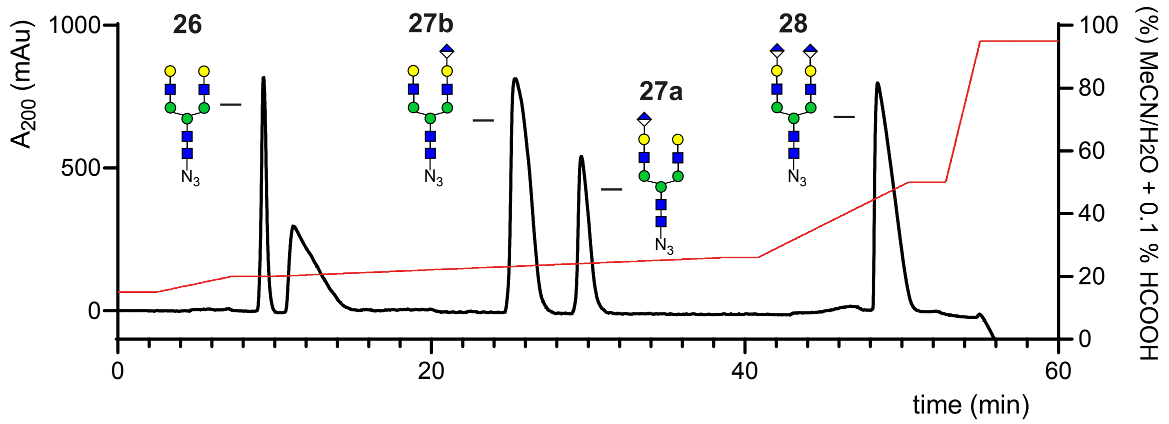

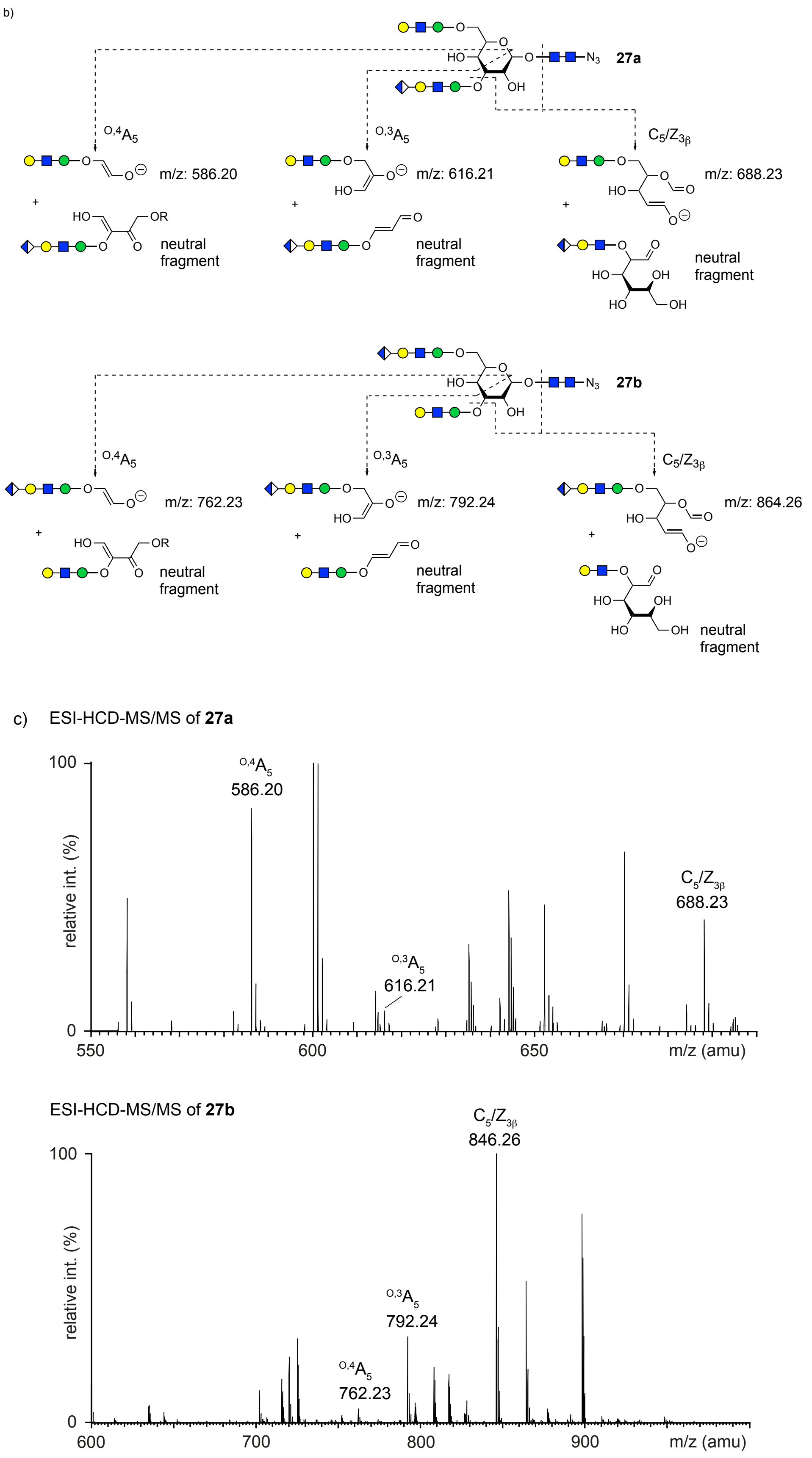

Figure S37: a) preparative separation by PGC-HPLC (Hypercarb, 150 x 10 mm), b) anionic cross ring fragmentation patterns for **27a** and **27b** leads to characteristic, diagnostic ions, c) formation of diagnostic ions from unsymmetric glucuronides **27a** and **27b** by HR-HCD-MS/MS in negative mode.[18]

7.5 Enzymatic Galactosylation of N-Glycan Azide 26

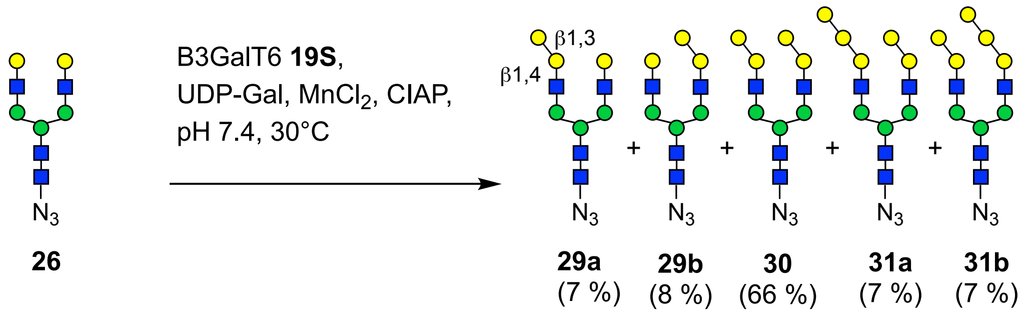

Figure S38: Incubation of N-glycan azide **26** with galactosyltransferase B3GalT6 **19S** and UDP-Gal confirms the side reactions in the N-glycan part of bikunin 1-50 **23** (vide supra). Additionally, the enzyme is capable of poly β1,3-galactosylating the N-glycan in both arms.

preparative reaction:

reaction buffer: 500 mM NaCl, 50 mM MES, pH 6.7

SUMO-B3GalT6 **19S** (2.8 mg/mL in 0.5 M NaCl, 20 mM MES, pH 6.7)

To nonasaccharide azide **26** (4.4 mg, 2.6 µmol) were subsequently added 151.4 µL of reaction buffer, 80.6 µL of UDP-Gal (60 mg/mL in H_2O, 7.9 µmol), 6.6 µL of $MnCl_2$ (50 mM), 15 µL of alkaline phosphatase (22 U/µL), 16.5 µL of BSA (20 mg/mL) and 60 µL of **19S**. The resulting reaction conditions were: **26** (8 mM) and UDP-GlcA (24 mM). The mixture was incubated for 2 d at 30°C, analyzed by LC-MS (YMC-UltraHT Hydrosphere C18, 2.0 x 30 mm, 0-3 % MeCN/H_2O + 0.1 % HCOOH and purified by gel filtration (Superdex 30 pg, 16 x 600, 1.5 mL/min, 0.1 M NH_4HCO_3). The lyophilized product mixture was fractionated by preparative RP-HPLC (YMC Hydrosphere C18, 10 x 150mm, 0-5 % MeCN/H_2O + 0.1 % HCOOH). The lyophilized product fractions were separated by an additional RP-HPLC (Thermo Scientific Hypercarb, 4.6 x 100 mm, 5-25 % MeCN/H_2O + 0.1 % HCOOH) and lyophilized. Yields: 0.33 mg of **29a** (0.18 µmol, 7 %), 0.37 mg of **29b** (0.2 µmol, 8 %), 3.4 mg of **30** (1.71 µmol, 66 %), 0.41 mg of **31a** (0.19 µmol, 7 %), 0.40 mg of **31b** (0.19 µmol, 7 %). The purity of the fractions was analyzed by LC-MS (Hypercarb S-3 µm, 2.1 × 30 mm, 250 Å, 5-25 % MeCN/H_2O +0.1% HCOOH, 40 °C) and HR-MS (direct injection in H_2O +0.1% HCOOH).

HR-ESI-MS of **29a**: *m/z* (exact mass) $C_{68}H_{113}N_7O_{50}$ (1827.6515), calculated: 1828.6588 $[M+H]^+$, 914.8330 $[M+2H]^{2+}$, found: 1828.6577, 914.8370.

HR-ESI-MS of **29b**: *m/z* (exact mass) $C_{68}H_{113}N_7O_{50}$ (1827.6515), calculated: 1828.6588 $[M+H]^+$, 914.8330 $[M+2H]^{2+}$, found: 1828.6559, 914.8351.

HR-ESI-MS of **30**: *m/z* (exact mass) $C_{74}H_{123}N_{7}O_{55}$ (1989.7043), calculated: 995.8594 $[M+2H]^{2+}$, found: 995.8598.

HR-ESI-MS of **31a**: *m/z* (exact mass) $C_{80}H_{133}N_{7}O_{60}$ (2151.7471), calculated: 1076.8859 $[M+2H]^{2+}$, found: 1076.8835.

HR-ESI-MS of **31b**: *m/z* (exact mass) $C_{80}H_{133}N_{7}O_{60}$ (2151.7471), calculated: 1076.8859 $[M+2H]^{2+}$, found: 1076.8835.

30: ^{1}H-NMR (500 MHz, D_2O with [D$_4$]-methanol as internal standard): δ = 5.12 (d, 1H, $J_{1,2} < 1$ Hz, H-1^{4}α), 4.93 (d, 1H, $J_{1,2} < 1$ Hz, H-1$^{4'}$α), 4.77-4.74 (m, 2H, H-1^{3}β, H-1^{1}β), 4.65-4.55 (m, 3H, H-1^{7}β, H-1$^{7'}$β, H-1^{2}β, H-1^{5}β, H-1$^{5'}$β), 4.55-4.50 (m, 2H, H-1^{6}β, H-1$^{6'}$β), 4.25 (dd, 1H, $J_{1,2} < 1$ Hz, $J_{2,3} < 1$ Hz, H-2^{3}), 4.20-4.17 (m, 3H, H-4^{6}, H-4$^{6'}$, H-2^{4}), 4.11 (dd, 1H, $J_{1,2} < 1$ Hz, $J_{2,3} < 1$ Hz, H-2$^{4'}$), 4.06-3.53 (m, 59H, H-6a^{5}, H-6a$^{5'}$, H-6a^{3}, H-4^{7}, H-4$^{7'}$, H-6a^{4}, H-6a$^{4'}$, H-3^{4}, H- 3$^{4'}$, H-6b^{5}, H-6b$^{5'}$, H-6a^{2}, H-3^{6}, H-3$^{6'}$, H-6a^{1}, H-2^{2}, H-6b^{3}, H-3^{3}, H-6a,b^{6}, H-6a,b$^{6'}$, H-4^{2}, H- 2^{1}, H-4^{5}, H-4$^{5'}$, H-6a,b^{7}, H-6a,b$^{7'}$, H-5^{6}, H-5$^{6'}$, H-4^{3}, H-6b^{2}, H-3^{1}, H-5^{4}, H-3^{2}, H-2^{5}, H-2$^{5'}$, H- 2^{6}, H-2$^{6'}$, H-5^{7}, H-5$^{7'}$, H-6b^{1}, H-3^{5}, H-3$^{5'}$, H-3^{7}, H-3$^{7'}$, H-4^{1}, H-5$^{4'}$, H-5^{2}, H-5^{3}, H-6b^{4}, H-6b$^{4'}$, H-2^{7}, H-2$^{7'}$, H-5^{1}, H-5^{5}, H-5$^{5'}$), 3.53-3.45 (m, 2H, H-4^{4}, H-4$^{4'}$), 2.10-2.02 (m, 12H, NAc).

^{13}C-NMR (125 MHz, D_2O D_2O with [D$_4$]-methanol as internal standard): δ = 175.8, 175.7, 175.6 (C=O NAc), 105.3 (C-1^{7}β, $^{1}J_{C\text{-}1,H\text{-}1}$ = 161.2 Hz), 101.3 (C-1$^{7'}$β, $^{1}J_{C\text{-}1,H\text{-}1}$ = 161.2 Hz), 103.5 (C-1^{6}β, $^{1}J_{C\text{-}1,H\text{-}1}$ = 162.8 Hz), 103.5 (C-1$^{6'}$β, $^{1}J_{C\text{-}1,H\text{-}1}$ = 161.7 Hz), 102.3 (C-1^{2}β, $^{1}J_{C\text{-}1,H\text{-}1}$ = 162.5 Hz), 101.4 (C-1^{3}β, $^{1}J_{C\text{-}1,H\text{-}1}$ = 160.7 Hz), 100.5 (C-1^{4}α, $^{1}J_{C\text{-}1,H\text{-}1}$ = 171.3 Hz), 100.4 (C-1^{5}β, $^{1}J_{C\text{-} 1,H\text{-}1}$ = 162.8 Hz), 100.4 (C-1$^{5'}$β, $^{1}J_{C\text{-}1,H\text{-}1}$ = 164.5 Hz), 98.0 (C-1$^{4'}$α, $^{1}J_{C\text{-}1,H\text{-}1}$ = 170.6 Hz), 89.5 (C-1^{1}β, $^{1}J_{C\text{-}1,H\text{-}1}$ = 158.7 Hz), 82.9 (C-3^{6}), 82.9 (C-3$^{6'}$), 81.4 (C-3^{3}), 80.4 (C-4^{2}), 79.6 (C-4^{1}), 79.2 (C-4^{5}), 79.2 (C-4$^{5'}$), 77.4 (C-5^{1}), 77.3 (C-2^{4}), 77.2 (C-2$^{4'}$), 76.0 (C-5^{7}), 76.0 (C-5$^{7'}$), 76.0 (C-5^{6}), 76.0 (C-5$^{6'}$),75.7 (C-5^{5}), 75.7 (C-5$^{5'}$), 75.4 (C-5^{2}), 75.4 (C-5^{3}), 74.5 (C-5^{4}), 73.8 (C-5$^{4'}$), 73.5 (C-3^{5}), 73.5 (C-3$^{5'}$),73.2 (C-3^{7}), 73.2 (C-3$^{7'}$), 73.0 (C-3^{1}), 73.0 (C-3^{2}), 72.0 (C-2^{7}), 72.0 (C-2$^{7'}$), 71.2 (C-2^{6}), 71.2 (C-2$^{6'}$), 71.2 (C-2^{3}), 70.4 (C-3^{4}), 70.4 (C-3$^{4'}$), 69.5 (C-4^{7}), 69.5 (C- 4$^{7'}$), 69.4 (C-4^{6}), 69.4 (C-4$^{6'}$), 68.3 (C-4^{4}), 68.3 (C-4$^{4'}$), 66.7 (C-6^{3}), 62.6 (C-6^{4}), 62.6 (C-6$^{4'}$), 62.0 (C-6^{7}), 62.0 (C-6$^{7'}$), 62.0 (C-6^{6}), 62.0 (C-6$^{6'}$), 60.9 (C-6^{1}), 60.9 (C-6^{2}), 60.9 (C-6^{5}), 60.9 (C-6$^{5'}$), 55.9 (C-2^{2}), 55.8 (C-2^{5}), 55.8 (C-2$^{5'}$), 55.5 (C-2^{1}), 23.3, 23.2, 23.1 (NAc).

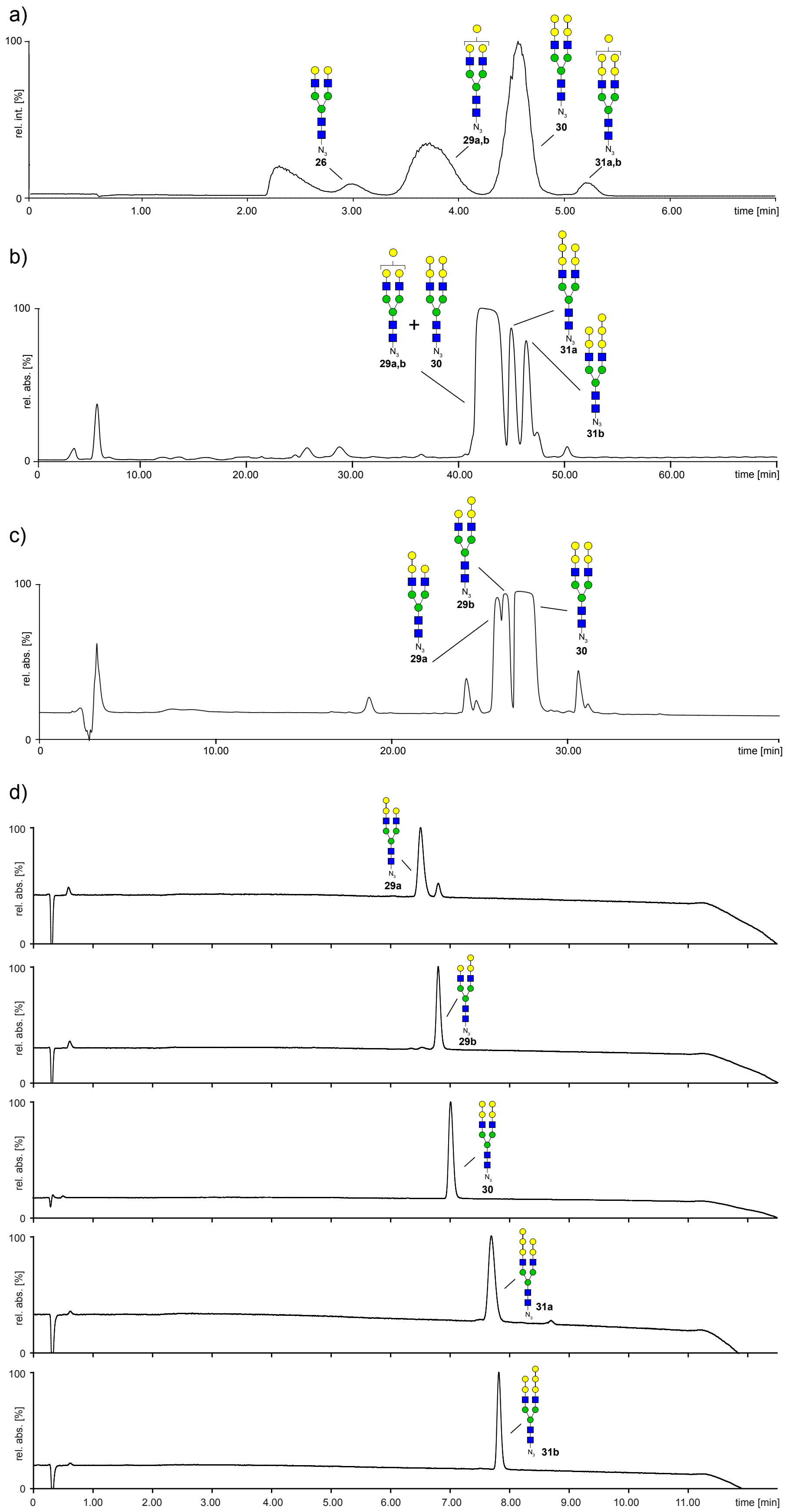

Figure S39: a) Analytical RP-LC-MS (TIC) of the crude reaction mixture after 24 h, b) preparative separation by C18-RP-HPLC (c) preparative separation by Hypercarb-RP-HPLC, d) analytical RP-LC-MS (UV) of the purified compounds.

a)

b)

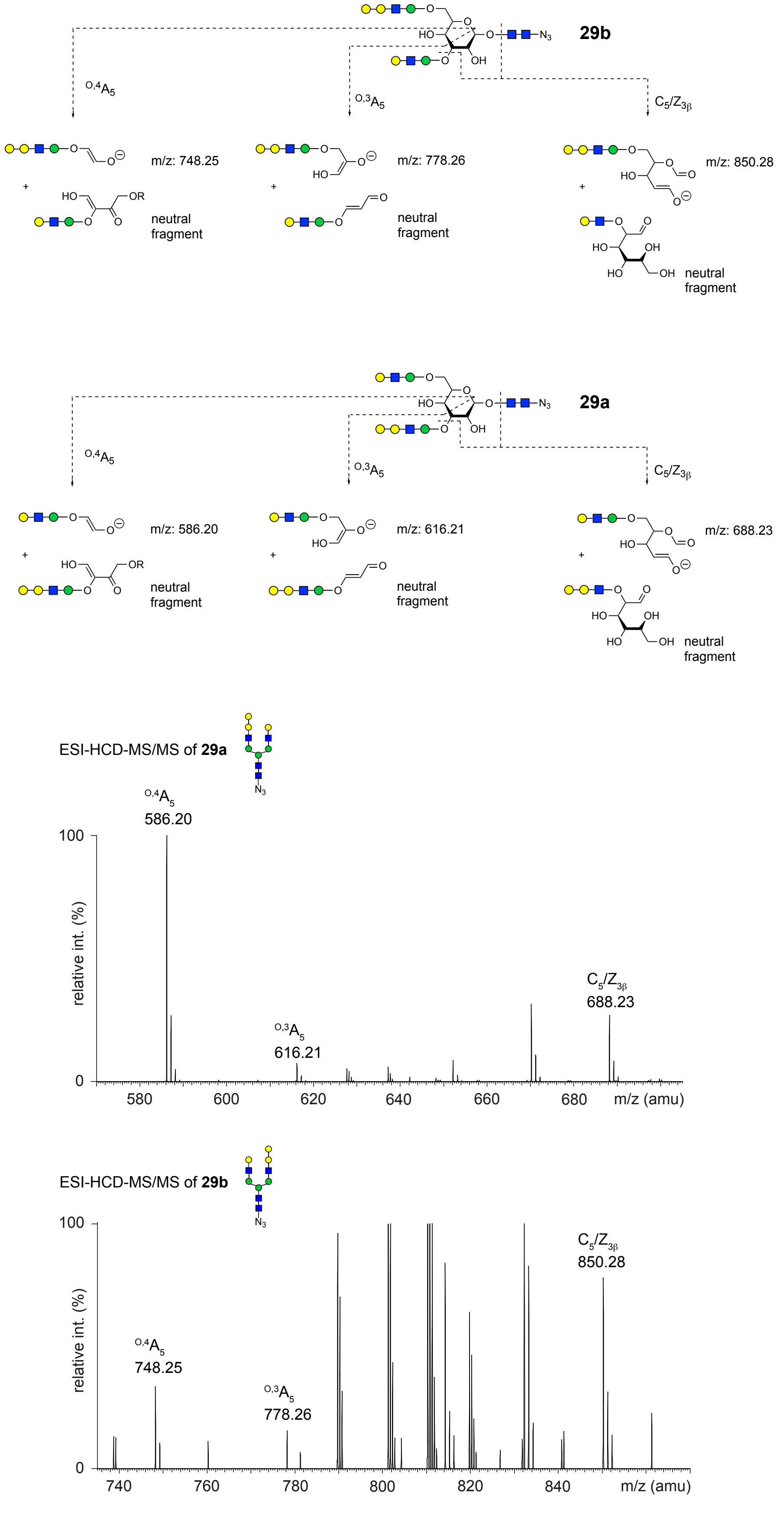

29b
O,4A5
O,3A5
C5/Z3β
m/z: 748.25
m/z: 778.26
m/z: 850.28
neutral fragment
neutral fragment
neutral fragment
29a
m/z: 586.20
m/z: 616.21
m/z: 688.23
ESI-HCD-MS/MS of 29a
O,4A5
586.20
O,3A5
616.21
C5/Z3β
688.23
relative int. (%)
m/z (amu)
ESI-HCD-MS/MS of 29b
O,4A5
748.25
O,3A5
778.26
C5/Z3β
850.28
relative int. (%)
m/z (amu)

c)

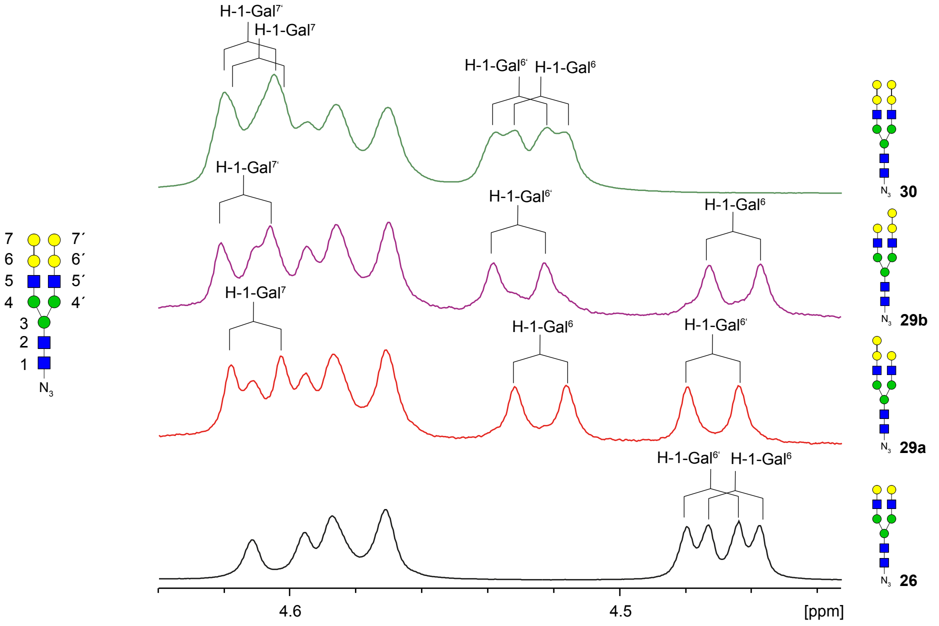

d)

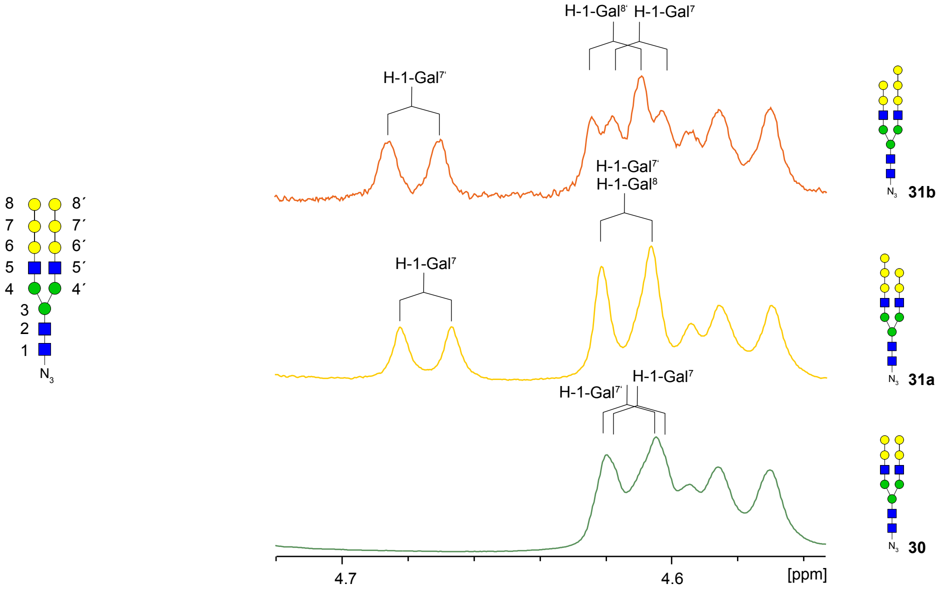

Figure S40: a) anionic cross ring fragmentation patterns for **29a** and **29b** leads to characteristic diagnostic ions, b) HR-HCD-MS/MS in negative mode[18] provides the expected diagnostic ions permitting assignment of the regioisomeric galactosides **29a** and **29b**, c) 500MHz-^{1}H-NMR assignment of unsymmetric N-glycans **29a** and **29b** via characteristic upfield shift of anomeric signals of Gal^{6} and Gal^{7} residues in the 1,3-arm relative to anomeric signals of Gal$^{6'}$ and Gal$^{7'}$ residues in the 1,6-arm; the effect is most pronounced in the parent compound **26**, d) 500MHz-^{1}H-NMR assignment of unsymmetric N-glycans **31a** and **31b** via characteristic upfield shift of the anomeric signal of Gal^{8} in the 1,3-arm relative to anomeric signal of Gal$^{8'}$ in the 1,6-arm.

7.6 Enzymatic Synthesis of Bikunin 1-25 Glycopeptides with extended linkage region

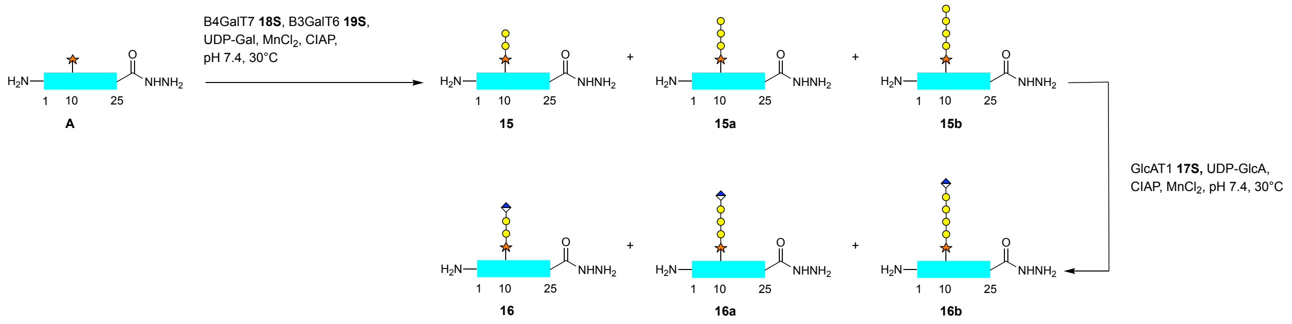

Figure S41: Analytical scale synthesis of bikunin 1-25 glycopeptides with an extended linkage region (**15a, 15b**) from glycopeptide **A**. The resulting glycopeptides were accepted by B3GlcAT1 17S and readily converted to the corresponding bikunin 1-25 glycopeptides with a terminal glucuronic acid (**16**, **16a**, **16b**).

Buffers and solutions:

Reaction buffer: 200 mM MOPS, 0.5 M NaCl, pH 7

SUMO-B4GalT7 **18S** (1.8 mg/mL in 50 mM NaH_2PO_4, 0.5 M NaCl, pH 6.6)

SUMO-B3GalT6 **20S** (1.7 mg/mL in 20 mM Mes, 1 M NaCl, pH 6.7)

SUMO-B3GlcAT1 **17S** (2 mg/mL in 50 mM NaH_2PO_4, 0.5 M NaCl, pH 6.6)

Stock solution (50 µL total volume): 1.6 µL of reaction buffer, 5.6 µL of **18S** (1.8 mg/mL), 17.4 µL of **20S** (1.72 mg/mL), 14.7 µL of UDP-Gal (100 mg/mL in H_2O), 5.0 µL of BSA (10 mg/mL in reaction buffer), 5.0 µL of alkaline phosphatase (100 mU/µL in reaction buffer) and 0.7 µL $MnCl_2$ (10 mg/mL in reaction buffer) were combined and vortexed prior to use.

Glycopeptide hydrazide **A** (0.37 mg, 135.3 nmol) was dissolved in 22.6 µL of stock solution and incubated at 30 °C. The resulting reaction conditions were: **A** (6 mM), UDP-Gal (48 mM), $MnCl_2$ (1 mM). After a total reaction time of 2 hours 6.6 µL of UDP-Gal (100 mg/mL in H_2O, 1.08 µmol) and 6.6 µL of **20S** were added. After a total reaction time of 24 hours an aliquot was analyzed by LC-MS and 3.5 µL of UDP-GlcA (100 mg/mL in H_2O, 541 nmol) and 8.9 µL of **17S** were added. After a total reaction of 27 hours the final reaction mixture was analyzed by LC-MS.

LC-MS after 24 h (Hydrosphere C18 S-2 µm, 2.0 × 50 mm, 5-25% MeCN/H_2O +0.1% HCOOH).

ESI-MS of **15**: *m/z* (exact mass) $C_{125}H_{209}N_{31}O_{57}$ (3056.44), calculated: 765.11 $[M+4H]^{4+}$, found: 765.33.

ESI-MS of **15a**: *m/z* (exact mass) $C_{131}H_{219}N_{31}O_{62}$ (3218.49), calculated: 805.62 $[M+4H]^{4+}$, found: 805.65.

ESI-MS of **15b**: *m/z* (exact mass) $C_{137}H_{229}N_{31}O_{67}$ (3380.55), calculated: 846.14 $[M+4H]^{4+}$, found: 846.39.

LC-MS after 27 h (Hydrosphere C18 S-2 µm, 2.0 × 50 mm, 5-25% MeCN/H_2O +0.1% HCOOH).

ESI-MS of **16**: *m/z* (exact mass) $C_{131}H_{217}N_{31}O_{63}$ (3232.47), calculated: 809.12 $[M+4H]^{4+}$, found: 809.37.

ESI-MS of **16a**: *m/z* (exact mass) $C_{137}H_{227}N_{31}O_{68}$ (3394.53), calculated: 849.63 $[M+4H]^{4+}$, found: 849.89.

ESI-MS of **16b**: *m/z* (exact mass) $C_{143}H_{237}N_{31}O_{73}$ (3556.58), calculated: 890.15 $[M+4H]^{4+}$, found: 890.41.

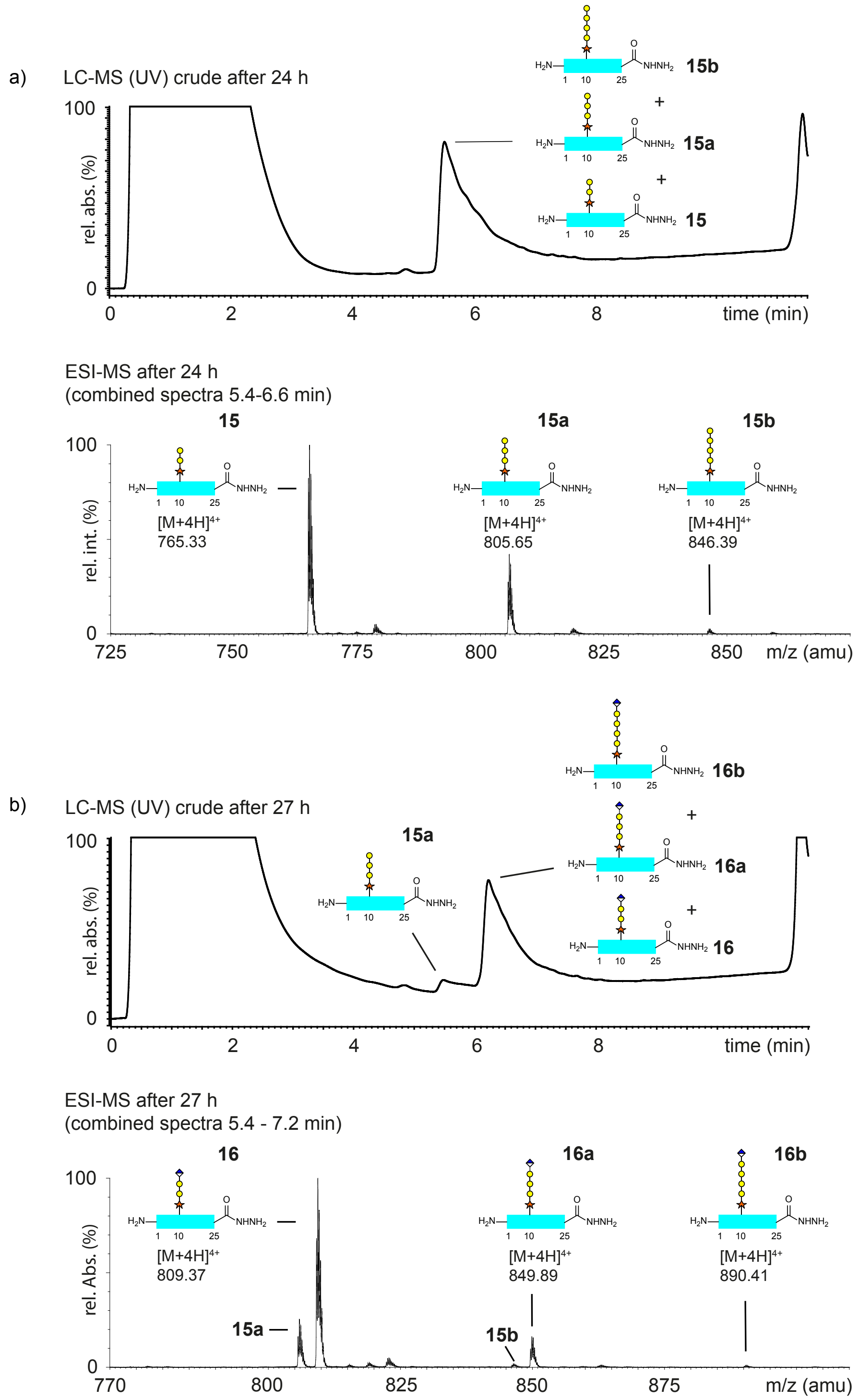

Figure S42: Reaction controls by LC-MS of a) galactosylation of **A** with B4GalT7 **18S** and B3GalT6 **20S** using 48 mM UDP-Gal for 24 h followed by b) glucuronylation using B3GlcAT1 **17S** and 24 mM UDP-GlcA for 3h.

7.7 Preparative Synthesis of Bikunin 1-25 Glycopeptide 22 with truncated linkage region

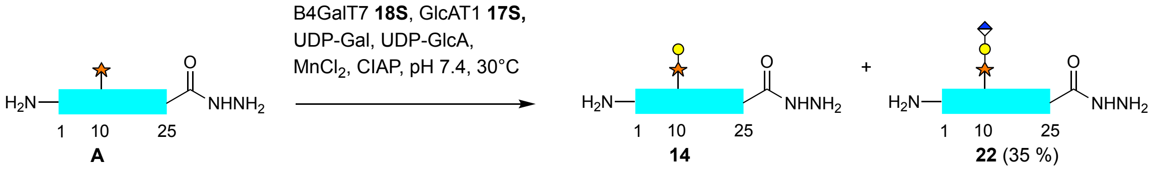

Figure S43: Preparative synthesis of the truncated bikunin 1-25 glycopeptide **22** from glycopeptide **A**.

Reaction buffer: 200 mM MOPS, 0.5 M NaCl, pH 7

SUMO-B4GalT7 **18S** (1.8 mg/mL) in 50 mM $NaH_2PO_4 \cdot 2\ H_2O$, 0.5 M NaCl, pH 6.6

SUMO-B3GlcAT1 **17S** (2 mg/mL) in 50 mM $NaH_2PO_4 \cdot 2\ H_2O$, 0.5 M NaCl, pH 6.6

Stock solution (350 µL total volume): 51.2 µL of reaction buffer (200 mM MOPS, 0.5 M NaCl, pH 7), 38.9 µL of **18S** (1.8 mg/mL), 105 µL of **17S** (2 mg/mL), 25.6 µL of UDP-Gal (100 mg/mL in H_2O), 54.3 µL of UDP-GlcA (100 mg/mL in H_2O), 35 µL of BSA (10 mg/mL in reaction buffer), 35 µL of alkaline phosphatase (100 mU/µL in reaction buffer) and 5 µL of $MnCl_2$ (10 mg/mL in reaction buffer) were combined and vortexed prior to use.

Glycopeptide hydrazide **A** (5.26 mg, 1.92 µmol) was dissolved in 320.7 µL of stock solution and incubated at 30 °C. The resulting reaction conditions were: **A** (6 mM), **18S** (0.2 mg/mL), **17S** (0.6 mg /mL), UDP-Gal (12 mM), UDP-GlcA (24 mM), BSA (1 mg/mL), $MnCl_2$ (1 mM), alkaline phosphatase (10 mU/µL). After 16 h 49.8 µL of UDP-GlcA (100 mg/mL in H_2O, 7.68 µmol) and 96.1 µL of **17S** were added. After 30 h 12.4 µL of UDP-GlcA (1.92 µmol) and 48.1 µL of **17S** were added. After 41 h 24.9 µL of UDP-GlcA (3.84 µmol) and 96.1 µL of **17S** were added to the reaction. The reaction mixture was purified by gel filtration (HiLoad 16/600 Superdex 30 pg, 600 × 16 mm, 10 % MeCN/H_2O + 0.1 % HCOOH, flow rate 1 mL/min). Yield: 5.15 mg of mixture **14** + **22**.

3 mg of the mixture **14** + **22** (986.3 nmol) were purified by RP-HPLC (YMC-Actus Hydrosphere C18 250 x 20 mm, S-5 µm, 12 nm, 6 %– 20 % MeCN/H_2O + 0.1 % HCOOH, flow rate: 10 mL/min). Yield: 1.21 mg of **22** (394 nmol, 35 %). LC-MS of **22** (Hydrosphere C18 S-2 µm, 2.0 × 50 mm, 5-25% MeCN/H_2O +0.1% HCOOH). ESI-MS of **22**: *m/z* (exact mass) $C_{125}H_{207}N_{31}O_{58}$ (3070.42), calculated: 768.61 $[M+4H]^{4+}$, 1024.47 $[M+3H]^{3+}$, 1536.21 $[M+3H]^{2+}$, found: 768.58, 1024.44, 1536.23.

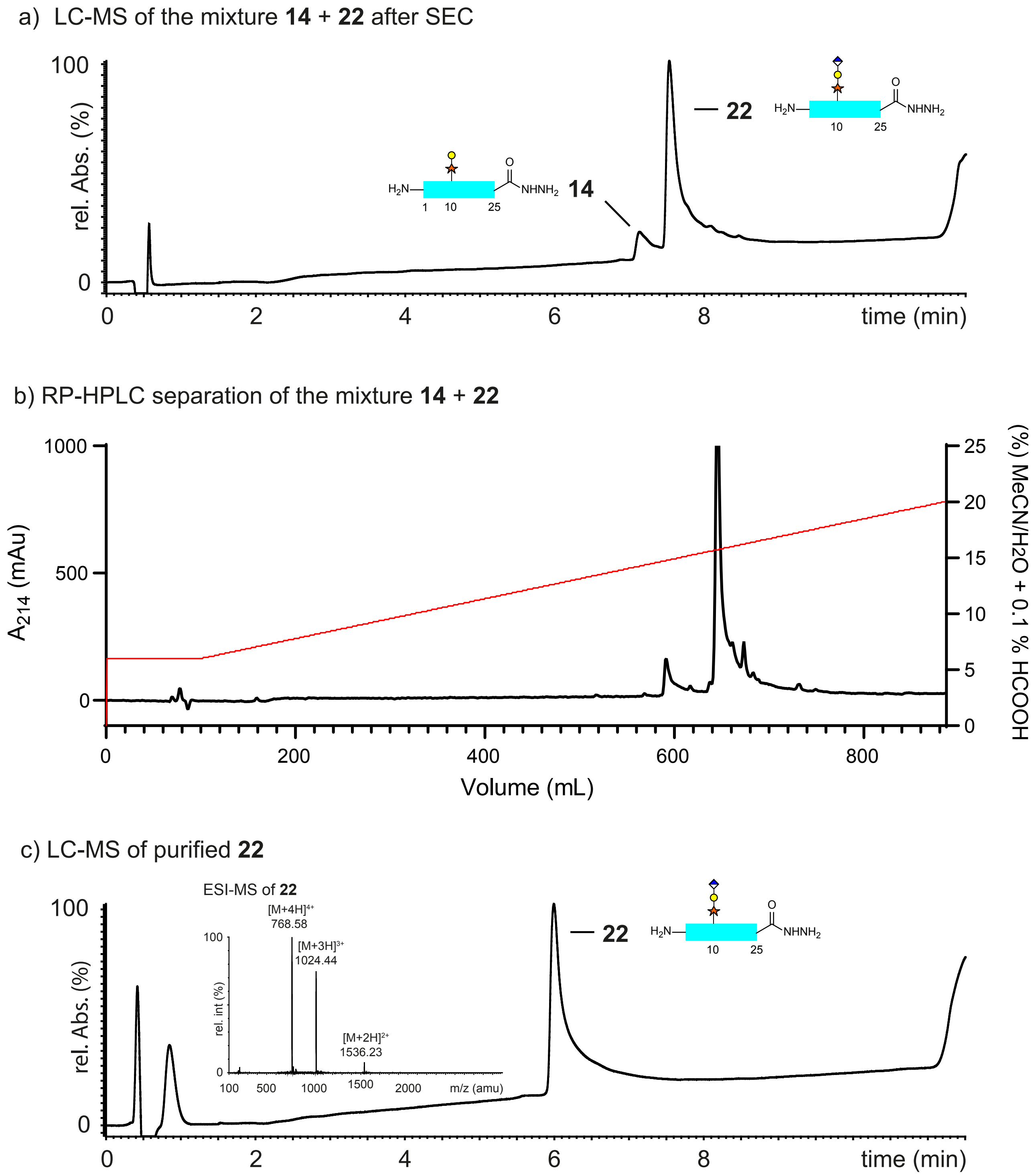

Figure S44: a) LC-MS of the truncated bikunin 1-25 glycopeptide **22** after SEC, b) preparative purification of the mixture of **14** + **22** by RP-HPLC, c) LC-MS of the purified truncated bikunin 1-25 glycopeptide **22**.

7.8 Preparative Synthesis of Bikunin 1-25 linkage region Glycopeptide 16

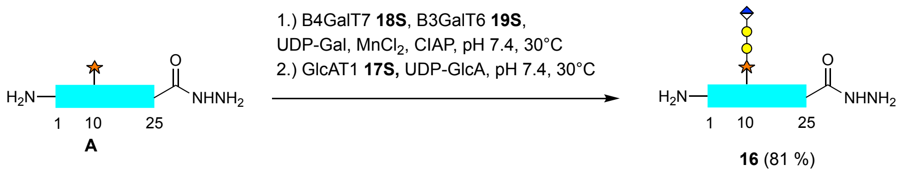

Figure S45: Preparative scale enzymatic elongation of bikunin 1-25 glycopeptide **A** to glycopeptide hydrazide **16** containing the linkage region tetrasaccharide.

Buffers and solutions:

Reaction buffer: 20 mM MES, 0.5 M NaCl, pH 6.7

SUMO-B4GalT7 **18S** (2.2 mg/mL in 1 M NaCl, 20 mM MES, pH 6.7)

SUMO-B3GalT6 **20S** (2.8 mg/mL in 1 M NaCl, 20 mM MES, pH 6.7)

SUMO-B3GlcAT1 **17S** (1.4 mg/mL in 0.5 M NaCl, 20 mM MES, pH 6.7)

Bikunin 1-25 glycopeptide hydrazide **A** (17.4 mg, 6.4 µmol) was dissolved in 779.5 µL of reaction buffer (0.5 M NaCl, 20 mM MES, pH 6.7). Subsequently,194.7 µL of UDP-Gal (60 mg/mL in H_2O 18.6 µmol,), 63.8 µL of BSA (20 mg/mL), 12.8 µL of $MnCl_2$ (100 mM), 12.8 µL of CIAP (1 U/µL), 119 µL of **18S** and 93.8 µL of **20S** were added. The resulting reaction conditions were: **A** (5 mM), UDP-Gal (15 mM). After 16 h 171.8 µL of UDP-GlcA (60 mg/mL in H_2O, 16 µmol) and 546 µL of **17S** were added. After 24 h of total reaction time the mixture was fractionated by gel filtration Superdex Peptide 10/300 GL, 0.3 mL/min, 10 % MeCN/ H_2O + 0.1 % TFA in several portions. The lyophilized product was purified by RP-HPLC (Ascentis C18, 250 x 10mm, 5-25 % MeCN/ H_2O + 0.1 % TFA, 3 mL/min) in several portions and lyophilized. Yield: 16.8 mg of **16** (5.2 µmol, 81 %). The reaction was monitored by LC-MS (Hydrosphere C18 S-2 µm, 2.0 × 50 mm, 5-25% MeCN/H_2O +0.1% HCOOH).

ESI-MS of **16**: *m/z* (average mass) $C_{131}H_{217}N_{31}O_{63}$ (3234.33), calculated: 3235.331 $[M+H]^+$, 1618.17 $[M+2H]^{2+}$found: 3231.54, 1616.12.

HR-ESI-MS of **16** (direct injection in H_2O + 0.1 % HCOOH): *m/z* (exact mass): $C_{131}H_{217}N_{31}O_{63}$ (3232.4729); calculated: $[M+3H]^{3+}$ 1078.4983, $[M+4H]^{4+}$ 809.1255, found: 1078.4949, 809.1285.

LC-MS after 24 h (Hydrosphere C18 S-2 µm, 2.0 × 50 mm, 5-20% MeCN/H_2O +0.1% HCOOH).

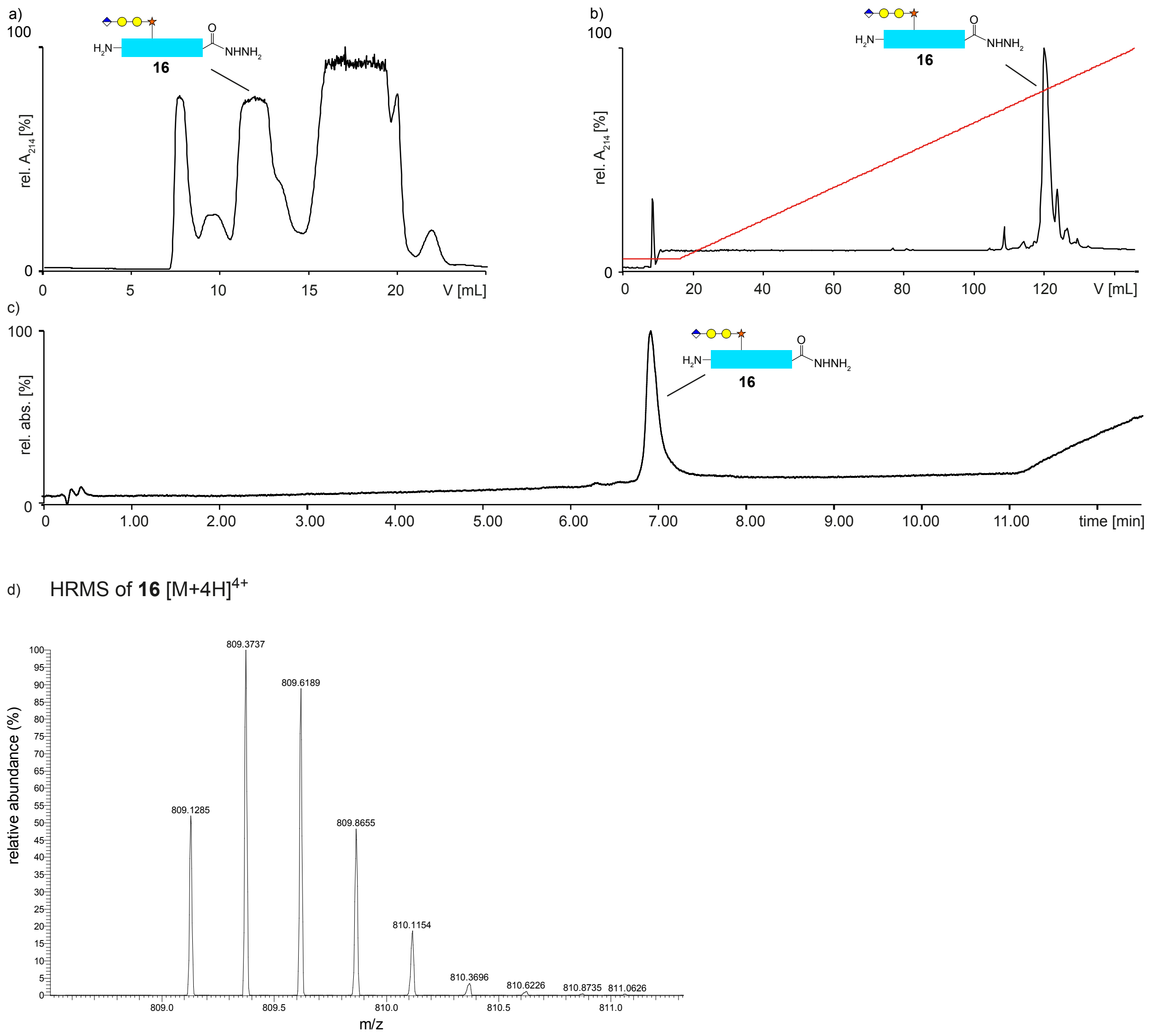

Figure S46: a) Gel filtration of bikunin 1-25 glycopeptide hydrazide **16**, b) preparative RP-HPLC of glycopeptide hydrazide **16**, c) RP-HPLC-MS of purified **16**, d) HR-MS of purified **16**.

7.9 Synthesis of Bikunin 1-50 Glycopeptide Hydrazide 24

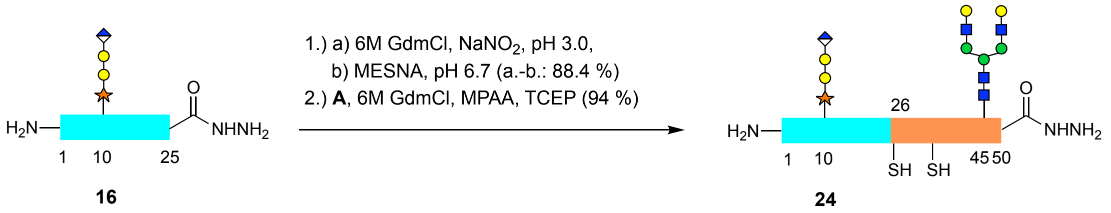

Figure S47: Synthesis of glycopeptide hydrazide 1-50 **24**.

1.) Synthesis of thioester: Bikunin 1-25 glycopeptide hydrazide **16** (2.6 mg, 0.8 µmol) was dissolved in buffer (212 µL, 6 M GdmCl, 0.2 M NaH_2PO_4, pH 3.0) and cooled to -15 °C for 10 min. Under stirring 0.5 M $NaNO_2$ (16 µL, 8 µmol) was added. The mixture was stirred for 25 min at -15 °C followed by addition of 1 M sodium 2-mercaptoethanesulfonate (80 µL, 80 µmol) in 6 M GdmCl, 0.2 M NaH_2PO_4, pH 6.6. The reaction mixture was brought to ambient temperature followed by adjustment of the pH value (4.7) to 6.7 using 1 M NaOH and a pH-microelectrode. The microelectrode remains in the mixture, After 1 h of occasional stirring the mixture was diluted with buffer (190 µL, 6 M GdmCl, 0.2 M NaH_2PO_4, pH 3) and purified by gel filtration (Sephadex G25 superfine, 15 × 164 mm, 10% MeCN/H_2O +0.1% TFA, 1.5 mL/min) gereinigt. Fractions containing the desired thioester **16MESNA** were pooled and lyophilized. Yield: 2.4 mg of **16MESNA** (0.7 µmol, 88%).

LC-MS of **16MESNA** (Hydrosphere C18 S-2 µm, 2.0 × 50 mm, 5-25% MeCN/H_2O +0.1% HCOOH). ESI-MS of **16MESNA** *m/z* (average mass) $C_{133}H_{219}N_{29}O_{66}S_2$ (3344.47), calculated: 3345.48 $[M+H]^+$, 1673.24 $[M+2H]^{2+}$ found: 3342.03, 1671.01.

2.) Native chemical ligation: Bikunin 1-25 glycopeptide thioester **16MESNA** (2.4 mg (0.7 µmol) and Bikunin 26-50 glycopeptide hydrazide **B** (1.8 mg, 0.43 µmol) were dissolved in ligation buffer (234 µL, 6 M GdmCl, 0.2 M NaH_2PO_4, 100 mM MPAA, 50 mM TCEP-HCl, pH 6.9) in an anaerobic chamber. After 25 h 2 µL of the reaction mixture were reduced with aqueous TCEP-HCl (18 µL, 50 mM, pH 6-7) and analyzed by LC-MS (Hydrosphere C18, 2.0 × 30 mm, 10-35% MeCN/H_2O + 0.1% HCOOH). After complete conversion of **B** one half of the reaction mixture (117 µL) was treated with TCEP-HCl (1 mg, 3.5 µmol) and purified by gel filtration (Superdex Peptide 10/300 GL, 10 × 300 mm, 10% MeCN/H_2O +0.1% TFA, flow rate 0.2 mL/min). The remaining half of the reaction mixture (117 µL) was treated accordingly. The fractions containing **24** were combined and lyophilized. Yield: 4.1 mg of **24** (0.55 µmol, 94%).

LC-MS of **24** (Hydrosphere C18 S-2 µm, 2.0 × 30 mm, 10-35% MeCN/H_2O +0.1% HCOOH).

HR-ESI-MS of **24**: *m/z* (exact mass): $C_{310}H_{493}N_{65}O_{143}S_2$ (7478.2745); calculated: 1870.5759 $[M+4H]^{4+}$, 1496.6622 $[M+5H]^{5+}$, 1247.3864 $[M+6H]^{6+}$, found: 1870.5626, 1496.6545, 1247.3815.

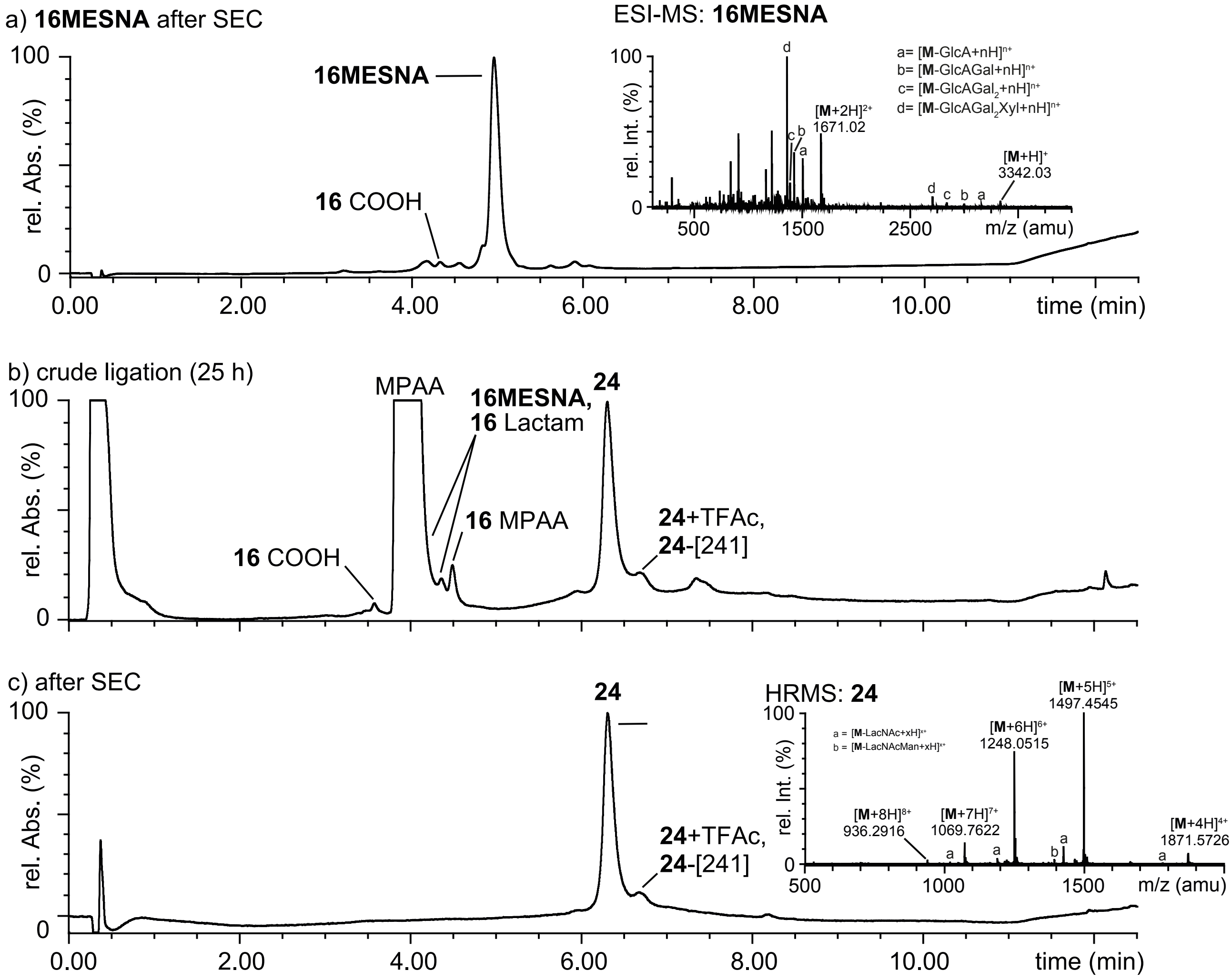

Figure S48: RP-HPLC-MS of a) purified thioester **16MESNA** after SEC; b) crude ligation mixture after 25 h; c) purified glycopeptide hydrazide 1-50 **24** after SEC.

8. Crystallization of B3GalT6 18

8.1 Overexpression and Crystallization of B3GalT6 18

Cleavage and concentration of **18**

SENP2: 11 mg/mL in 5 mM TRIS, 75 mM NaCl, 2 mM DTT, 0.1 mM EDTA,
10 % glycerol (w/v)
gel filtration buffer: 500 mM NaCl, 20 mM MES, pH 6.6

A solution of **18S** (15 mL, 1.5 mg **18S**/mL in 500 mM NaCl, 20 mM MES, pH 6.6) was concentrated to 3.5 mL in a 10 mL Amicon stirred cell (NADIR UP020 P). Subsequently, 55 µL of SENP2 (11 mg/mL in 5 mM TRIS, 75 mM NaCl, 2 mM DTT, 0.1 mM EDTA, 10 % glycerol w/v) were added and the mixture was kept at 27 °C for 48 h. The precipitate was removed by centrifugation (25000 g, 4 °, 2 min) followed by gel filtration (HiLoad 16/600 Superdex 75 pg, 1 mL/min,500 mM NaCl, 20 mM MES, pH 6.6). Protein yield after gel filtration: 5 mg of **18** (c = 0.5 mg/mL). This solution was subsequently concentrated stepwise:
A solution of **18** after gel filtration (20 mL, 0.22 mg **18**/mL in 500 mM NaCl, 20 mM MES, pH 6.6) was combined with 4 mL of buffered glycerol (60 % glycerol in 500 mM NaCl, 20 mM MES, pH 6.6) and concentrated to 500 µL (Amicon Ultra-5, MWCO 10 kDa). Protein yield: 1.5 mg **18** (c = 3 mg/mL).
To the concentrated solution of **18** (500 µL) were added 100 µL of buffered glycerol (60 % glycerol in 500 mM NaCl, 20 mM MES, pH 6.6) and concentrated to 180 µL (Amicon Ultra-0.5, MWCO 10 kDa). Protein yield: 1.2 mg **18** (c = 6.8 mg/mL).
To the concentrated solution of **18** (180 µL, 6.8 mg/mL in 20 % glycerol, 500 mM NaCl, 20 mM MES, pH 6.6) were added 4.7 µL of UDP-Gal (165 mM, 120 mg/mL in H_2O) and 0.9 µL of $MnCl_2$ (100 mM).
This solution of **18** at a concentration of 6.8 mg/ml was employed in the initial screening for crystallization conditions using the commercial Qiagen screens Classic Suite I, II and JCSG+. The nano liter pipetting was performed by a Phoenix liquid handling robot (Art Robbins) in 96-well MRC-2 crystallization plates (Molecular Dimensions). Crystallization plates were stored at 20°C and automatic imaging was performed by a RockMaker 1000 imaging system (Formulatrix, Ltd.). Suitable 3D crystals grew within one week at 20 % (w/v) PEG 4000, 20 % (v/v) 2-propanol and 100 mM trisodium citrate, pH 5.6. Before flash freezing in liquid nitrogen, these crystals were transferred into a cryo-protectant solution at the same condition supplemented with 25 % (v/v) glycerol. For protein phasing, the same cryo condition was used for soaking experiments with 0.2 mM $HgCl_2$.

X-ray diffraction data collection was performed at HZB beamline MX-14.1 (BESSY, Berlin) using a DECTRIC Pilatus 6M detector at mercury absorption edge at a wavelength of 1.0064 Å. Data processing and scaling was done by *XDSAPP*[19] suite. Phasing and initial automatic model building was done within *CCP4i* [20] by using the *Phaser/Shelx* SAD pipeline [21,22]. Here, only the peak $HgCl_2$ data set was used for heavy atom phasing. An unsoaked crystal, was used as a native processed data set for the high resolution structure refinement with *REFMAC* [23] at a maximum resolution of 1.2 Å. Data processing and structure refinement statistics are given in Table **S6**.

Table **S6**: X-ray data and refinement statistics.

**Data processing**	
Space group (symbol / no.)	C222(1) / 20
Unit cell (a,b,c / α,β,γ) [Å / °]	49.26, 77.45, 151,09 / 90, 90, 90
Resolution (High res), [Å]	50.0 – 1.2 (1.27 – 1.2
Unique reflections (High res), [Å]	108 243 (17 160)
Observations (High res), [Å]	1 403 966 (206 135)
Completness (h.r.)	99.6 (98.5)
Mutliplicity (h.r.)	13.0 (12.0)
Wilson B-factor, [Å^2]	19.58
R-meas (h.r.), [%]	11.4 (329.6)
I/sigma (h.r.)	11.5 (0.60)
CC1/2 (h.r.)	99.9 (37.7)
Processing program	XDS
Scaling program	XDS
Wavelength [Å]	1.006364
**Refinement**	
no. of amino acids (aa range)	(53 – 329)
no. of protein atoms	2256
no. of waters	378
no. of solvent atoms	14
no. of metals	1
Resolution (High res), [Å]	50.0 – 1.2 (1.231 – 1.2)
R-work (High res) [%]	16.7 (54.5)
R-free (High res) [%]	20.4 (57.6)
B-factor protein atoms, [Å^2]	13.30
B-factor metals, [Å^2]	21.71
B-factor water, [Å^2]	34.25
B-factor solvent atoms, [Å^2]	13.64
RMSD bond lengths / target [A]	0.010 / 0.012
RMSD angles / target [°]	1.897 / 1.864
RMSD planes / target [A]	0.009 / 0.020

8.2 Relative activity of 20S and 21S

Reaction buffer: 200 mM MOPS, 0.5 M NaCl, pH 7

SUMO-B4GalT7 **18S** (1.9 mg/mL) in 50 mM $NaH_2PO_4 \cdot 2\,H_2O$, 0.5 M NaCl, pH 6.6

SUMO-B3GalT6 **20S** (1.56 mg/mL) in 20 mM Mes, 0.5 M NaCl, pH 6.7

SUMO-B3GalT6 **21S** (138 mg/mL) in 20 mM Mes, 1 M NaCl, pH 6.7

Stock solution (60 µL total volume): 28.9 µL of reaction buffer (200 mM MOPS, 0.5 M NaCl, pH 7), 9.5 µL of **18S** (1.9 mg/mL), 8.8 µL of UDP-Gal (100 mg/mL in H_2O), 6 µL of BSA (10 mg/mL in reaction buffer), 6.0 µL of alkaline phosphatase (100 mU/µL in reaction buffer) and 0.8 µL $MnCl_2$ (10 mg/mL in reaction buffer) were combined and vortexed prior to use.

Glycopeptide hydrazide **A** (0.5 mg, 182.9 nmol) was dissolved in 45.7 µL of stock solution and incubated at 30 °C for 1 hour. The resulting reaction conditions were: **A** (4 mM), **18S** (0.3 mg/mL), UDP-Gal (24 mM), BSA (1 mg/mL), $MnCl_2$ (1 mM), alkaline phosphatase (10 mU/µL). After 1 hour, the reaction mixture was split into two portions of 20 µL each for the galactosylation reaction with **20S** and **21S**.

Galactosylation of **14** with **20S**:

To 20 µL of the reaction mixture 0.74 µL of **20S** and 5.92 µL of the reaction buffer were added. The mixture was incubated at 30 °C, resulting in a final reaction concentration of **14** at 3 mM.

Galactosylation of **14** with **21S**:

To 20 µL of the reaction mixture 0.84 µL of **20S** and 5.82 µL of the reaction buffer were added. The mixture was incubated at 30 °C, resulting in a final reaction concentration of **14** at 3 mM.

The reaction mixtures were analyzed by LC-MS at the specific time points:

Hydrosphere C18, S-2 µm, 2.0 x 50 mm, 120 Å, 5-20 % $MeCN/H_2O$ + 0.1 % HCOOH, 40 °C.

Hypercarb S-3 µm, 2.1 × 30 mm, 250 Å, 15-35 % $MeCN/H_2O$ +0.1% HCOOH, 80 °C.

a) comparison of enzymatic conversion with **20S** and **21S**

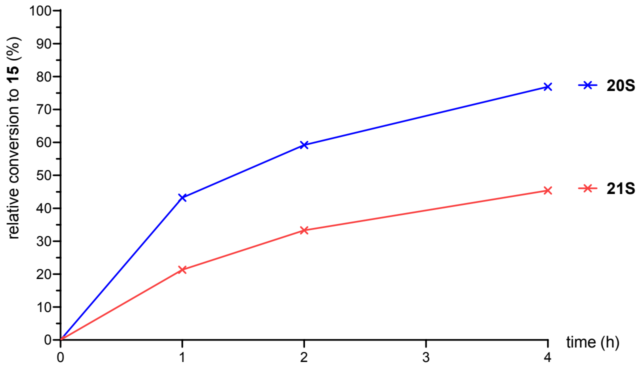

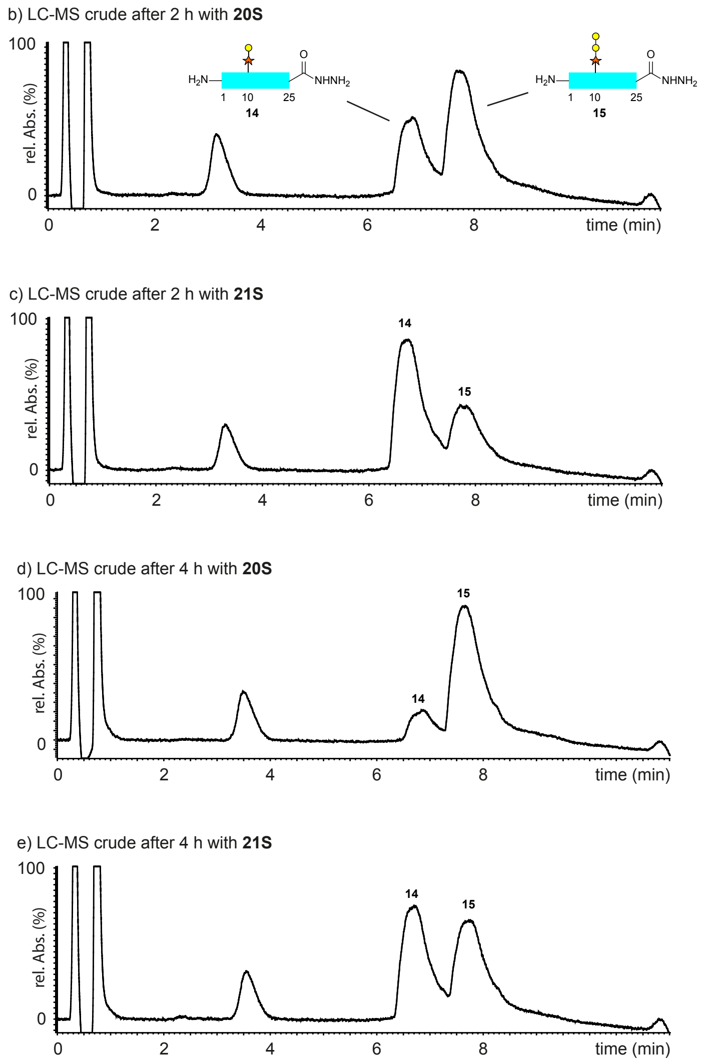

Figure S49: a-d) Galactosylation of **14** monitored by HPLC-MS using the enzymes **20S** (disulfide-linked dimer) and **21S** after 2 and 4 h; e) conversion to **15** using **20S** and **21S** plotted against reaction time (conversions were determined based on MS-intensities).

8.3 Thermal unfolding of 18S, B3GalT6 20S and 21S, 17S

Stock solutions:

SUMO-B3GlcAT1 **17S** (2 mg/mL) in 50 mM $NaH_2PO_4 \cdot 2\ H_2O$, 0.5 M NaCl, pH 6.6

SUMO-B4GalT7 **18S** (2 mg/mL) in 50 mM $NaH_2PO_4 \cdot 2\ H_2O$, 0.5 M NaCl, pH 6.6

SUMO-B3GalT6 **20S** (1.35 mg/mL) in 20 mM Mes, 1 M NaCl, pH 6.7

SUMO-B3GalT6 **21S** (1.1 mg/mL) in 20 mM Mes, 1 M NaCl, pH 6.7

CD measurements:

Prior to the measurement of the melting curves, the stock solutions of the glycosyltransferases were diluted with H_2O to the following protein concentrations: **17S** (0.2 mg/mL), **18S** (0.29 mg/mL), **20S** (0.3 mg/mL), and **21S** (0.28 mg/mL). CD at 222 nm was measured on a JASCO J-715 spectropolarimeter (JASCO Corp., Rev. 1.00) with temperature control. Data were recorded from 20-95 °C over 75 min. The melting curves for **17S**, **20S**, and **21S** were fitted using the Boltzmann sigmoid equation, while a simple linear regression was sufficient for **17S**, where no significant unfolding was observed. All analyses were performed using GraphPad Prism.

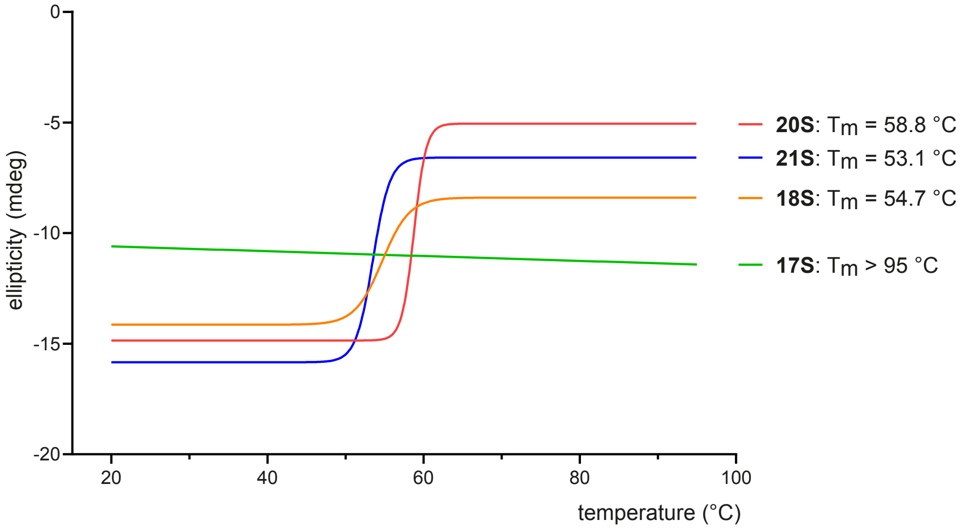

Figure S50: Thermal unfolding of the SUMOylated glycosyltransferases **17S**, **18S**, **20S** and **21S** monitored by CD-spectroscopy at 222 nm. The curves are fitted and plotted in one graph.

8.4 Sequence alignment of B3GalT6 from various animal species

```
tr|A0A2S2QV21|A0A2S2QV21_9HEMI     1  ............................................MF.............L
sp|Q9N491|SQV2_CAEEL               1  ...................................MRFY.........RTYL.......L
tr|Q257A1|Q257A1_CAEBR             1  ...................................MRFYR...LSF..QTYL.......L
tr|A0A836EW34|A0A836EW34_9HYME     1  ...........................MTYLRLDLMPKLQ...SIMRRR..FIARA....
tr|A0A8X6YCK5|A0A8X6YCK5_9ARAC     1  .......................................MNNGKMRRHTLKS........
tr|A0A5B7E752|A0A5B7E752_PORTR     1  MLGDIAGHFDSKLSEGRRAAGNPAIVLNKRSNVNVQNMMRRGGGFWLCRRPLLSAVQVLA
tr|Q256Y9|Q256Y9_TETNG                ............................................................
tr|A0A4Z2F2N5|A0A4Z2F2N5_9TELE     1  ...................................MNLYR...SACRHKTAL........
sp|Q91Z92|B3GT6_MOUSE              1  ...................................MKVFR...RAWRHRVAL........
tr|D3ZQC1|D3ZQC1_RAT               1  ...................................MKVFR...RAWRHRVVL........
sp|Q96L58|B3GT6_HUMAN              1  ...................................MKLLR...RAWRRRAAL........
tr|F1MVH6|F1MVH6_BOVIN             1  ...................................MKLLR...RAWRHRTAL........
tr|I3LU42|I3LU42_PIG               1  ...................................MRLLR...RAWRHRTAL........
tr|A0A1L8FM74|A0A1L8FM74_XENLA     1  ...................................MNLVR...VICRHKTAL........
tr|A0A151N4M8|A0A151N4M8_ALLMI     1  ...................................MKLLR...LLCRHKTAL........
tr|A0A851X0E2|A0A851X0E2_CORMO     1  ...................................MKALR...RLSRHRTAL........
tr|Q257A2|Q257A2_AEDAE             1  .........................................................MLP
tr|Q256Z6|Q256Z6_DROER             1  ...................................MRRLN..........NL...VTFFT
tr|Q256Z3|Q256Z3_DROSE             1  ...................................MRRLN..........NL...VTFFT
```

```
tr|A0A2S2QV21|A0A2S2QV21_9HEMI     4  NQKRLIY..........FFLANLSFILGCTLTLFFLHTTT....................
sp|Q9N491|SQV2_CAEEL              10  VAGA..FCSLCTL..........AVIFNCGWDDSPPATPS.......AIN..........
tr|Q257A1|Q257A1_CAEBR            14  VAGA..FCSICTL..........AVIFNCGWEDNNNI.......................
tr|A0A836EW34|A0A836EW34_9HYME    25  NVLAFLFAIVFVFLCLRYLP.....ERECQ..............................
tr|A0A8X6YCK5|A0A8X6YCK5_9ARAC    14  ...IFLYCIYIWLLAVGYFLGVLHSFHRCNENTSILNPSV....................
tr|A0A5B7E752|A0A5B7E752_PORTR    61  CVGAFMLGSLVTLMSIDP...MRCDIHQCTEKIKRTDVED....................
tr|Q256Y9|Q256Y9_TETNG             1  ............V...........FLAKCTSETLKQDQQDPPRIVPRAKFVHSH......
tr|A0A4Z2F2N5|A0A4Z2F2N5_9TELE    15  VVGSVSLFAVVLL...........FLAKCTSETPRPDRRDAPGLEPRAAAPRAPPEPRDP
sp|Q91Z92|B3GT6_MOUSE             15  GLGGLAFCGTTLL...........YLARCASEGETPSASGA.......A...........
tr|D3ZQC1|D3ZQC1_RAT              15  GLGGLAFCSTTLL...........YLARCASEGETPSASGA.......A...........
sp|Q96L58|B3GT6_HUMAN             15  GLGTLALCGAALL...........YLARCAAEPGDPRAMSG.......R...........
tr|F1MVH6|F1MVH6_BOVIN            15  GLGGLAMGGVALL...........YLARCAAPPDSAL.SGP.......A...........
tr|I3LU42|I3LU42_PIG              15  GLGCLALGGATLL...........YLARCAAPPAP.........................
tr|A0A1L8FM74|A0A1L8FM74_XENLA    15  GLGAVSVFLVVLL...........YLAKCTSESLKPASP.R.......ALPYQQAS....
tr|A0A151N4M8|A0A151N4M8_ALLMI    15  GLGGLSLFAVVLL...........YLA.................................
tr|A0A851X0E2|A0A851X0E2_CORMO    15  GLGGLSLCAAVLL...........YLAKCTSEGLRPLPAPP.......ALPHSQPG....
tr|Q257A2|Q257A2_AEDAE             4  LFCSFLAGVCLTV........LIGGTRQTCE.............................
tr|Q256Z6|Q256Z6_DROER            13  AITAFFFGSFITK........ILNSVDQCPA.............................
tr|Q256Z3|Q256Z3_DROSE            13  AITAFFFGSFITK........ILSSVDQCPA.............................
```

```
tr|A0A2S2QV21|A0A2S2QV21_9HEMI    34  ...............FKSITLPKEPRFKLLVLVISAVKNQNRRDAIRETWAQAK......
sp|Q9N491|SQV2_CAEEL              41  .....GGGSNA....PLISSPTNLPETFLYISILTSPNETERRQNVRDTWFRLS..T...
tr|Q257A1|Q257A1_CAEBR            39  ................ALASTAHLSETFLFVSVLSSPNETERRQNVRETWFRLS..A...
tr|A0A836EW34|A0A836EW34_9HYME    50  ...............SNRQTLGSKLKHRLIALILSSPDNLERRNTIRKTWLAEH......
tr|A0A8X6YCK5|A0A8X6YCK5_9ARAC    51  .............TSDSTELLIQKHAAFFVVVIFSAPANYERRLAIRKTWLSTK......
tr|A0A5B7E752|A0A5B7E752_PORTR    98  ........VNSWLGMWGKGNRGAEKSVFLVIVMLSAPANKEQREVIRQTWLSEE......
tr|Q256Y9|Q256Y9_TETNG            32  ............VEVRNAPSISKDFSVFLVVLITTGPKYTERRSIIRSTWLTKR......
tr|A0A4Z2F2N5|A0A4Z2F2N5_9TELE    64  PEPRDPPEPRDPPEPRDPPEPAKELAAFLVVLITSGPKYTERRSIIRSTWLARR......
sp|Q91Z92|B3GT6_MOUSE             46  ...............R......PRAKAFLAVLVASAPRAVERRTAVRSTWLAPE.RR...
tr|D3ZQC1|D3ZQC1_RAT              46  ...............R......TRAKAFLAVLVASAPRAVERRTAVRSTWLAQE.RR...
sp|Q96L58|B3GT6_HUMAN             46  ...............SPPPPAPARAAAFLAVLVASAPRAAERRSVIRSTWLA...RR...
tr|F1MVH6|F1MVH6_BOVIN            45  ...............APAPVGPARAAAFLAVLVASAPRAAERRSVVRSTWLAA..RR...
tr|I3LU42|I3LU42_PIG              39  ...............APAPAAQARAVAFLAVLVASAPRAAERRSVVRSTWLAA..RR...
tr|A0A1L8FM74|A0A1L8FM74_XENLA    52  ............HRQ.QQHGAEKSVSAFLVVLIASGPKYSERRSIIRSTWLSGVKSR...
tr|A0A151N4M8|A0A151N4M8_ALLMI    31  ..................................NGPKYTERRSIIRSTWLSTA.GR...
tr|A0A851X0E2|A0A851X0E2_CORMO    53  ............RGARAAPPPAPEGSAFVAVVVMSGPKYSERRSIIRSTWMAAA.RQ...
tr|Q257A2|Q257A2_AEDAE            27  ...............SVGRIYEPENSYFLMLLIVSAPGNVERRNAIRETYLNLRPRMLNE
tr|Q256Z6|Q256Z6_DROER            36  .............HRSRIPHLEPHPNLFLMVLVLSAPHNAEERQAMRSTWLASAGQSLAQ
tr|Q256Z3|Q256Z3_DROSE            36  .............HRSRIPHLEPHPKLFLMVLVLSAPHNSDERNAMRSTWLANAGQSLTQ
```

```
tr|A0A2S2QV21|A0A2S2QV21_9HEMI    73  .....................................................DDVEVRF
sp|Q9N491|SQV2_CAEEL              87  ..................................................KGPSVFIAKF
tr|Q257A1|Q257A1_CAEBR            78  ..................................................KGPSVFIAKF
tr|A0A836EW34|A0A836EW34_9HYME    89  ....................................................DATVKHFF
tr|A0A8X6YCK5|A0A8X6YCK5_9ARAC    92  ...................................................GNLSIKHFF
tr|A0A5B7E752|A0A5B7E752_PORTR   144  ....................................................KADTLHFF
tr|Q256Y9|Q256Y9_TETNG            74  ....................................................DSDVLARF
tr|A0A4Z2F2N5|A0A4Z2F2N5_9TELE   118  ....................................................DADVLARF
sp|Q91Z92|B3GT6_MOUSE             81  ..................................................GGPEDVWARF
tr|D3ZQC1|D3ZQC1_RAT              81  ..................................................GGPKDVWARF
sp|Q96L58|B3GT6_HUMAN             85  ..................................................GAPGDVWARF
tr|F1MVH6|F1MVH6_BOVIN            85  ..................................................GGPGDVWARF
tr|I3LU42|I3LU42_PIG              79  ..................................................GGPGDVWARF
tr|A0A1L8FM74|A0A1L8FM74_XENLA    96  ..................................................AEHGDVWARF
tr|A0A151N4M8|A0A151N4M8_ALLMI    53  ..................................................PPHEDVWCRF
tr|A0A851X0E2|A0A851X0E2_CORMO    97  ..................................................APHGHVWSRF
tr|Q257A2|Q257A2_AEDAE            72  SYQ.EEAIYVPLYDGDTGQLQLESVQKQRELLNGYRRWQEKKIKNI...KVINFKVKTLF
tr|Q256Z6|Q256Z6_DROER            83  PYLPEELIYLPAFNA.NGHMQMELVAEQASRLREYTNWQKTLLTEDPPKTQRRITVKHVF
tr|Q256Z3|Q256Z3_DROSE            83  PYLPEELIYLPTFNA.QGHLQVELVAEQASRLRQYTNWQQSLLTEDPPKTKRLITVKHVF
```

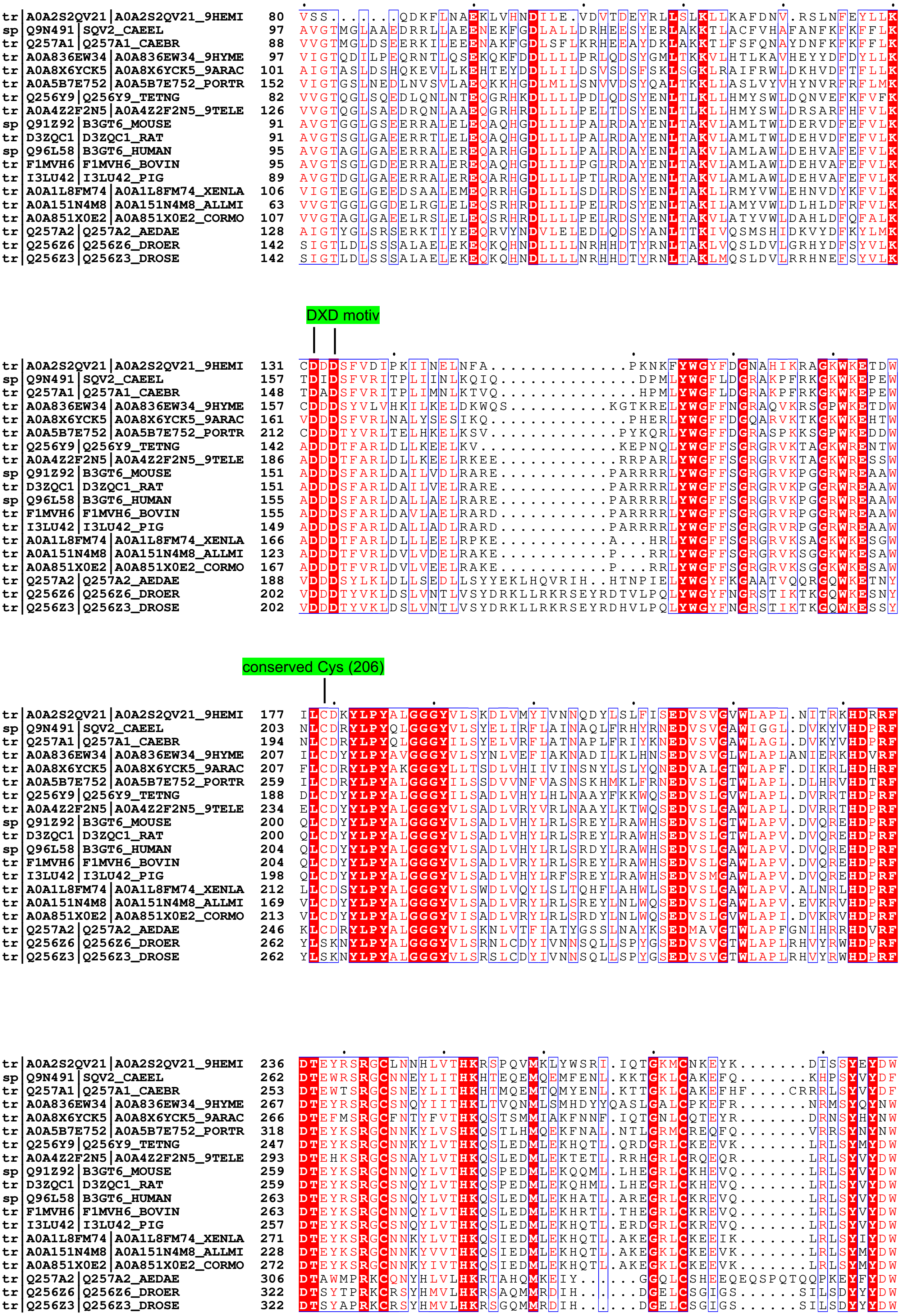
DXD motiv
conserved Cys (206)

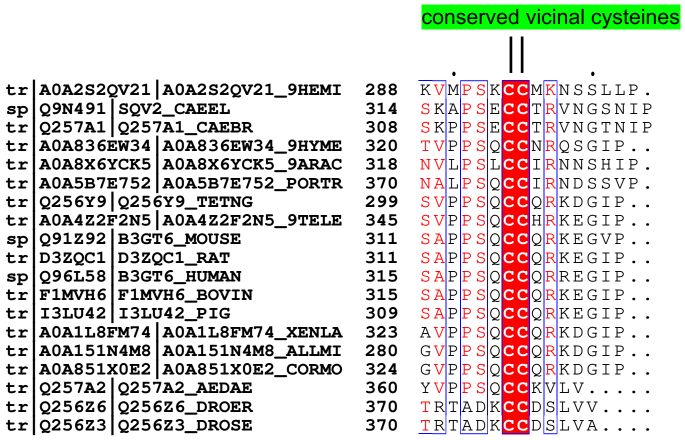

Figure S51: Sequence alignment of selected B3GalT6 proteins from various species: 9HEMI: *Sipha flava*; CAEEL: *Caenorhabditis elegans*; CAEBR: *Caenorhabditis briggsae*; 9HYME: *Acromyrmex heyeri*; 9ARAC: *Trichonephila inaurata madagascariensis*; PORTR: *Portunus trituberculatus*; TETNG: *Tetraodon nigroviridis*; 9TELE: *Liparis tanakae*; MOUSE: *Mus musculus*; RAT: *Rattus norvegicus*; HUMAN: *Homo sapiens*; BOVIN: *Bos taurus*; PIG: *Sus scrofa*; XENLA: *Xenopus laevis*; ALLMI: *Alligator mississippiensis*; CORMO: *Corvus moneduloides*; AEDAE *Aedes aegypti*; DROER: *Drosophila erecta*; DROSE: *Drosophila sechellia*. For the sequence alignment of select B3GalT6 sequences the freely accessible webtool ESPript 3.0[24] was used: https://espript.ibcp.fr/ESPript/ESPript/

9. NMR Spectra:

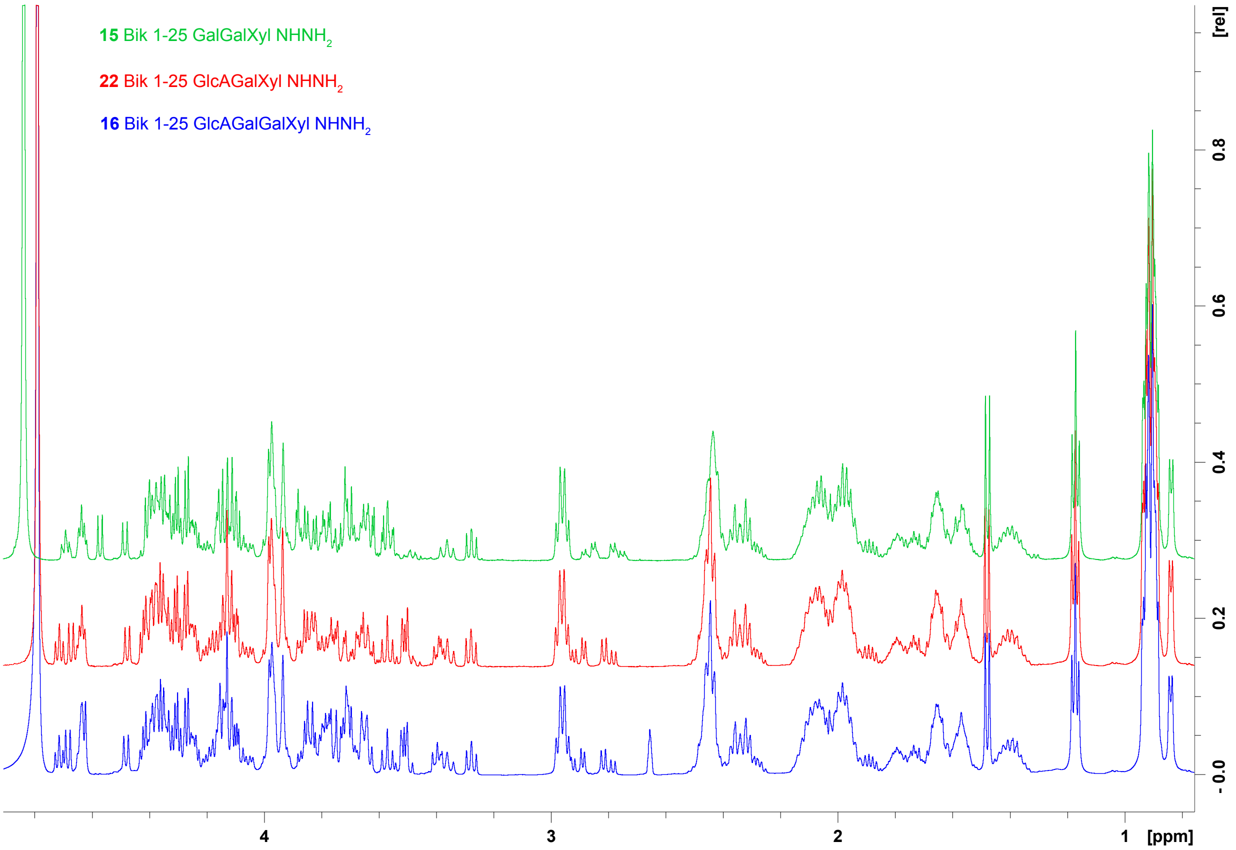

1H-NMR (D_2O) overlay of glycopeptides **15**, **22** and **16**

HSQC of glycopeptide **15**

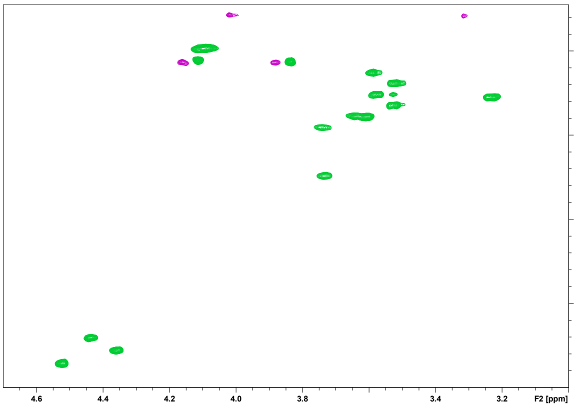

HSQC of glycopeptide **16**

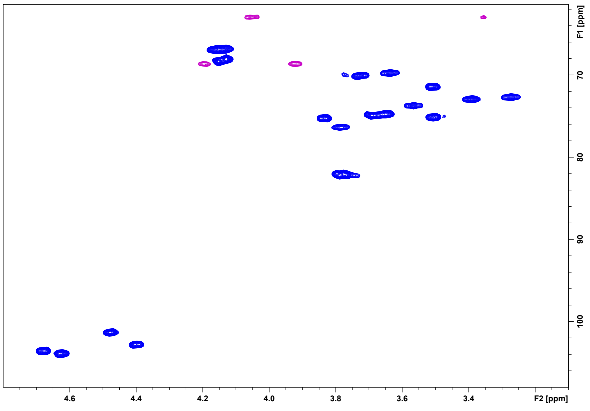

HSQC of glycopeptide **22**

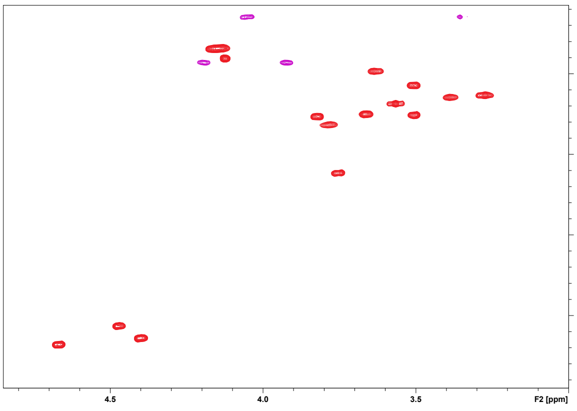

overlay of HSQC spectra of glycopeptides **15**, **16** and **22**

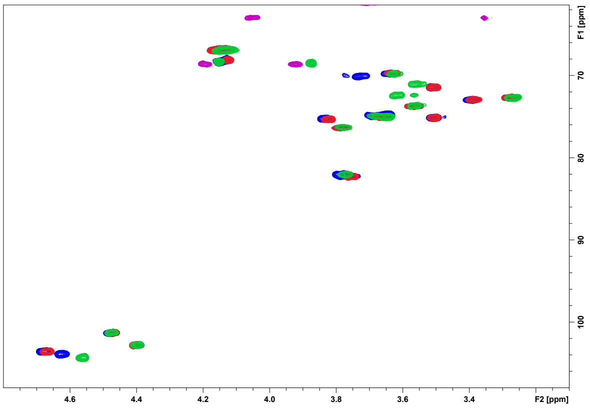

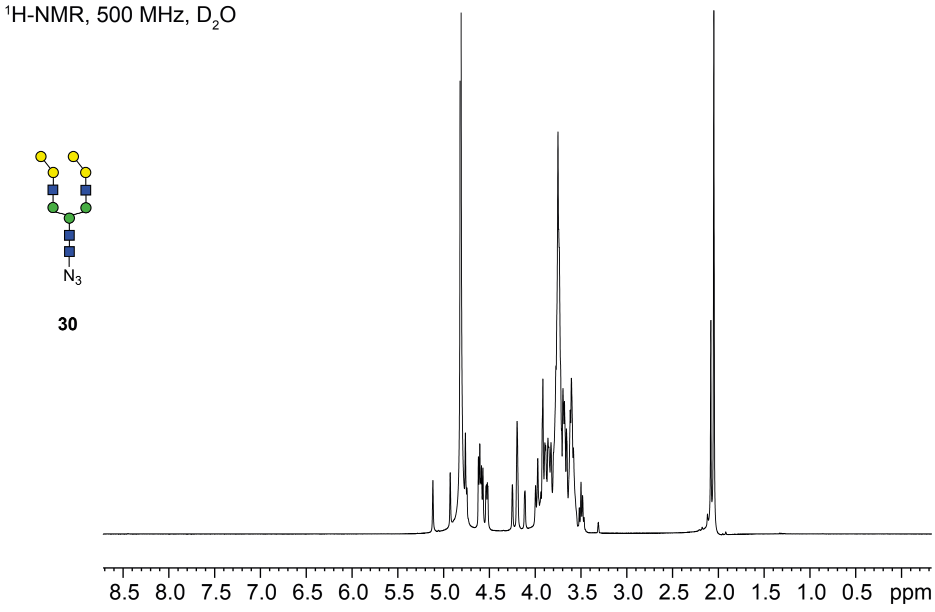

1H-NMR, 500 MHz, D2O
N3
30
8.5 8.0 7.5 7.0 6.5 6.0 5.5 5.0 4.5 4.0 3.5 3.0 2.5 2.0 1.5 1.0 0.5 ppm

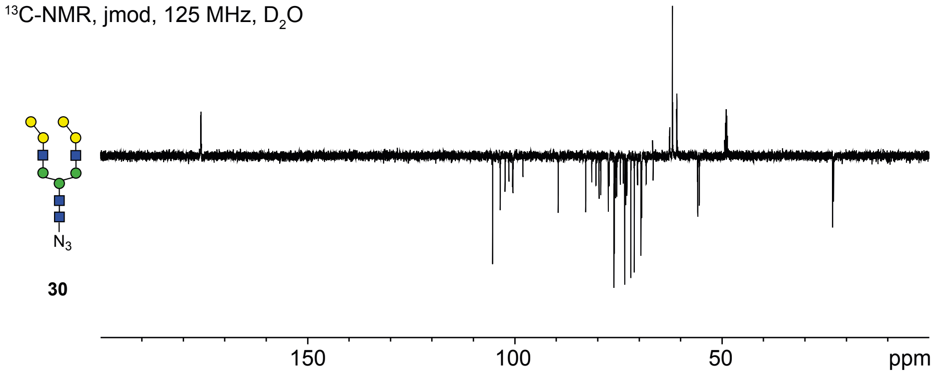

13C-NMR, jmod, 125 MHz, D2O
N3
30
150
100
50
ppm

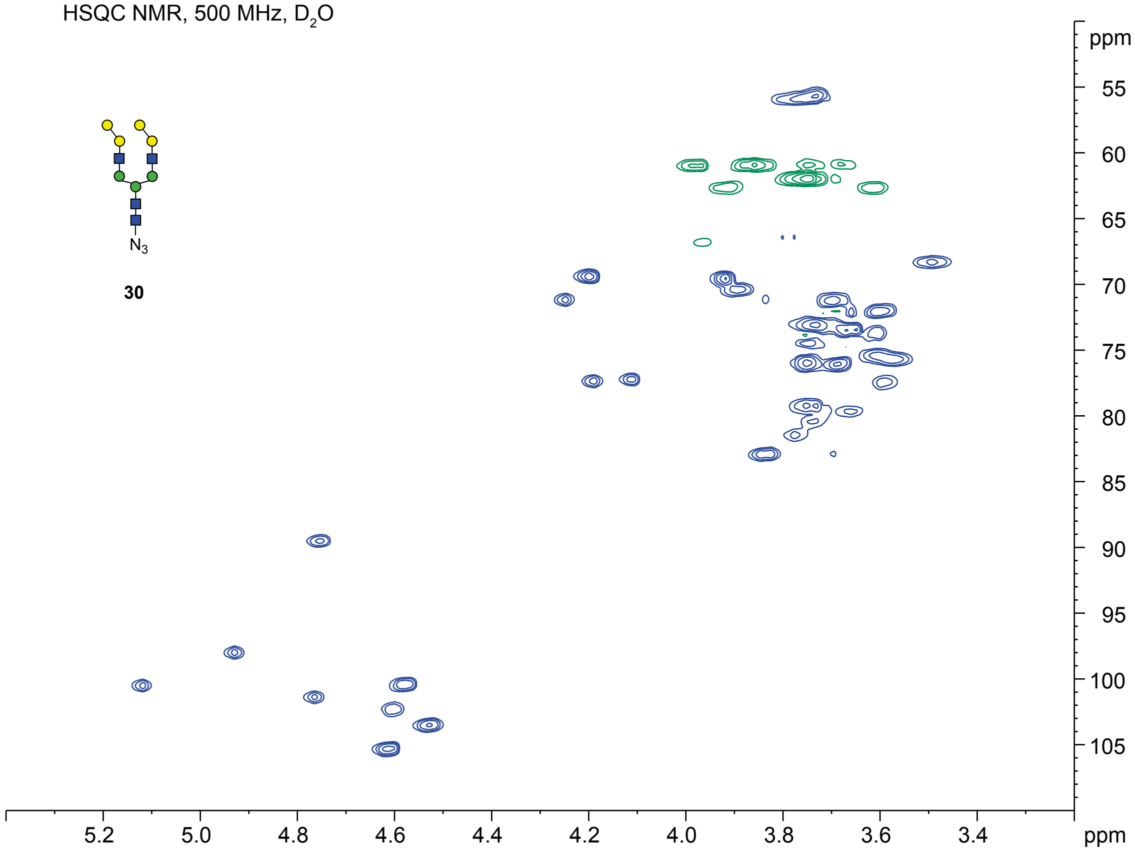

HSQC NMR, 500 MHz, D2O
N3
30
ppm
55
60
65
70
75
80
85
90
95
100
105
5.2 5.0 4.8 4.6 4.4 4.2 4.0 3.8 3.6 3.4 ppm

10. References:

[1] C. Heinlein, D. Varon Silva, A. Tröster, J. Schmidt, A. Gross, C. Unverzagt, *Angew. Chem. Int. Ed.* **2011**, 50, 6406 (see supplement pages 17-18).

[2] S. Eissler, M. Kley, D. Bächle, G. Loidl, T. Meier, D. Samson, *J. Pept. Sci.* **2017**, 23, 757–762.

[3] W. C. Chan, P. D. White, *Eds.*, *Fmoc Solid Phase Peptide Synthesis: A Practical Approach*, Oxford University Press, New York, **2000**.

[4] N. L. Benoiton, Y. C. Lee, R. Steinaur, F. M. Chen, *Int. J. Pept. Protein Res.* **1992**, *40*, 559-566.

[5] T.-Y. Huang, M. M. L. Zulueta, S.-C. Hung, *Org. Lett.* **2011**, *13*, 1506–1509

[6] P. J. Knerr, W. A. van der Donk, *J. Am. Chem. Soc.* **2012**, *134*, 7648–7651

[7] P. C. Bulman Page, Y. Chan, J. Liddle, M. R. J. Elsegood, *Tetrahedron* **2014**, *70*, 7283–7305.

[8] J. Zhang, P. Kováč, *J. Carbohydr. Chem.* **1999**, *18*, 461–469.

[9] M. Mori, Y. Ito, T. Ogawa, *Carbohydr. Res.* **1990**, *195*, 199–224.

[10] Y. Nakahara, S. Ando, Y. Ito, H. Hojo, Y. Nakahara, *Biosci. Biotechnol. Biochem.* **2001**, *65*, 1358–1368.

[11] Y. Zhang, S. M. Muthana, J. J. Barchi, Jr., J. C. Gildersleeve, *Org. Lett.* **2012**, *14*, 3958-3961.

[12] W. König, R. Geiger, *Chem. Ber.* **1970**, *103*, 2034-2040.

[13] Y. Kajihara, A. Yoshihara, K. Hirano, N. Yamamoto, *Carbohydr. Res.* **2006**, *341*, 1333-1340.

[14] V. Ullmann, M. Rädisch, I. Boos, J. Freund, C. Pöhner, S. Schwarzinger, C. Unverzagt, *Angew. Chem. Int. Ed.* **2012**, *51*, 11566-11570.

[15] R. Almeida, S. B. Levery, U. Mandel, H. Kresse, T. Schwientek, E. P. Bennett, H. Clausen, *J. Biol. Chem.* **1999**, *274*, 26165-26171.

[16] K. W. Moremen, A. Ramiah, M. Stuart, J. Steel, L. Meng, F. Forouhar, H. A. Moniz, G. Gahlay, Z. Gao, D. Chapla, S. Wang, J. Y. Yang, P. K. Prabhakar, R. Johnson, M. D. Rosa, C. Geisler, A. V. Nairn, J. Seetharaman, S. C. Wu, L. Tong, H. J. Gilbert, J. LaBaer, D. L. Jarvis, *Nat. Chem. Biol.* **2018**, *14*, 156-162.

[17] L. C. Pedersen, K. Tsuchida, H. Kitagawa, K. Sugahara, T. A. Darden, M. Negishi, *J. Biol. Chem.* **2000**, *275*, 34580-34585.

[18] a) D. J. Harvey, *J. Am. Soc. Mass Spectrom.* **2005**, *16*, 631-646; b) D. J. Harvey, *Mass Spectrom. Rev.* **2020**, *39*, 586-679

[19] M. Krug, M. S. Weiss, U. Heinemann, U. Mueller, *J. Appl. Cryst.* **2012**, *45*, 568–572.

[20] M. D. Winn, C. C. Ballard, K. D. Cowtan, E. J. Dodson, P. Emsley, P. R. Evans, R. M. Keegan, E. B. Krissinel, A. G. W. Leslie, A. McCoy, S. J. McNicholas, G. N. Murshudov, N. S. Pannu, E. A. Potterton, H. R. Powell, R. J. Read, A. Vagin, K. S. Wilson, *Acta Cryst. D* **2011**, *67*, 235–242.

[21] A. J. McCoy, R. W. Grosse-Kunstleve, P. D. Adams, M. D. Winn, L. C. Storoni, R. J. Read, *J. Appl. Crystallogr.* **2007**, *40*, 658–674.

[22] T. R. Schneider, G. M. Sheldrick, *Acta Crystallogr. D Biol. Crystallogr.* **2002**, *58*, 1772–1779.

[23] O. Kovalevskiy, R. A. Nicholls, F. Long, A. Carlon, G. N. Murshudov, *Acta Cryst. D* **2018**, *74*, 215–22.

[24] X. Robert, P. Gouet, *Nucleic Acids Res.* **2014**, *42*, W320-324.
